# Bisphosphate nucleotidase 1 promotes progression and docetaxel resistance in triple-negative breast cancer via STUB1-mediated destabilization of LIMA1

**DOI:** 10.1038/s41419-025-08245-0

**Published:** 2026-01-15

**Authors:** Yun-Xiao Ling, Lisa Andriani, Shao-Ying Yang, Qian Zhao, Min-Ying Huang, Yin-ling Zhang, Fang-Lin Zhang, Zhi-Min Shao, Da-Qiang Li, Guang-Yu Liu

**Affiliations:** 1 Shanghai Key Laboratory of Breast Cancer, Department of Breast Surgery, Fudan University Shanghai Cancer Center, Shanghai, China; 2 Department of Oncology, Shanghai Medical College, Fudan University, Shanghai, China; 3 Cancer Institute, Shanghai Medical College, Fudan University, Shanghai, China; 4 Institutes of Biomedical Sciences, Fudan University, Shanghai, China

**Keywords:** Oncogenes, Protein-protein interaction networks

## Abstract

Triple-negative breast cancer (TNBC) is the most aggressive subtype of breast cancer without effective targeted therapies. Integrative analysis of transcriptomic and proteomic datasets of TNBC in our center revealed that bisphosphate nucleotidase 1 (BPNT1), a member of inositol monophosphatase superfamily with poorly characterized functional and mechanistic roles in human cancer, was abnormally upregulated in TNBC and its high expression was associated with poor patient prognosis. Loss- and gain-of-function assays revealed that BPNT1 acted as a novel oncogenic driver to promote TNBC cell proliferation, migration, invasion in vitro and to accelerate xenograft tumor growth and lung metastasis in mice. Mechanistically, BPNT1 recruited E3 ubiquitin ligase STUB1 (STIP1 homology and U-box containing protein 1) to induce proteasomal degradation of tumor suppressor protein LIMA1 (LIM domain and actin binding 1), thus promoting the epithelial-mesenchymal transition process and TNBC progression. Notably, re-expression of LIMA1 in BPNT1-overexpressing cells partially attenuated BPNT1-driven EMT and malignant phenotypes of TNBC cells. Furthermore, knockdown of BPNT1 enhanced the sensitivity of TNBC cells to the chemotherapeutic agent docetaxel. Collectively, these findings uncover a previously unknown role of the BPNT1-STUB1-LIMA1 axis in progression and docetaxel resistance in TNBC, and highlight BPNT1 as a potential therapeutic target for patients with TNBC.

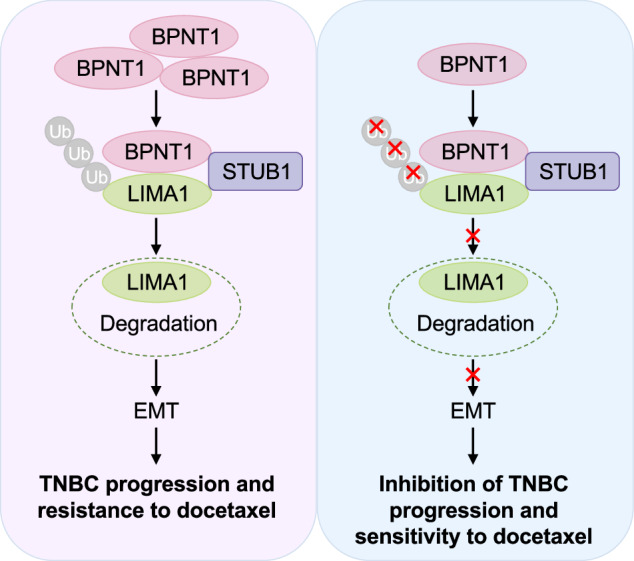

## Introduction

Breast cancer (BC) remains a leading cause of female cancer mortality worldwide [1], with prognosis closely linked to molecular subtypes. Triple-negative breast cancer (TNBC), lacking estrogen/progesterone receptors and HER2 (~10–20% of invasive BCs), shows aggressive behavior with high relapse, metastasis, and poor survival [2]. Lack of actionable targets limits TNBC therapy, leaving cytotoxic chemotherapy as the mainstay [3]. Docetaxel (DTX), a microtubule-targeting taxane, is a cornerstone chemotherapy for advanced TNBC [4]; however, resistance to DTX remains a major clinical challenge [5, 6]. Consequently, delineating previously unrecognized targets that mediate TNBC progression and chemoresistance is a pressing research priority.

Bisphosphate nucleotidase 1 (BPNT1), an inositol monophosphatase family member, hydrolyzes 3′-phosphoadenosine 5′-phosphate (PAP) to 5’-adenosine monophosphate (5’-AMP) [7–9]. Evidence from multiple model organisms indicates that disruption of BPNT1 leads to the toxic accumulation of PAP and diverse pathologies such as liver failure, neuronal dysfunction, iron-deficiency anemia, anasarca, and hemochromatosis [8, 10–13]. Bioinformatic analyses also link BPNT1 dysregulation to glioma [14], gastric cancer [15], and head and neck squamous cell carcinoma [16].

Given the aggressive nature and therapeutic challenges of TNBC, our center (Fudan University Shanghai Cancer Center) has established a robust transcriptomic cohort comprising 465 TNBC patients [17]. Integrative analysis of our recently published transcriptomic [17] and proteomic [18] datasets of TNBC revealed that BPNT1 is significantly overexpressed in TNBC tissues compared to matched paracancerous tissues and its elevated expression correlates with poor patient prognosis.

LIM domain and actin binding 1 (LIMA1/EPLIN) is a cytoskeletal scaffold governing actin dynamics, motility, and adhesion [19, 20], and functions as an EMT/metastasis suppressor often downregulated in cancer [21–23]. Recent studies provide evidence that epidermal growth factor [24], Rab40-Cullin5 complex [25], and deubiquitinase USP44 [26] affect the stability of LIMA1. LIMA1 is regulated via phosphorylation/dephosphorylation by extracellular signal-regulated kinase [27] and phosphatase CDC14 [28], respectively. Nevertheless, mechanisms leading to reduced LIMA1 in human cancers are incompletely defined.

In this study, we demonstrate that BPNT1 promotes TNBC progression and DTX resistance. Mechanistically, BPNT1 recruits the E3 ligase STUB1 to trigger ubiquitin-proteasomal degradation of the tumor suppressor LIMA1, driving EMT. Silencing BPNT1 sensitizes TNBC cells to DTX. These findings uncover a novel BPNT1-STUB1-LIMA1 axis in TNBC pathogenesis and chemoresistance, positioning BPNT1 as a therapeutic target.

## Materials and methods

### Cell culture and reagents

Human TNBC cell lines, MCF10A, and HEK293T were obtained from the Shanghai Key Laboratory of Breast Cancer (FUSCC) and authenticated by short tandem repeat profiling (STR). MCF10A cells were cultured as described in published protocols [29], while other lines were maintained in DMEM with 10% FBS and 1% penicillin/streptomycin at 37 °C, 5% CO₂.

### Clinical samples and data

RNA-sequencing (RNA-Seq) [17] and quantitative proteomic [18] data derive from the published FUSCC-TNBC cohort. Paired TNBC and adjacent normal tissues were collected from treatment-naïve FUSCC patients under approved ethics protocols.

### DNA plasmids construction and transfection

Short hairpin RNA (shRNA) targeting BPNT1 or STUB1 (sequences in Table S1) were cloned into pLKO.1 vectors. BPNT1, LIMA1, and STUB1 cDNAs (primers in Table S2) were cloned into pCDH-Flag or pLVX-HA vectors. Transfections used Neofect per established protocols [29, 30].

### Immunoblotting and immunoprecipitation assays

Proteins were extracted with RIPA buffer, quantified (BCA assay), separated by SDS-PAGE, and transferred to PVDF membranes. After blocking with 5% BSA, membranes were incubated with primary antibodies overnight at 4 °C, followed by secondary antibodies, and visualized by ECL. Immunoprecipitation (IP) and liquid chromatography-tandem mass spectrometry (LC-MS/MS) assays were performed as described [29, 30]. Antibodies are listed in Table S3.

### RNA extraction and RT-qPCR

RNA was extracted using RNAiso Plus, reverse transcribed using HiScript III RT SuperMix kit, and analyzed by Real-time quantitative PCR (RT-qPCR) using the 2^−ΔΔCT^ method. Primer sequences are provided in Table S4.

### Cell proliferation and colony formation assays

Cells (1000/well) were plated in 96-well plates and treated with DMSO or DTX; viability was measured by CCK-8 (A450). For colony assays, 1000 cells/well in 6-well plates were treated and after 12–14 days fixed and stained with 0.2% crystal violet.

### Cell migration and invasion assays

Transwell migration/invasion assays used 2–5 × 10⁴ cells in serum-free medium (uncoated inserts for migration; Matrigel for invasion). After 16–18 h, cells on the underside were fixed, stained with 0.2% crystal violet, and counted.

### Xenograft tumor models and treatment

All animal experiments were approved by the Animal Experiments Committee of FUSCC. All group allocations were randomized. Mammary fat pads of 6-week-old female BALB/c nude mice (*n* = 8–10) received 3 × 10⁶ cell inoculations for tumorigenesis evaluation. Upon reaching mean tumor volumes of 100 mm³, cohorts underwent biweekly intraperitoneal injections of DTX (20 mg/kg in DMSO) or vehicle control across six administrations. Tumor dimensions underwent triweekly caliper measurement, with volumes computed via established formula [29]. After euthanasia, xenograft tumors from the mice were dissected and weighed. To perform lung metastasis assays, 1 × 10^6^ cells were injected into the tail vein of 6-week-old female BALB/c nude mice. Nude mice were euthanized when they exhibited weight loss of at least 10% or developed dysphagia or cachexia, and their lungs were removed and evaluated.

### Immunofluorescent staining and ubiquitination assays

Cells grown on coverslips were fixed with 4% PFA, permeabilized with 0.1% Triton X-100, blocked with 5% BSA, incubated with primary antibodies overnight (4 °C), secondary antibodies (1 h, RT), mounted with DAPI, and imaged by confocal microscopy. Ubiquitination was assessed following established methodologies [29, 30]. HEK293T cells underwent individual or combinatorial transfection with designated plasmids. Post 48 h incubation, 6 h MG-132 exposure (10 μM) preceded denaturing lysis and IP-based ubiquitination detection. Reagents used in this study are listed in Table S5.

### Statistical analysis

Quantitative data (≥3 independent experiments) are mean ± SD. Statistical comparisons used two-tailed t-tests or one-way ANOVA (GraphPad Prism v9.4); significance thresholds: *p < 0.05, **p < 0.01, ***p < 0.001, ****p < 0.0001 (ns, nonsignificant). GDSC database (https://www.cancerrxgene.org/) facilitated gene-drug association analysis.

## Results

### BPNT1 is aberrantly overexpressed in TNBC tissues and its high expression is associated with poor patient prognosis

**Fig. 1:**
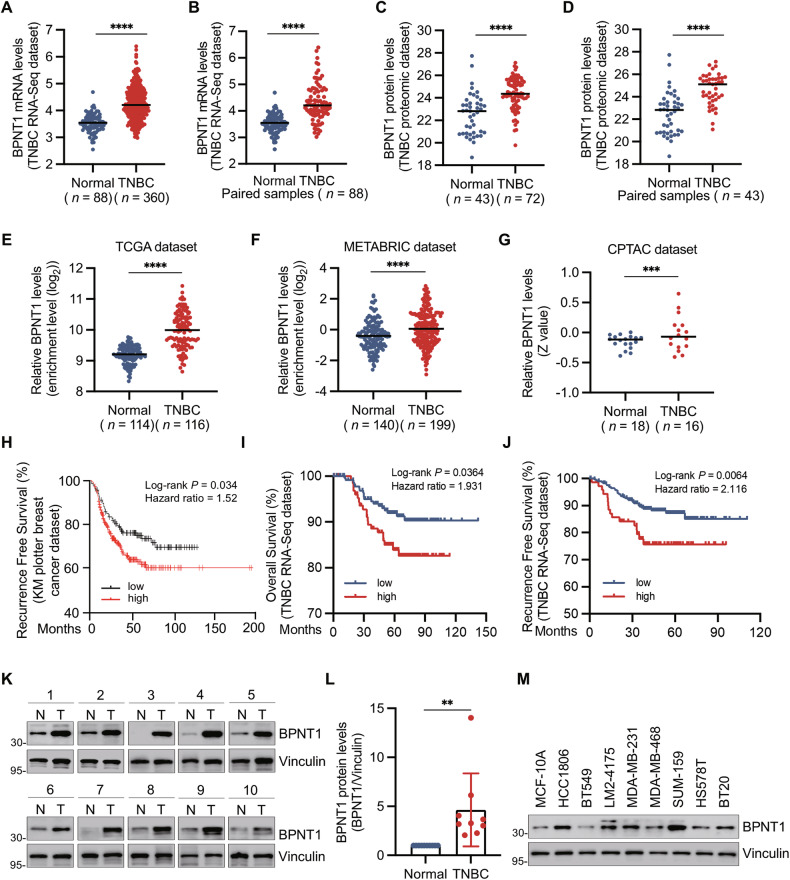
BPNT1 is overexpressed in TNBC tissues and is associated with poor disease prognosis. **A**, **B** BPNT1 mRNA levels in 360 TNBC tissues and 88 adjacent normal tissues in the FUSCC-TNBC RNA-Seq dataset. **C**, **D** BPNT1 protein levels in 72 TNBC tissues and 43 adjacent normal tissues in the FUSCC-TNBC proteomic dataset. **E** Analysis of BPNT1 mRNA levels in 114 normal tissues and 116 TNBC tissues in the TCGA dataset. **F** Analysis of BPNT1 mRNA levels in 140 normal tissues and 199 TNBC tissues in the METABRIC dataset. **G** Analysis of BPNT1 protein levels in 18 normal tissues and 16 TNBC tissues in the CPTAC dataset. **H** Kaplan–Meier analysis of recurrence-free survival of TNBC patients with high and low expression levels of BPNT1 in the Kaplan–Meier Plotter dataset (https://kmplot.com/analysis/). Kaplan–Meier analysis of overall survival (**I**) and recurrence-free survival (**J**) of TNBC patients with high and low BPNT1 expression. **K**, **L** Immunoblotting analysis of BPNT1 protein expression levels in 10 pairs of TNBC tissues and matched normal breast tissues. Quantitative results of relative BPNT1 protein expression levels (BPNT1/Vinculin) are shown in (**L**). N normal, T tumor. **M** Immunoblotting analysis of BPNT1 protein expression levels in human mammary epithelial cell line MCF10A and eight representative human TNBC cell lines. Quantitative results of relative BPNT1 protein expression levels are shown in Fig. S1B.

To identify therapeutic targets underlying TNBC progression, we performed integrative analyses of transcriptomic [17] and proteomic [18] datasets from our institutional TNBC cohorts. BPNT1 was abnormally elevated in paired and unpaired TNBC versus adjacent normal tissues (Fig. 1A–D), with highest levels in the BLIS subtype (Supplementary Fig. S1A). Consistent results were observed in external cohorts (TCGA, METABRIC, CPTAC), confirming BPNT1 elevation in TNBC (Fig. 1E–G). Kaplan–Meier survival analysis demonstrated significantly reduced recurrence-free survival (RFS) in TNBC patients with elevated BPNT1 expression (Fig. 1H). Corroborating this, FUSCC-TNBC RNA-Seq data [17] linked heightened BPNT1 levels to poorer RFS and overall survival (OS) outcomes (Fig. 1I, J). Immunoblotting of 10 paired specimens and cell-line profiling confirmed increased BPNT1 protein and mRNA in TNBC (Fig. 1K–M and Supplementary Fig. S1B). These results identify BPNT1 as an overexpressed, prognostically relevant TNBC biomarker.

To explore the potential mechanisms underlying BPNT1 overexpression in TNBC, we first assessed copy number amplification status of BPNT1 in breast cancer using The Cancer Genome Atlas Breast Cancer (TCGA-BRCA) dataset, and found that the amplification frequency of BPNT1 gene copy number was 5.8% in TNBC (Supplementary Fig. S2). Thus, we can not exclude the possibility that copy number amplification contributes to an upregulation of BPNT1 mRNA levels in TNBC. In addition, we also tried to identify the potential transcriptional regulators for BPNT1 using hTFtarget [31] and identified 16 candidate transcriptional regulators of BPNT1. Correlation analysis in FUSCC-TNBC data [17] found significant positive associations only for CEBPB (*r* = 0.245, *p* < 0.0001) and E2F1 (*r* = 0.301, *p* < 0.0001), with E2F1 showing the strongest link (Supplementary Fig. S3A). Consistently, we also demonstrated that BPNT1 promoter contains high-probability E2F1-binding motifs using JASPAR (https://jaspar.elixir.no/) database [32] (Supplementary Fig. S3B).

Taken together, these results suggest that copy number amplification and E2F1-mediated transcriptional regulation could be involved in BPNT1 upregulation in TNBC.

### BPNT1 promotes TNBC cell growth both in vitro and in vivo

**Fig. 2:**
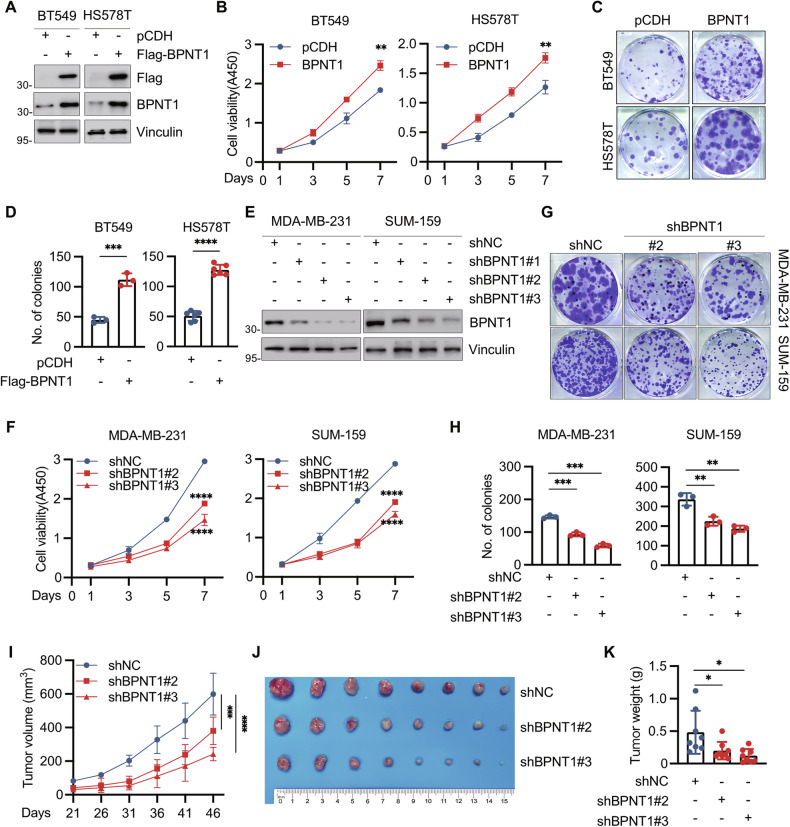
BPNT1 promotes TNBC cell proliferation and colony formation in vitro and xenograft tumor growth in vivo. **A** Immunoblotting analysis of the expression status of BPNT1 in BT549 and HS578T cells stably expressing empty vector pCDH and Flag-BPNT1. BT549 and HS578T cells stably expressing pCDH and Flag-BPNT1 were subjected to CCK-8 (**B**) and colony formation assays (**C**, **D**). Representative images of the survival colonies (**C**) and corresponding quantitative results (**D**) are shown. **E** Verification of the efficiency of shRNA-mediated knockdown of BPNT1 in MDA-MB-231 and SUM-159 cells stably expressing empty vector shNC and shBPNT1 (#1, #2, and #3) by immunoblotting. MDA-MB-231 and SUM-159 cells stably expressing shNC and shBPNT1 (#2 and #3) were subjected to CCK-8 (**F**) and colony formation assays (**G**, **H**). Representative images of the survival colonies (**G**) and corresponding quantitative results (**H**) are shown. **I**–**K** SUM-159 cells stably expressing shNC and shBPNT1 (#2 and #3) were inoculated into the mammary fat pad of 6-week-old BALB/c female nude mice (*n* = 8). After 46 days post-injection, mice were sacrificed, and xenograft tumors were removed. Tumor volume (**I**), representative tumor images (**J**), and tumor weight (**K**) are shown.

Given the differential basal expression of BPNT1 across TNBC cell lines, we overexpressed Flag-BPNT1 in low-expressing BT549 and HS578T cells (Fig. 2A), which enhanced proliferation and colony formation (Fig. 2B–D). Conversely, shRNA-mediated BPNT1 knockdown in MDA-MB-231 and SUM-159 decreased viability and colony formation (Fig. 2E–H). To verify the tumourigenic effect of BPNT1 in mice, SUM-159 cells expressing empty vector shNC or shBPNT1 (#2 and #3) were orthotopically implanted into mammary fat pads of BALB/c nude mice (6–8 weeks, *n* = 8). Consistent with in vitro observations, BPNT1 knockdown suppressed tumor growth in vivo (volume, weight; Fig. 2I-K). Together, these results suggest that BPNT1 promotes TNBC cell growth both in vitro and in vivo.

### BPNT1 promotes the migratory, invasive, and metastatic potential of TNBC cells both in vitro and in vivo

**Fig. 3:**
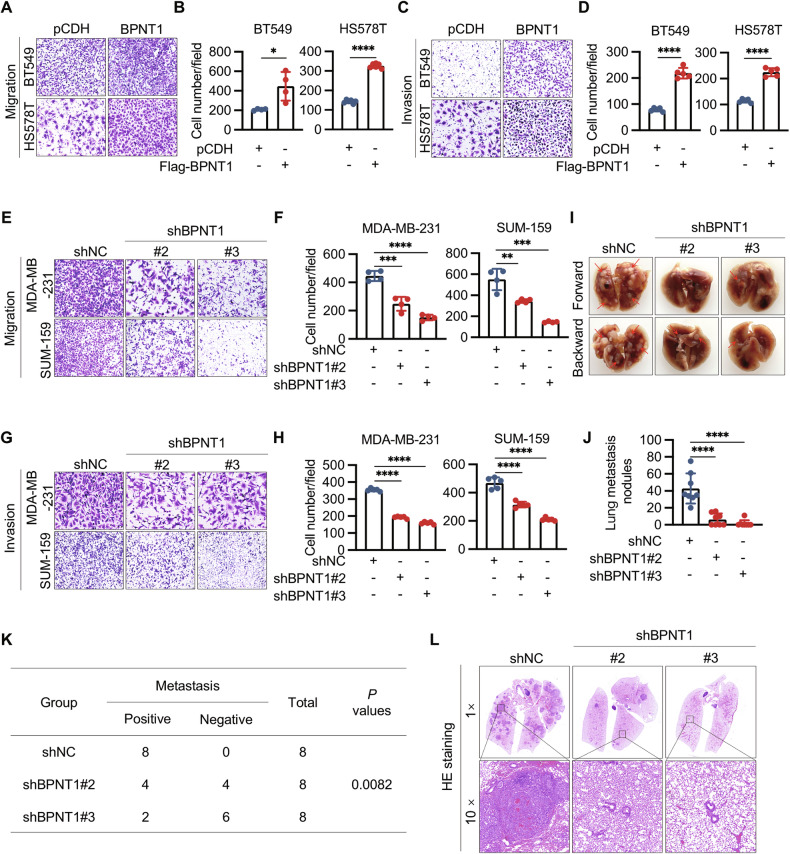
BPNT1 promotes TNBC cell migration and invasion in vitro and lung metastatic potential in vivo. BT549 and HS578T cells stably expressing pCDH and Flag-BPNT1 were subjected to transwell migration (**A**, **B**) and invasion (**C**, **D**) assays. Representative images of migrated and invaded cells are shown in **A**, **C** and the corresponding quantitative results are shown in **B**, **D**, respectively. MDA-MB-231 and SUM-159 cells stably expressing shNC and shBPNT1 (#2 and #3) were subjected to transwell migration (**E**, **F**) and invasion (**G**, **H**) assays. Representative images of migrated and invaded cells are shown in (**E**, **G**) and corresponding quantitative results are shown in (**F**, **H**), respectively. **I**–**L** SUM-159 cells stably expressing shNC and shBPNT1 (#2 and #3) were injected into the tail vein of 7-week-old BALB/c female nude mice (*n* = 8). After 6 weeks of injection, mice were sacrificed, and lungs were removed. Representative images of lung metastasis (**I**), corresponding quantitative results of metastatic lung nodules (**J**), the incidence of lung metastasis (**K**), and representative images of HE (hematoxylin-eosin) staining of lung tissues (**L**) are shown, respectively.

Next, we investigated the potential effects of BPNT1 on the migratory and invasive abilities of TNBC cells. BPNT1 overexpression enhanced transwell migration and invasion in BT549 and HS578T, while BPNT1 depletion impaired these phenotypes in MDA-MB-231 and SUM-159 (Fig. 3A–H). In tail-vein metastasis assays, BPNT1 knockdown reduced lung metastatic burden (nodule count, incidence; Fig. 3J–L; Supplementary Fig. S4A), indicating BPNT1 promotes motility and metastasis.

To test whether BPNT1 enzymatic activity is required, we generated a catalytic mutant (BPNT1 D51A) by mutating a key residue within its active site [13]. Immunoblotting confirmed that the D51A mutation did not affect the expression level of the BPNT1 protein (Supplementary Fig. S4B). Both WT and D51A mutant restored proliferation, colony formation, migration, and invasion in BPNT1-depleted cells (Supplementary Fig. S4C–H), indicating a non-catalytic mechanism.

### BPNT1 interacts with LIMA1 and induces its proteasomal degradation in TNBC cells

**Fig. 4:**
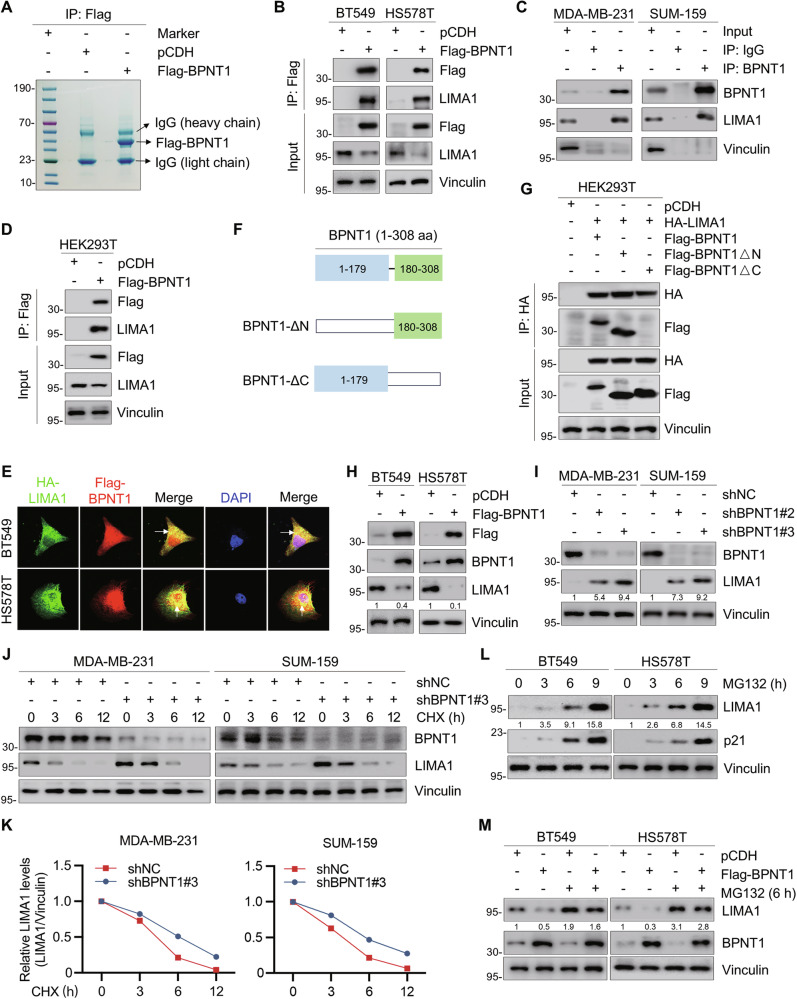
BPNT1 interacts with LIMA1 and induces its degradation in TNBC cells. (**A**) HEK293T cells stably expressing empty vector pCDH and Flag-BPNT1 were subjected to IP assays using anti-Flag affinity gel beads. (**B**) BT549 and HS578T cells stably expressing pCDH and Flag-BPNT1 were subjected to IP and immunoblotting assays with the indicated antibodies. (**C**) IP assays using control IgG or an anti-BPNT1 antibody were performed to detect the endogenous interaction between BPNT1 and LIMA1 in MDA-MB-231 and SUM-159 cells. (**D**) HEK293T cells transfected with the pCDH and Flag-BPNT1 were subjected to IP assays and followed by immunoblotting with the indicated antibodies. (**E**) Immunofluorescent staining showing the partial colocalization of LIMA1 and BPNT1 (as indicated by white arrows) in BT549 and HS578T cells stably expressing HA-LIMA1 (green) and Flag-BPNT1 (red). The nucleus was counterstained with DAPI (blue). (**F**) Schematic presentation of domains of wild-type BPNT1, C-terminal (BPNT1-ΔN, aa 180-308) and N-terminal (BPNT1-ΔC, aa 1-179) truncation mutants of BPNT1. (**G**) HEK293T cells transfected with the indicated expression vectors were subjected to IP assays and followed by immunoblotting with the indicated antibodies. Detection of the expression levels of LIMA1 in BT549 and HS578T cells stably expressing pCDH and Flag-BPNT1 (**H**) and in MDA-MB-231 and SUM-159 cells stably expressing shNC and shBPNT1 (#2 and #3) (**I**) by immunoblotting assays with the indicated antibodies. **J**, **K** MDA-MB-231 and SUM-159 cells expressing shNC and shBPNT1 (#3) were treated with DMSO or 100 mg/mL CHX for the indicated times and subjected to immunoblotting assays (**J**). Quantitative results of relative LIMA1 protein levels (LIMA1/Vinculin) analyzed by ImageJ are shown in (**K**). **L** Immunoblotting assays showing the expression levels of LIMA1 in TNBC cells treated with DMSO or 10 μM MG-132. P21 was used as a positive control. **M** Immunoblotting assays examining the expression levels of LIMA1 in TNBC cells expressing pCDH and Flag-BPNT1 treated with DMSO or 10 μM MG-132 for 6 h.

To explore BPNT1’s mechanism of action which facilitates TNBC progression, we performed IP assay coupled with LC-MS/MS proteomic profiling in HEK293T cells stably expressing Flag-BPNT1 or empty vector pCDH (Fig. 4A and Supplementary Fig. S5A). Applying a stringent threshold (unique peptides ≥2), 59 high-confidence BPNT1-interacting proteins were identified, with the top 10 candidates cataloged in Supplementary Fig. S5B. Gene ontology-molecular function (GO-MF) analysis showed enrichment for cadherin binding, unfolded protein binding, and actin filament binding (Supplementary Fig. S5C).

Based on the above results, we selected LIMA1, the top-ranked BPNT1-interacting protein, for further verification. Reciprocal co-IP confirmed endogenous and exogenous BPNT1–LIMA1 interactions across cell lines (Fig. 4B–D), and immunofluorescence showed partial co-localization in BT549 and HS578T (Fig. 4E). Together, these results suggest that LIMA1 indeed interacts with BPNT1.

To identify the functional domain of BPNT1 required for LIMA1 interaction, we constructed N-terminal (BPNT1-ΔC, aa 1-179) and C-terminal (BPNT1-ΔN, aa 180-308) truncation mutants of BPNT1 (Fig. 4F). Subsequent co-immunoprecipitation assays demonstrated that while both wild-type BPNT1 and the C-terminal truncation mutant (BPNT1-ΔN) robustly interacted with LIMA1, the N-terminal truncation mutant (BPNT1-ΔC) completely lost its binding capacity with LIMA1 (Fig. 4G). Collectively, these data establish that the BPNT1-LIMA1 interaction is mediated specifically by the C-terminal domain (aa 180-308) of BPNT1.

Next, we sought to explore whether there is a regulatory relationship between BPNT1 and LIMA1. In public datasets (TCGA, METABRIC, CPTAC), LIMA1 protein was decreased in TNBC compared with adjacent tissues (Supplementary Fig. S5D–F). In 10 paired clinical samples, BPNT1 inversely correlated with LIMA1 (Supplementary Fig. S5G, H). BPNT1 overexpression reduced LIMA1 protein dose-dependently, whereas BPNT1 knockdown increased LIMA1 (Fig. 4H, I); LIMA1 mRNA remained unchanged (Supplementary Fig. S5I), indicating post-transcriptional regulation. Protein synthesis inhibitor cycloheximide (CHX) chase showed prolonged LIMA1 half-life after BPNT1 depletion (Fig. 4J, K), consistent with BPNT1 affecting LIMA1 stability.

To determine if the observed LIMA1 degradation depended on the BPNT1-LIMA1 interaction, we exogenously expressed wild-type BPNT1 and two truncation mutants (BPNT1-ΔN: aa 180-308; BPNT1-ΔC: aa 1-179) in HS578T and BT549 cells. Immunoblotting assays revealed that only the interaction-deficient mutant BPNT1-ΔC failed to degrade LIMA1 protein, whereas both wild-type BPNT1 and the interaction-competent mutant BPNT1-ΔN effectively downregulated LIMA1 levels (Supplementary Fig. S5J).

Prior evidence indicates LIMA1 undergoes ubiquitin-proteasome system (UPS) mediated degradation in specific contexts [24–26]. We treated BT549 and HS578T with proteasome inhibitor MG-132 and found that LIMA1 and p21 [33] both accumulated in a time-dependent manner (Fig. 4L). In contrast, Baf-A1 (autophagy inhibitor) increased p62, a known autophagy-lysosome system substrate [34], but did not alter LIMA1 (Supplementary Fig. S5K). MG-132 restored BPNT1-mediated LIMA1 downregulation (Fig. 4M), supporting UPS-dependent destabilization by BPNT1.

### BPNT1 recruits E3 ubiquitin-protein ligase STUB1 to catalyze LIMA1 ubiquitination and subsequent proteasomal degradation

**Fig. 5:**
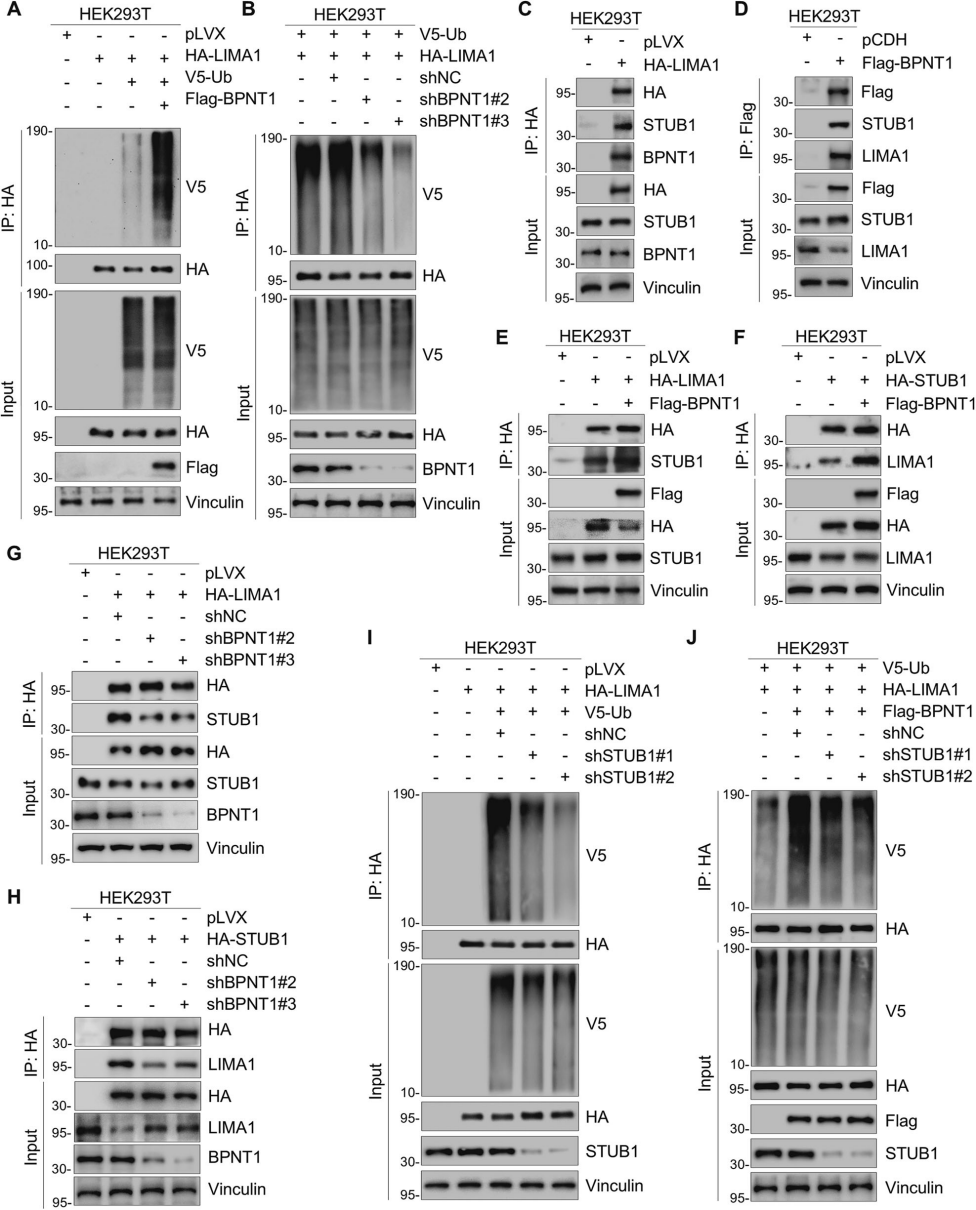
BPNT1 recruits E3 ubiquitin-protein ligase STUB1 to promote ubiquitination-dependent proteasomal degradation of LIMA1. Immunoblotting assays assessing LIMA1 ubiquitination levels in HEK293T cell lines transfected with Flag-BPNT1 (**A**) or shBPNT1 (#2 and #3) (**B**). **C**, **D** IP assays of the interaction between BPNT1, LIMA1, and STUB1 in HEK293T cells. HEK293T cells transfected with pLVX and HA-LIMA1 (**C**), and pCDH and Flag-BPNT1 (**D**), respectively, were subjected to IP assays, followed by immunoblotting with the indicated antibodies. Immunoblotting assays investigating the expression levels of STUB1 (**E**, **G**) and LIMA1 (**F**, **H**) in HEK293T cells transfected with the indicated plasmids. **I**, **J** HEK293T cells were co-transfected with HA-LIMA1, V5-ubiquitin (Ub), Flag-BPNT1 alone or in combination, and then infected with or without shNC and shSTUB1 (#1 and #2) lentivirus, followed by incubation with 10 μM MG-132 for 6 h. IP analysis using HA-tagged beads was performed to detect the ubiquitination levels of LIMA1.

Ubiquitination assays showed increased LIMA1 ubiquitination on BPNT1 overexpression and decreased ubiquitination upon BPNT1 knockdown (Fig. 5A, B). As BPNT1 is not a putative E3 ubiquitin ligase, we hypothesized its role as a molecular adaptor that scaffolds E3 ubiquitin ligase recruitment for LIMA1 ubiquitination. LC-MS/MS data identified STUB1 (also known as CHIP) as a candidate BPNT1 partner [35–38] (Fig. 4C).

To validate STUB1’s role in BPNT1-mediated LIMA1 regulation, we first confirmed the formation of a tripartite complex through reciprocal co-IP assays, which demonstrated endogenous interactions among LIMA1, STUB1 and BPNT1 (Fig. 5C, D and Supplementary Fig. S6A). STUB1 knockdown decreased, while overexpression increased LIMA1 protein levels without affecting mRNA (Supplementary Fig. S6B–E). Critically, BPNT1 dynamically modulates the LIMA1-STUB1 interaction, as evidenced by IP assays showing that BPNT1 overexpression disrupted (Fig. 5E, F), whereas BPNT1 knockdown enhanced (Fig. 5G, H), physical association between LIMA1 and STUB1. Definitive ubiquitination analyses further established that STUB1 depletion markedly attenuated BPNT1-induced polyubiquitination of LIMA1 (Fig. 5I, J). Collectively, these data demonstrate that BPNT1 scaffolds STUB1 recruitment to catalyze ubiquitin-dependent proteasomal degradation of LIMA1, thereby depleting this tumor suppressor in TNBC.

To establish the functional dependency of BPNT1’s oncogenic effects on the STUB1-mediated degradation of LIMA1, we performed rescue experiments in STUB1-depleted MDA-MB-231 and SUM-159 cells. First, immunoblotting assays confirmed that BPNT1 overexpression failed to reduce LIMA1 protein levels in the context of STUB1 knockdown (Supplementary Fig. S6F), demonstrating that STUB1 is essential for BPNT1-induced LIMA1 downregulation. Strikingly, while STUB1 knockdown itself significantly reduced cell growth, colony formation capacity, and migratory/invasive abilities (consistent with its role in promoting malignancy via LIMA1 degradation), reintroduction of BPNT1 into these STUB1-depleted cells failed to restore cell proliferation, colony formation, or motile and invasive capacities (Supplementary Fig. S6G–L). Collectively, these genetic rescue experiments demonstrated that the tumor-promoting functions of BPNT1 in TNBC are largely dependent on the STUB1-mediated ubiquitination and degradation of LIMA1.

### BPNT1 augments TNBC progression partially through regulating LIMA1-mediated EMT

**Fig. 6:**
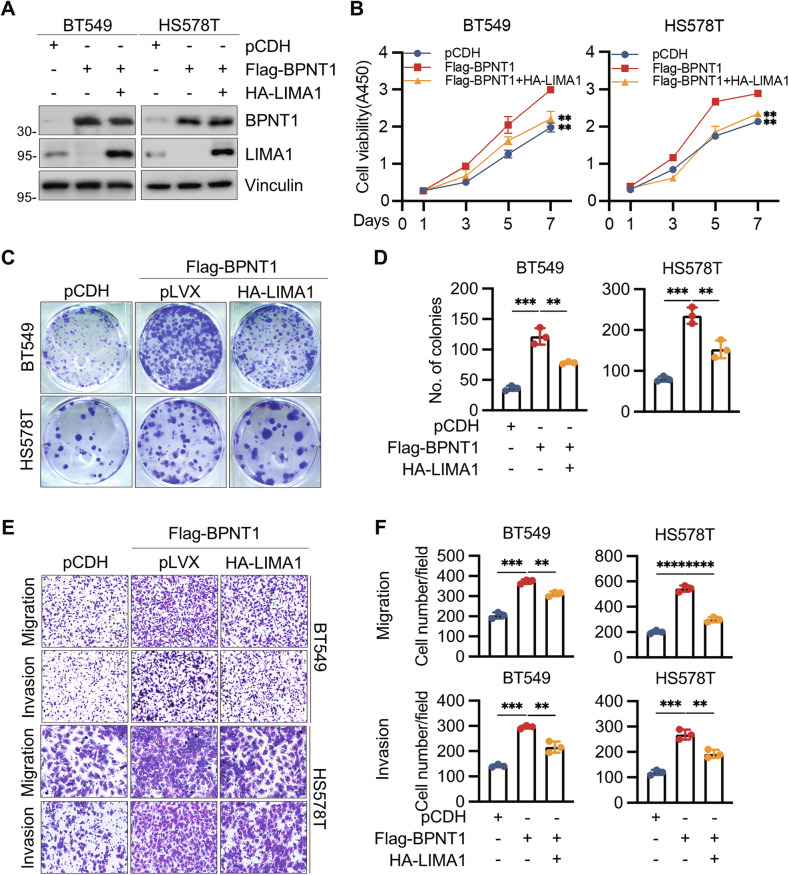
Reintroduction of LIMA1 impairs BPNT1-mediated TNBC cell proliferation, migration, and invasion in vitro. **A** BT549 and HS578T cells stably expressing pCDH or Flag-BPNT1 were transfected with or without HA-LIMA1. After 48 h of transfection, cells were subjected to immunoblotting analysis with the indicated antibodies. BT549 and HS578T cells stably expressing pCDH or Flag-BPNT1 alone or in combination with HA-LIMA1 were subjected to CCK-8 (**B**) and colony formation assays (**C**, **D**). Representative images of the survival colonies (**C**) and corresponding quantitative results (**D**) are shown, respectively. **E**, **F** BT549 and HS578T cells stably expressing pCDH or Flag-BPNT1 alone or in combination with HA-LIMA1 were subjected to transwell migration and invasion assays. Representative images of migrated and invaded cells are shown in (**E**) and corresponding quantitative results are shown in (**F**).

To elucidate the functional dependency of BPNT1’s oncogenicity on LIMA1 suppression, we re-expressed LIMA1 in BPNT1-overexpressing BT549 and HS578T cells (Fig. 6A) and performed function rescue assays. Re-expression of LIMA1 partially rescued the enhanced proliferation, colony formation, migration, and invasion induced by BPNT1 overexpression (Fig. 6B–F). Notably, forced LIMA1 expression substantially reversed BPNT1-driven migratory and invasive potentiation (Fig. 6E, F), indicating LIMA1 restoration can counteract BPNT1’s pro-metastatic programming.

**Fig. 7:**
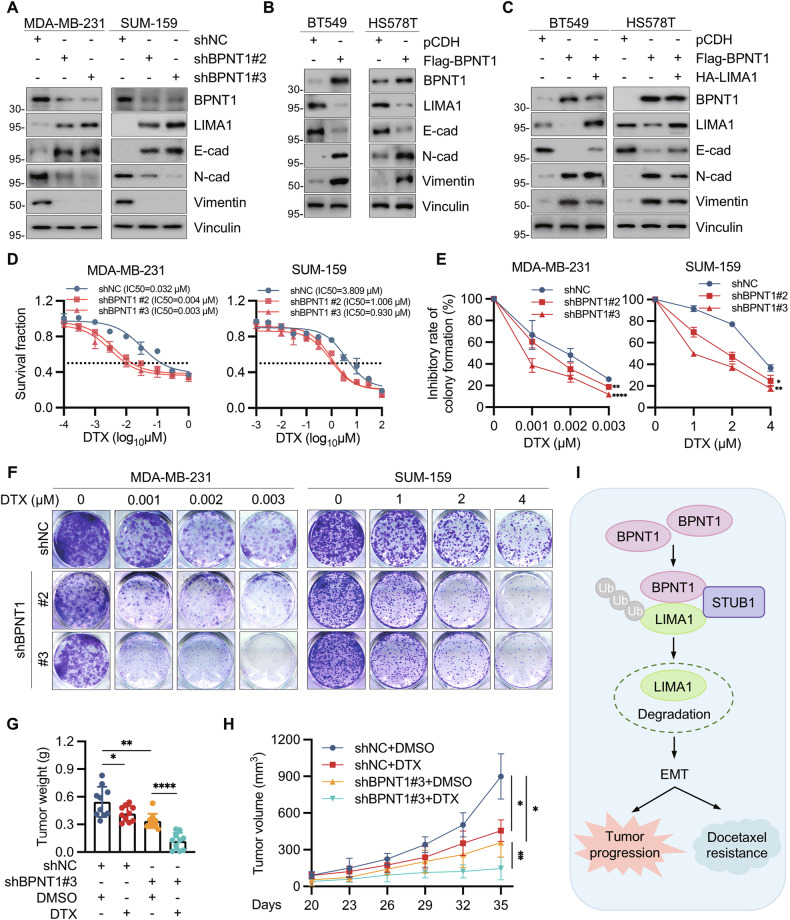
BPNT1 accelerates TNBC progression via EMT and promotes resistance of TNBC cells to docetaxel. The expression levels of EMT-related makers were detected by immunoblotting in cells with overexpression (**A**) and knockdown (**B**) of BPNT1, respectively. **C** BT549 and HS578T cells stably expressing pCDH or Flag-BPNT1 alone or in combination with HA-LIMA1 were subjected to immunoblotting analysis with the indicated antibodies. **D** MDA-MB-231 and SUM-159 cells stably expressing empty vector shNC or shBPNT1 (#2 and #3) were treated with or without increasing doses of docetaxel (DTX). Cell viability was determined using CCK-8 assays. The IC50 values are shown. **E**, **F** MDA-MB-231 and SUM-159 cells stably expressing empty vector shNC or shBPNT1 (#2 and #3) were treated without or with the indicated concentrations of DTX and subjected to colony formation assays. Representative images of survival colonies and corresponding quantitative results are shown in (**E**, **F**), respectively. **G**, **H** SUM-159 cells stably expressing shNC or shBPNT1 (#3) were inoculated into mammary fat pad of 6-week-old BALB/c female nude mice (*n* = 20). After 20 days of injection, mice were randomly divided into two groups (*n* = 10), and were administered with or without DTX (20 mg/kg, dissolved in DMSO) via intraperitoneal injection every 2 days for a total of 6 doses. Tumor weight (**G**) and tumor volume (**H**) are shown. **I** The proposed working model. Accumulating BPNT1 in TNBC recruits the E3 ubiquitin ligase STUB1 to mediate ubiquitination and proteasomal degradation of LIMA1, thus promoting EMT progression to drive TNBC progression and resistance to DTX.

Given the established role of LIMA1 deficiency in promoting EMT through adhesion complex disassembly [24, 39], we systematically evaluated EMT marker dynamics (E-cadherin, N-cadherin, vimentin) [40, 41] in BPNT1-modulated TNBC models. Immunoblotting revealed that BPNT1 knockdown in MDA-MB-231 and SUM-159 cells induced epithelial restitution, characterized by E-cadherin (an epithelial marker) upregulation and concomitant N-cadherin/vimentin (mesenchymal markers) downregulation (Fig. 7A); conversely, BPNT1 overexpression in BT549 and HS578T cells drove mesenchymal transition with inverted marker patterns (Fig. 7B). Critically, LIMA1 reconstitution mitigated these EMT changes (Fig. 7C), indicating BPNT1 fosters progression partly via LIMA1-dependent EMT.

### BPNT1 promotes resistance of TNBC cells to DTX both in vitro and in vivo

Given LIMA1’s role in cytoskeletal dynamics and microtubule-targeting agent response [20, 42–44], we tested BPNT1’s effect on DTX sensitivity [45]. GDSC analysis linked the BPNT1–LIMA1 axis to DTX response (Supplementary Fig. S7A). BPNT1 depletion increased DTX sensitivity in vitro (CCK-8 and colony assays; Fig. 7D–F) and enhanced DTX efficacy in xenografts (Fig. 7G, H; Supplementary Fig. S7B, C).

Collectively, these findings delineate a novel oncogenic axis wherein BPNT1 scaffolds STUB1-mediated ubiquitination and degradation of LIMA1, driving EMT-facilitated tumor progression and DTX chemoresistance (Fig. 7I).

## Discussion

While previous research on BPNT1 focused on sulfur metabolism [11, 13, 14, 46], its oncogenic role in TNBC remained undefined [14–16]. Our data identify BPNT1 as a TNBC-associated oncoprotein that recruits STUB1 to drive ubiquitin-dependent degradation of the tumor suppressor LIMA1, thereby promoting malignancy and docetaxel resistance; BPNT1 expression thus carries prognostic and therapeutic implications.

LIMA1 was identified as one of the binding targets of BPNT1 by LC-MS/MS. LIMA1 is frequently downregulated and suppresses tumorigenesis in many cancers by activating several signaling axes, such as Wnt/β-catenin, PI3K/AKT, and FAK/Src [47–50]. However, LIMA1 is upregulated in head and neck squamous cell carcinoma, which is triggered by DNA demethylation of its promoter region [51, 52]. LIMA1 has been documented as a metastasis suppressor in breast cancer [25, 50], a finding corroborated by our multi-omics analyses showing significant LIMA1 downregulation in TNBC versus paracancerous tissues across FUSCC transcriptomic and proteomic datasets [17, 18]. These results were further verified in clinical tissue specimens and public CPTAC datasets (Supplementary Fig. S5B–D). In addition, further functional validations demonstrated that reintroduction of LIMA1 suppressed malignant biological behavior of TNBC cells and EMT process (Figs. 6 and 7C). In this context, the prognostic predictive effects and therapeutic potential of LIMA1 in breast cancer warrant further exploration.

Mechanistically, our data show that BPNT1 downregulates LIMA1 at the protein level rather than at mRNA level. In fact, accumulating evidence has elucidated the mechanisms of numerous transcriptional and post-translational pathways involved in the regulation of LIMA1 expression [26, 53–55]. Pan-cancer analyses have indicated that p53 transactivates LIMA1 gene promoter to exert tumor-suppressive effects [56]. Another study suggests that cancer associated fibroblasts (CAF) deploy miR-20a-5p-loaded exosomes to silence LIMA1 in hepatocellular carcinoma [57]. Meanwhile, ubiquitination degradation is another important mechanism for post-transcriptional regulation of LIMA1 [24]. Previous studies have reported that deubiquitinase USP44 participates in the degradation of LIMA1 in cholangiocarcinoma [26]. In this study, we demonstrated a novel regulatory mechanism for LIMA1 degradation by BPNT1 (Figs. 4 and 5).

Considering that BPNT1 is not an E3 ubiquitin-protein ligase or deubiquitinase, we further probed its potential partner in BPNT1-mediated LIMA1 ubiquitination. STUB1 exhibits functional versatility across oncogenic processes—spanning tumor initiation, advancement, metastatic dissemination, chemoresistance, and clinical outcome [58–64]. This E3 ligase displays paradoxical duality, functioning context-dependently as an oncogene or tumor suppressor contingent upon malignancy type and substrate specificity [65, 66]. Our findings position BPNT1 as a novel molecular scaffold that redirects STUB1’s catalytic activity toward LIMA1 degradation, resolving its tumor-promoting role in TNBC (Figs. 4 and 5). However, we cannot entirely exclude the possibility that BPNT1 affects LIMA1 levels via other pathways.

Cytotoxic chemotherapeutics such as taxanes and anthracyclines have been shown to be effective in patients with TNBC [2]. EMT is a key driver of resistance to microtubule-targeting agents like DTX, a mainstay TNBC chemotherapy [67, 68]. As BPNT1 downregulates LIMA1 to promote EMT, we investigated its role in DTX resistance. BPNT1 knockdown sensitized TNBC cells to DTX in vitro and in vivo (Fig. 7), positioning BPNT1 as a biomarker and target to overcome DTX resistance.

Collectively, we delineate a pathogenic signaling cascade wherein BPNT1 scaffolds STUB1-mediated ubiquitination and degradation of LIMA1, activating EMT to drive TNBC progression while concurrently establishing DTX chemoresistance. These results may provide evidence for exploring novel therapeutic strategies targeting BPNT1 in patients with TNBC.

## Supplementary information

Supplementary information

Ling et al. Original western blots 10-28-2025

## Data Availability

Study data incorporates FUSCC-TNBC cohort analyses alongside public datasets (TCGA, CPTAC, METABRIC) accessible via www.cbioportal.org. The manuscript and Supplementary materials collectively provide full data support for all conclusions.
