# Supplementary material for: Bisphosphate nucleotidase 1 promotes progression and docetaxel resistance in triple-negative breast cancer via STUB1-mediated destabilization of LIMA1
Source: Cell Death Dis. 2026 Jan 15;17(1):40. doi: 10.1038/s41419-025-08245-0 (PMC12808305; doi:10.1038/s41419-025-08245-0)
Supplement: Supplementary file 1 — Supplementary information [file 41419_2025_8245_MOESM1_ESM.docx]

**Supplementary information for**

Ling et al. Bisphosphate nucleotidase 1 promotes progression and docetaxel resistance in triple-negative breast cancer via STUB1-mediated destabilization of LIMA1

**Supplementary information contains:**

Supplementary Figures 1-7

Supplementary Tables 1-5

**Supplementary Figures and Figure legends**

**
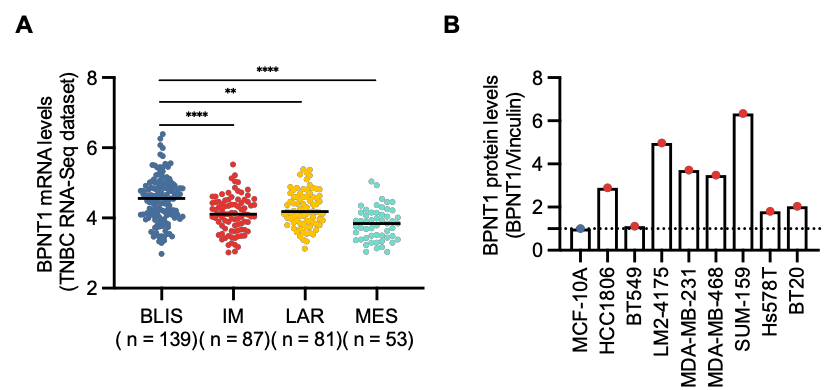
**

**Figure S1. BPNT1 is overexpressed in TNBC tissues and cell lines**

(A) Analysis of BPNT1 mRNA levels in different TNBC subtypes using FUSCC TNBC RNA-Seq dataset.

(B) Quantitative results of relative BPNT1 protein expression levels in human mammary epithelial cell line MCF10A and eight representative human TNBC cell lines. quantification of relative BPNT1 protein levels (BPNT1/Vinculin) was conducted using ImageJ software.


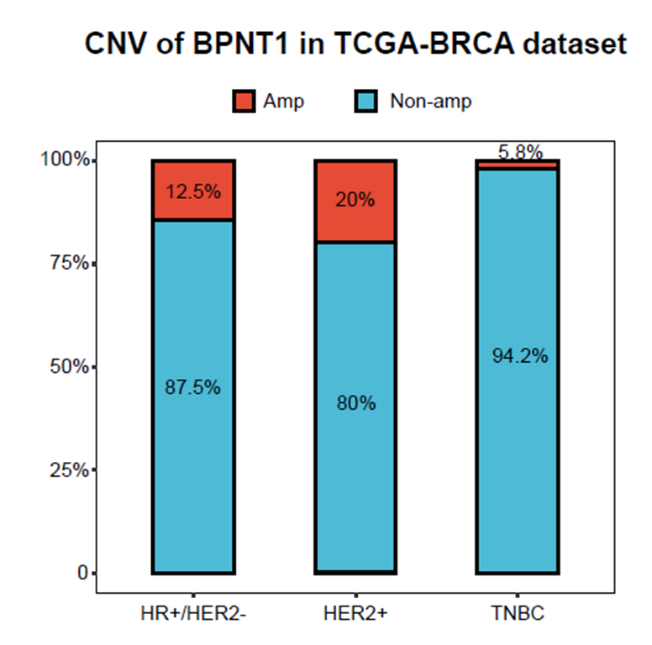


**Figure S2. Analysis of copy number amplification status of BPNT1 in breast cancer using The Cancer Genome Atlas Breast Cancer (TCGA-BRCA) dataset**


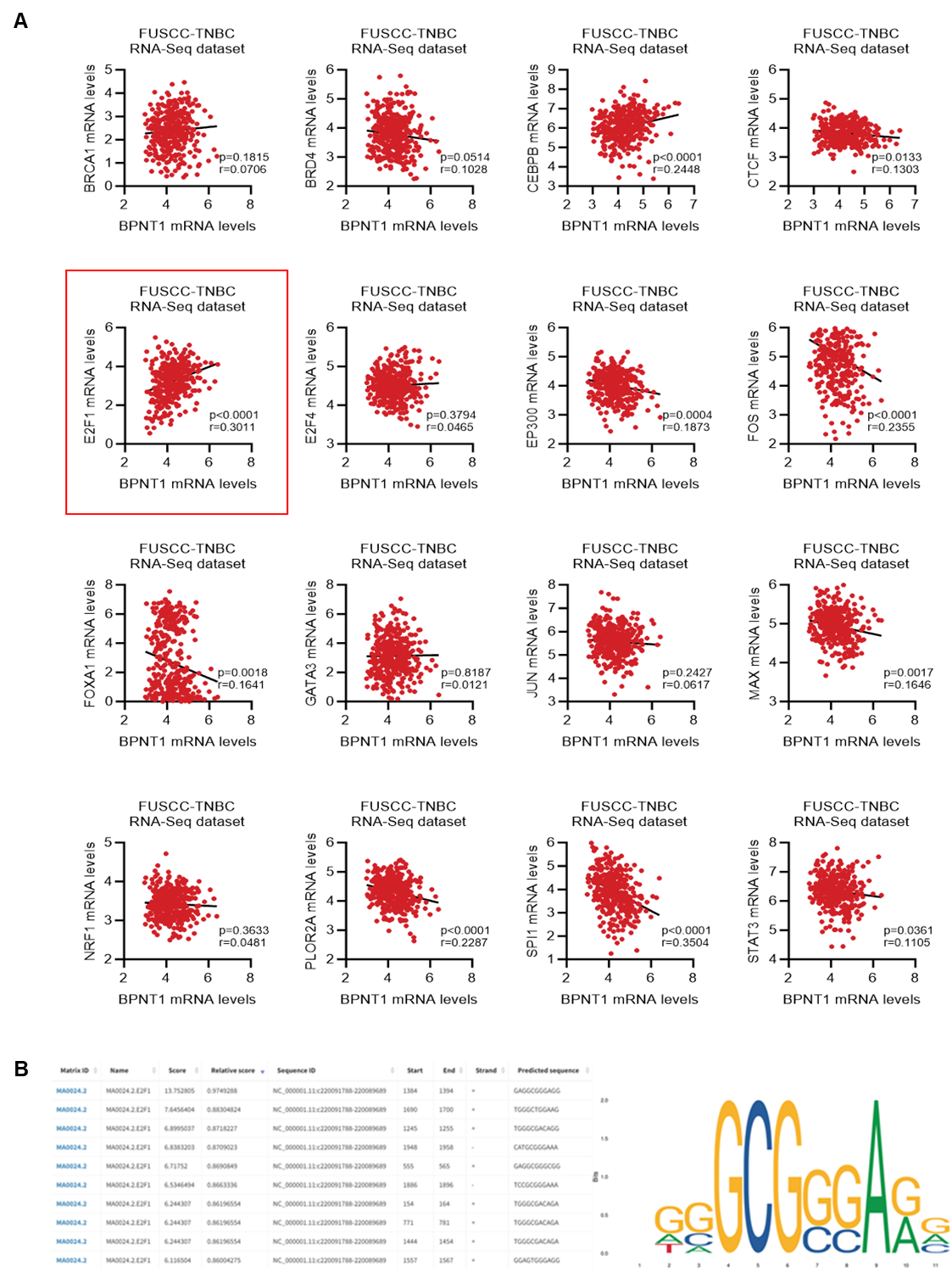


**Figure S3. Identification of the potential transcription regulators of BPNT1 in TNBC**

(A) Analysis of the potential transcriptional regulators for BPNT1 using hTFtarget, a comprehensive database for regulations of human transcription factors and their targets[1]. The results revealed 16 potential transcriptional regulators of BPNT1 in breast tissues. Further correlation analysis in the FUSCC-TNBC RNA-seq dataset [2] demonstrated that only transcription factors CEBPB (Pearson's r =0.245, *p*< 0.0001) and E2F1 (Pearson's r=0.301, *p*< 0.0001) showed significantly positive correlations with BPNT1 expression levels, with E2F1 exhibiting the strongest association.

(B) Identification of high-probability E2F1-binding motifs in BPNT1 promote using JASPAR (<https://jaspar.elixir.no/>) database [3].

**
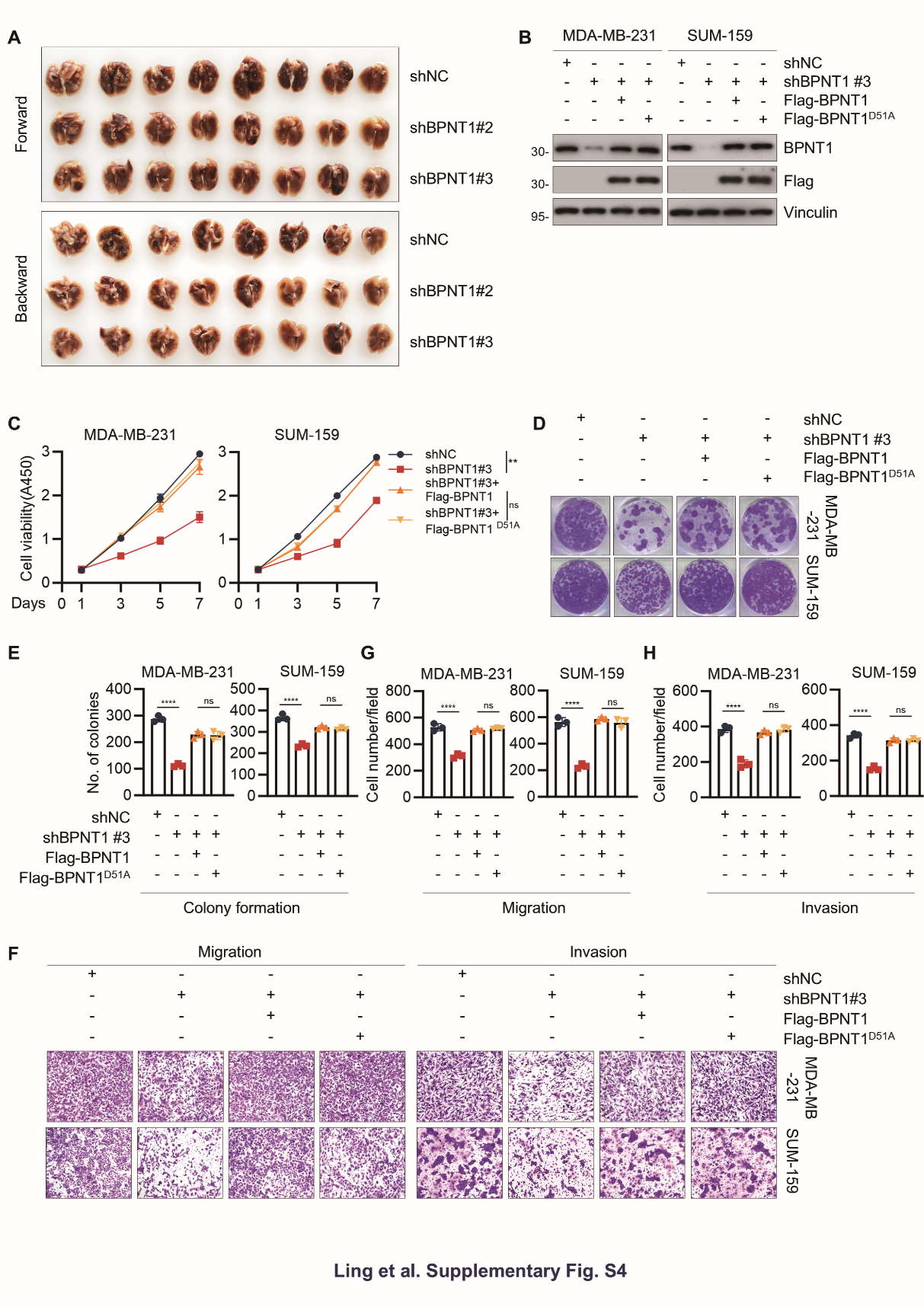
**

**Figure S4. BPNT1 promotes TNBC cell growth, migration, and invasion independent of its intrinsic enzymatic activity**

(A) Images of removed lung tissues related to **Figure 3I-L** are shown.

(B) Immunoblotting analysis of the expression status of BPNT1 in MDA-MB-231 and SUM-159 cells stably expressing shNC or shBPNT1#3 alone or in combination with Flag-BPNT1 and Flag-BPNT1^D51A^.

(C-E) MDA-MB-231 and SUM-159 cells stably expressing shNC or shBPNT1#3 alone or in combination with Flag-BPNT1 and Flag-BPNT1^D51A^ were subjected to CCK-8 (C) and colony formation assays (D and E). Representative images of the survival colonies (D) and corresponding quantitative results (E) are shown, respectively.

(F-H) MDA-MB-231 and SUM-159 cells stably expressing shNC or shBPNT1#3 alone or in combination with Flag-BPNT1 and Flag-BPNT1^D51A^ were subjected to transwell migration and invasion assays. Representative images of migrated and invaded cells are shown in F, and corresponding quantitative results are shown in G (migration assay) and H (invasion assay).


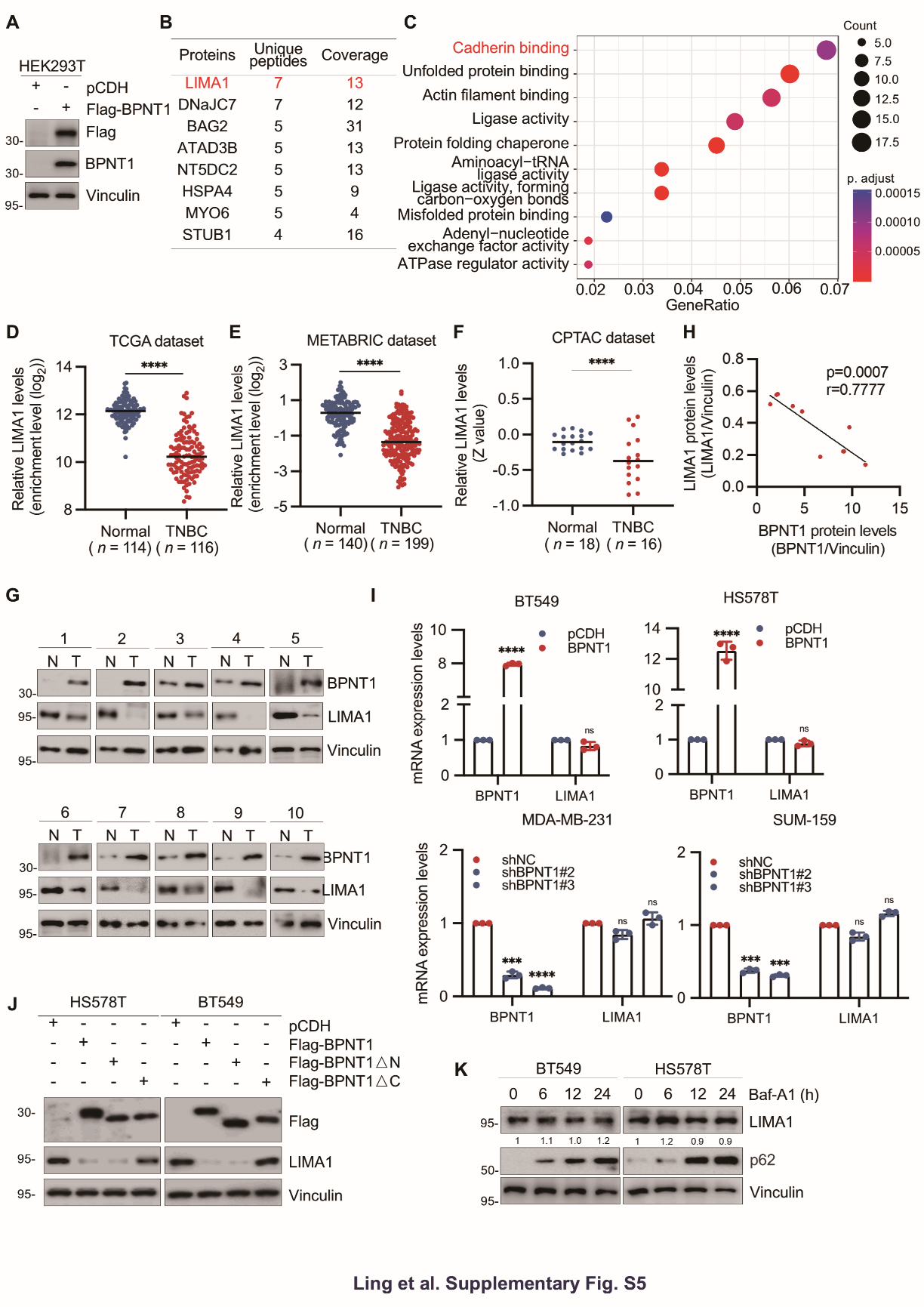


**Figure S5. BPNT1 regulates LIMA1 at post transcriptional level**

(A) Validation of HEK293T cells stably expressing empty vector pCDH and Flag-BPNT1 by immunoblotting.

(B) The top 10 BPNT1-interacting proteins according to the number of identified unique peptides.

(C) Gene Ontology-molecular function (GO-MF) analysis of BPNT1-interacting proteins.

(D) Analysis of LIMA1 mRNA levels in 114 normal tissues and 116 TNBC tissues in the TCGA dataset.

(E) Analysis of LIMA1 mRNA levels in 140 normal tissues and 199 TNBC tissues in the METABRIC dataset.

(F) Analysis of LIMA1 protein levels in 18 normal tissues and 16 TNBC tissues in the CPTAC dataset.

(G) Immunoblotting analysis of BPNT1 and LIMA1 protein expression levels in 10 pairs of TNBC tissues and matched normal breast tissues.

(H) Spearman analysis of the correlation of the protein levels between BPNT1 and LIMA1 in tissues (E) is shown.

(I) RT-qPCR analysis of the mRNA expression levels of LIMA1 in cells with overexpression or knockdown of BPNT1.

(J) Immunoblotting analysis of the expression status of LIMA1 in BT549 and HS578T cells stably expressing empty vector pCDH, Flag-BPNT1, Flag-BPNT1 ΔN and Flag-BPNT1 ΔC.

(K) Immunoblotting assays showing the expression levels of LIMA1 in TNBC cells treated with DMSO or 200 nM Baf-A1 for the indicated time. P62, a known substrate of the autophagy-lysosome system, was used as a positive control.

**
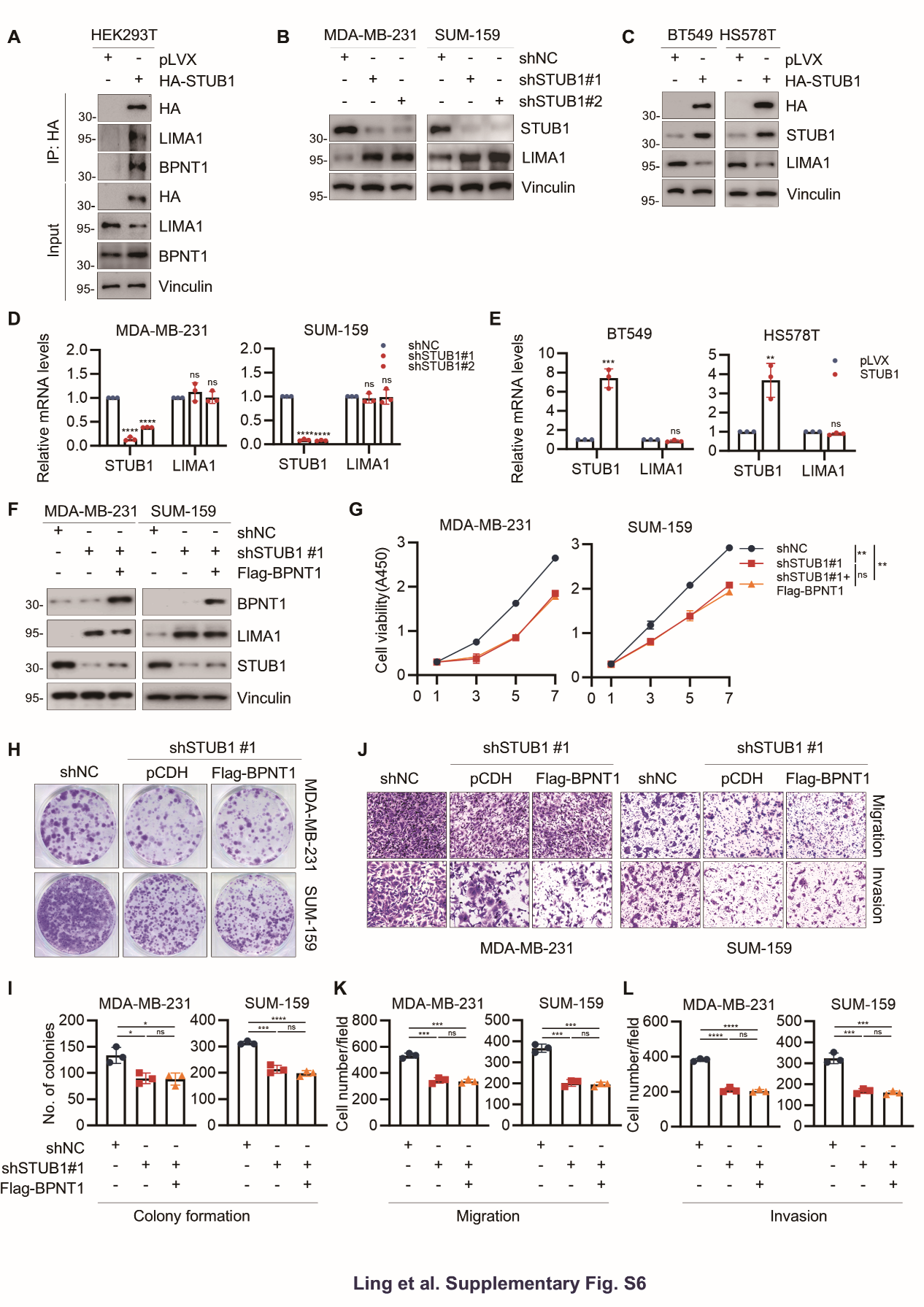
**

**Figure S6. STUB1 promote proteasomal degradation of LIMA1** **and is essential for BPNT1-induced oncogenic phenotypes in TNBC cells**

(A) HEK293T cells. HEK293T cells transfected with pLVX and HA-STUB1 were subjected to IP assays, followed immunoblotting with the indicated antibodies to detect the interaction between STUB1, BPNT1 and LIMA1.

(B-C) Immunoblotting analyses of the expression levels of LIMA1 in MDA-MB-231 and SUM-159 cells stably expressing shNC and shSTUB1 (#1 and #2) (B) and in BT549 and HS578T cells stably expressing pLVX and HA-STUB1 (C).

(D-E) RT-qPCR analysis of the mRNA expression levels of LIMA1 in cells with knockdown (D) or overexpression (E) of STUB1.

(F) Immunoblotting analysis of the expression status of LIMA1 in MDA-MB-231 and SUM-159 cells stably expressing shNC or shSTUB1#1 alone or in combination with Flag-BPNT1.

(G-I) MDA-MB-231 and SUM-159 cells stably expressing shNC or shSTUB1#1 alone or in combination with Flag-BPNT1 were subjected to CCK-8 (G) and colony formation assays (H and I). Representative images of the survival colonies (H) and corresponding quantitative results (I) are shown, respectively.

(J-L) MDA-MB-231 and SUM-159 cells stably expressing shNC or shSTUB1#1 alone or in combination with Flag-BPNT1 were subjected to transwell migration and invasion assays. Representative images of migrated and invaded cells are shown in J, and corresponding quantitative results are shown in K (migration assay) and L (invasion assay).

**
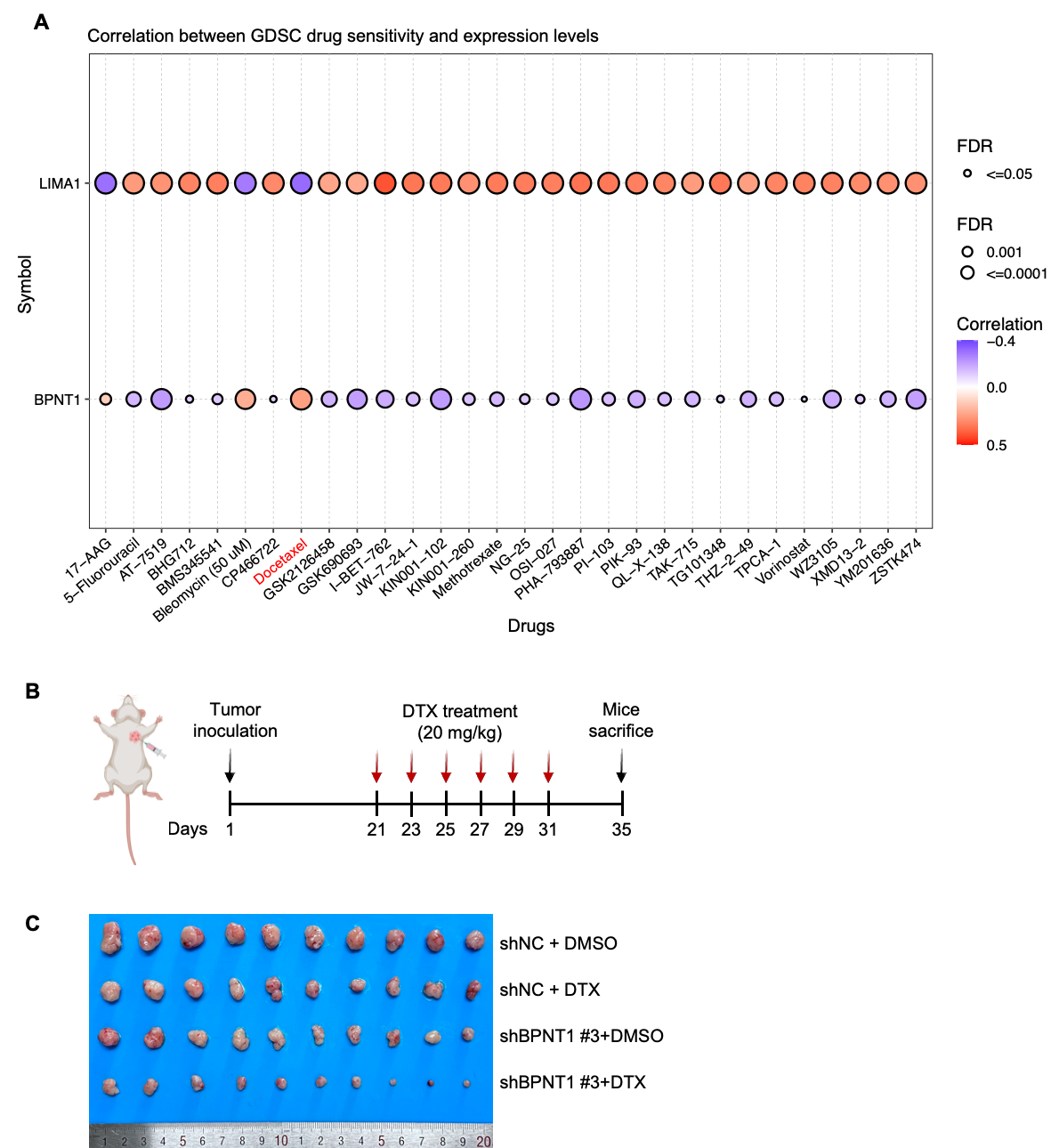
**

**Figure S7. BPNT1 promotes resistance of TNBC cells to docetaxel**

(A) Analysis of correlation between drug sensitivity and BPNT1 and LIMA1 expression in Genomics of Drug Sensitivity in Cancer (GDSC) dataset (https://www.cancerrxgene.org/).

(B-C) SUM-159 cells stably expressing shNC or shBPNT1 (#3) were inoculated into mammary fat pad of 6-week-old BALB/c female nude mice (*n* =20). After 20 days post-injection, mice were randomly divided into two groups (*n* =10), and administered with or without docetaxel (20 mg/kg, dissolved in DMSO) via intraperitoneal injection every 2 days for a total of 6 doses. Images of tumor growth and drug procedure (B) and representative tumor image (C) are shown. Left image in B was created using BioRender.com.

**Supplementary tables**

**Table S1. Targeting sequences for shRNAs**

| shRNAs | Primers | Sequences |
| --- | --- | --- |
| shBPNT1 #1 | Forward | CCGGCCATCGCAGTACAGTGCTATTCTCGAGAATAGCACTGTACTGCGATGGTTTTTG |
|  | Reverse | AATTCAAAAACCATCGCAGTACAGTGCTATTCTCGAGAATAGCACTGTACTGCGATGG |
| shBPNT1 #2 | Forward | CCGGAGCCGAGTTCCAGAATCTATTCTCGAGAATAGATTCTGGAACTCGGCTTTTTTG |
|  | Reverse | AATTCAAAAAAGCCGAGTTCCAGAATCTATTCTCGAGAATAGATTCTGGAACTCGGCT |
| shBPNT1#3 | Forward | CCGGCTTCTTGACAATGTAACAGTTCTCGAGAACTGTTACATTGTCAAGAAGTTTTTG |
|  | Reverse | AATTCAAAAACTTCTTGACAATGTAACAGTTCTCGAGAACTGTTACATTGTCAAGAAG |
| shSTUB1 #1 | Forward | CCGGCCCAAGTTCTGCTGTTGGACTCTCGAGAGTCCAACAGCAGAACTTGGGTTTTTG |
|  | Reverse | AATTCAAAAACCCAAGTTCTGCTGTTGGACTCTCGAGAGTCCAACAGCAGAACTTGGG |
| shSTUB1 #2 | Forward | CCGGCGCGAAGAAGAAGCGCTGGAACTCGAGTTCCAGCGCTTCTTCTTCGCGTTTTTG |
|  | Reverse | AATTCAAAAACGCGAAGAAGAAGCGCTGGAACTCGAGTTCCAGCGCTTCTTCTTCGCG |

**Table S2. Primers used for molecular cloning of expression vectors**

| Plasmids | Primers | Sequences |
| --- | --- | --- |
| Flag-pCDH-BPNT1 | Forward | GATTCTAGAGCTAGCGAATTCGCCACCATGGCTTCCAGTAACACTGTGTTG |
|  | Reverse | ATGGTCTTTGTAGTCGGATCCAGGAACAAGTGCATTTTTAATAGATTCTGG |
| Flag-pCDH-BPNT1^D51A^ | Forward | CAGACCAAAGCTGCCCGATTGGCACAG |
|  | Reverse | CTGTGCCAATCGGGCAGCTTTGGTCTG |
| Flag-pCDH-  BPNT1ΔC | Forward | GATTCTAGAGCTAGCGAATTCGCCACCATGGCTTCCAGTAACACTGTGTTG |
|  | Reverse | TGGTCTTTGTAGTCGGATCCCCCAAAGGCGCCTAAACC |
| Flag-pCDH-BPNT1ΔN | Forward | GATTCTAGAGCTAGCGAATTCGCCACCTTTCAGCTGAAAGAAGTCCCTGC |
|  | Reverse | ATGGTCTTTGTAGTCGGATCCAGGAACAAGTGCATTTTTAATAGATTCTGG |
| HA-pLVX-LIMA1 | Forward | GGATCTATTTCCGGTGAATTCGCCACCATGGAATCATCTCCATTTAATAGA |
|  | Reverse | GGGATCCGCGGCCGCTCTAGATTAAGCGTAGTCTGGGACGTCGTATGGGTACTCTTCATCCTCATCCTC |
| HA-pLVX-STUB1 | Forward | GGATCTATTTCCGGTGAATTCGCCACCATGCAGCAGCACGAGCAGGCC |
|  | Reverse | GGGATCCGCGGCCGCTCTAGATTAAGCGTAGTCTGGGACGTCGTATGGGTATCAGTAGTCCTCCACCCA |

**Table S3. Antibodies used in this study.**

| Antibodies | Vendors | Cat# | Host | Working concentration |
| --- | --- | --- | --- | --- |
| BPNT1 | Santa Cruz | sc-393185 | Mouse | 1:1000 (WB) |
| Vinculin | Sigma | V9131 | Mouse | 1:5000 (WB) |
| LIMA1 | Abclonal | A11682 | Rabbit | 1:1000 (WB) |
| STUB1 | Zenbio | R25823 | Rabbit | 1:1000 (WB) |
| Flag | Sigma | F1804 | Mouse | 1:3000 (WB)  1:200 (IF) |
| HA | CST | 3724S | Rabbit | 1:3000 (WB)  1:200 (IF) |
| P21 | CST | 2947S | Rabbit | 1:1000 (WB) |
| P62 | Abcam | ab109012 | Rabbit | 1:5000 (WB) |
| Ub | CST | 3936S | Mouse | 1:1000 (WB) |
| E-cadherin | CST | 3195S | Rabbit | 1:1000 (WB) |
| N-cadherin | CST | 13116S | Rabbit | 1:1000 (WB) |
| Vimentin | Abcam | ab8069 | Mouse | 1:2000 (WB) |

Note: WB, Western blot; IF, immunofluorescence;

**Table S4. Primers for qPCR analysis**

| Genes | Primers | Sequences |
| --- | --- | --- |
| BPNT1 | Forward | ATGGCTTCCAGTAACACTGTG |
|  | Reverse | CGTCTGACTATCATTCCTGCC |
| LIMA1 | Forward | GACTCCCAGGTTAAGAGTGAGG |
|  | Reverse | TTGCAGGTGCCTGAAACTTCT |
| STUB1 | Forward | AGCAGGGCAATCGTCTGTTC |
|  | Reverse | CAAGGCCCGGTTGGTGTAATA |
| GAPDH | Forward | GGAGCGAGATCCCTCCAAAAT |
|  | Reverse | GGCTGTTGTCATACTTCTCATGG |

**Table S5. Reagents used in this study.**

| Reagents | Vendors | Cat# |
| --- | --- | --- |
| DMEM | BasalMedia | L110 |
| Fetal bovine serum | Gibco | 10270-106 |
| Neofect DNA transfection reagent | TEYE Biotech | TF201201 |
| BCA assay | Yeasen | 20201ES90 |
| PVDF membrane | Millipore | IPVH00010 |
| BSA | Sigma | 900933 |
| Chemiluminescence detection kit | Yeasen | 36208ES76 |
| RNAiso Plus reagent | Takara | 9109 |
| HiScript III RT SuperMix | Vazyme | 323-01 |
| ChamQ Universal SYBR qPCR Master Mix | Vazyme | Q711-03 |
| Docetaxel | Selleck | RP56976 |
| CCK-8 kit | Yeasen | 40203ES92 |
| Migration assay | BD Falcon | 353097 |
| Invasion assay | BD Biocoat | 354480 |
| Coverslip | Fisher Scientific | 12-545-80 |
| Paraformaldehyde | Sangon Biotech | E672002-0500 |
| Triton X-100 | Sigma-Aldrich | 93443 |
| DAPI-fluoroshield mounting medium | Abcam | ab104139 |
| MG-132 | Selleck Chemicals | 2619 |
