# Supplementary material for: Bisphosphate nucleotidase 1 promotes progression and docetaxel resistance in triple-negative breast cancer via STUB1-mediated destabilization of LIMA1
Source: Cell Death Dis. 2026 Jan 15;17(1):40. doi: 10.1038/s41419-025-08245-0 (PMC12808305; doi:10.1038/s41419-025-08245-0)

Figure 1K

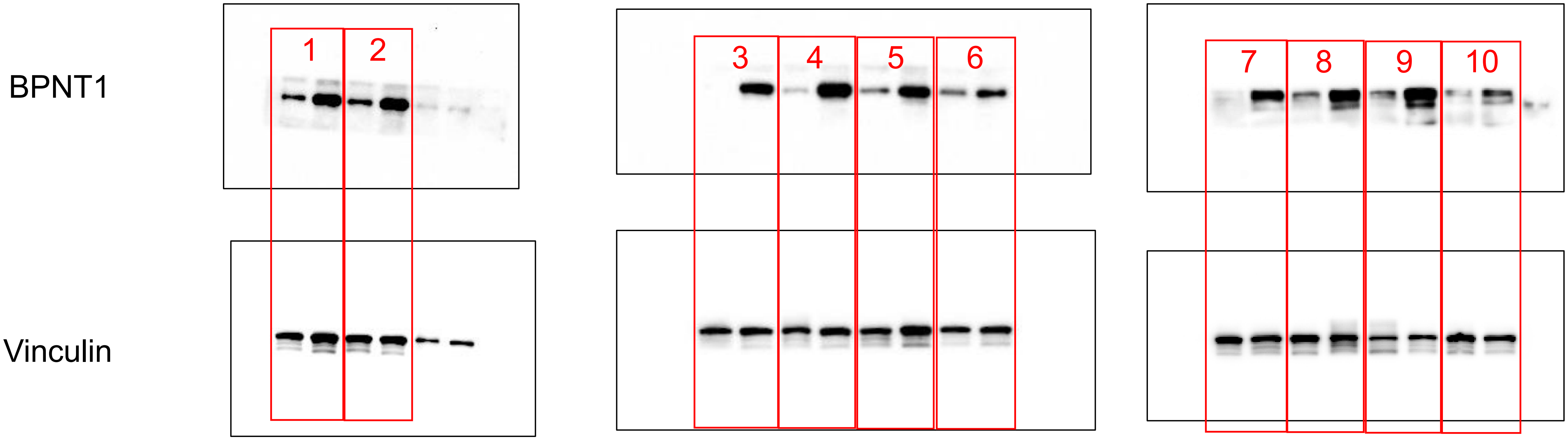

Figure 1M

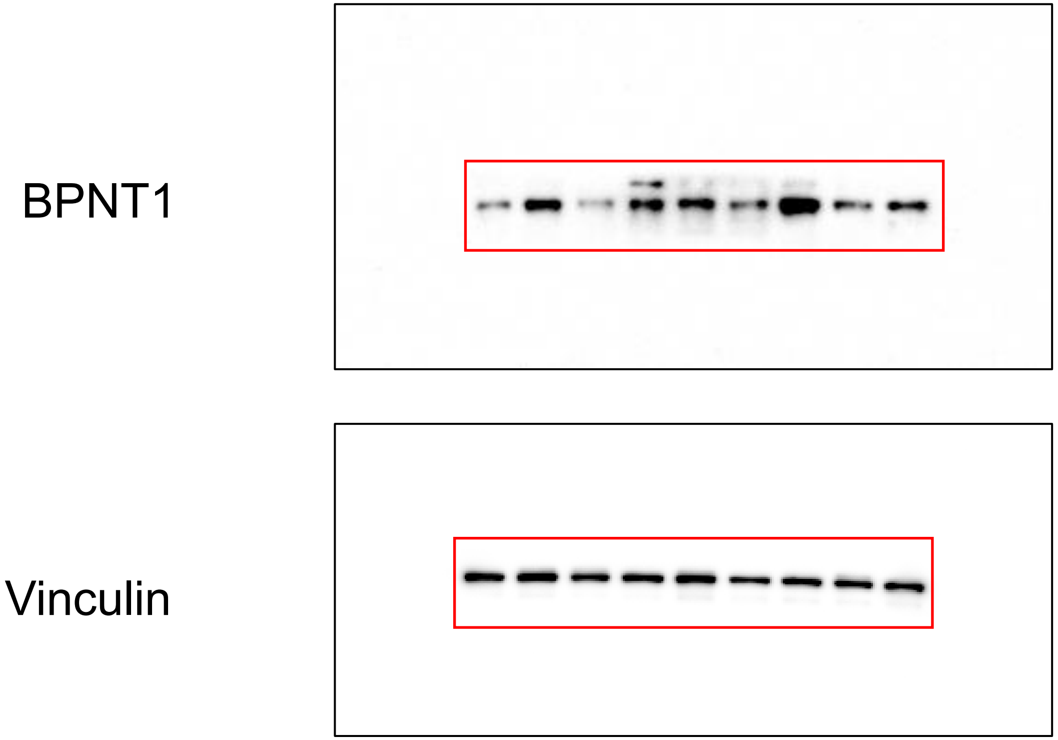

Figure 2A

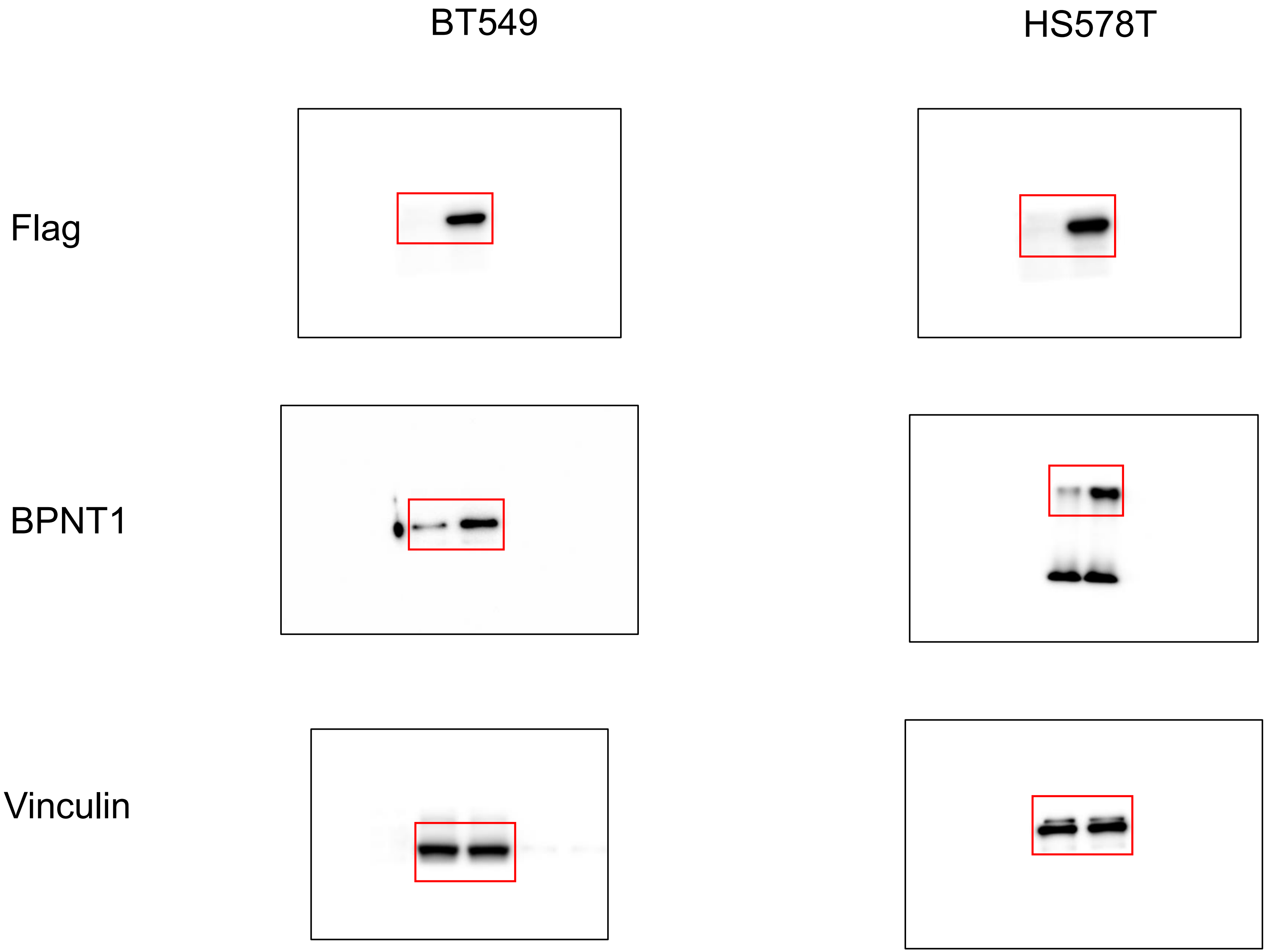

Figure 2E

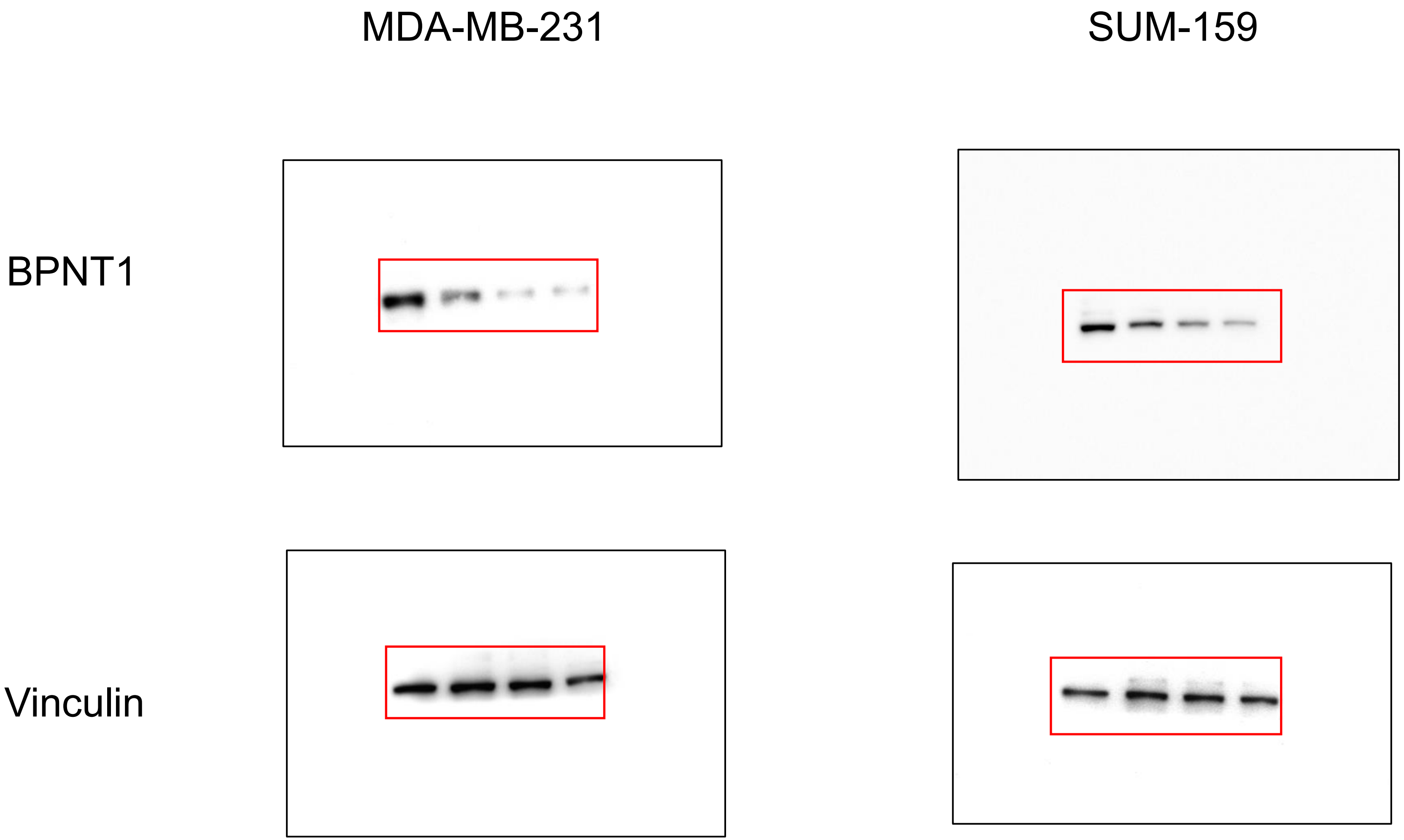

Figure 4B

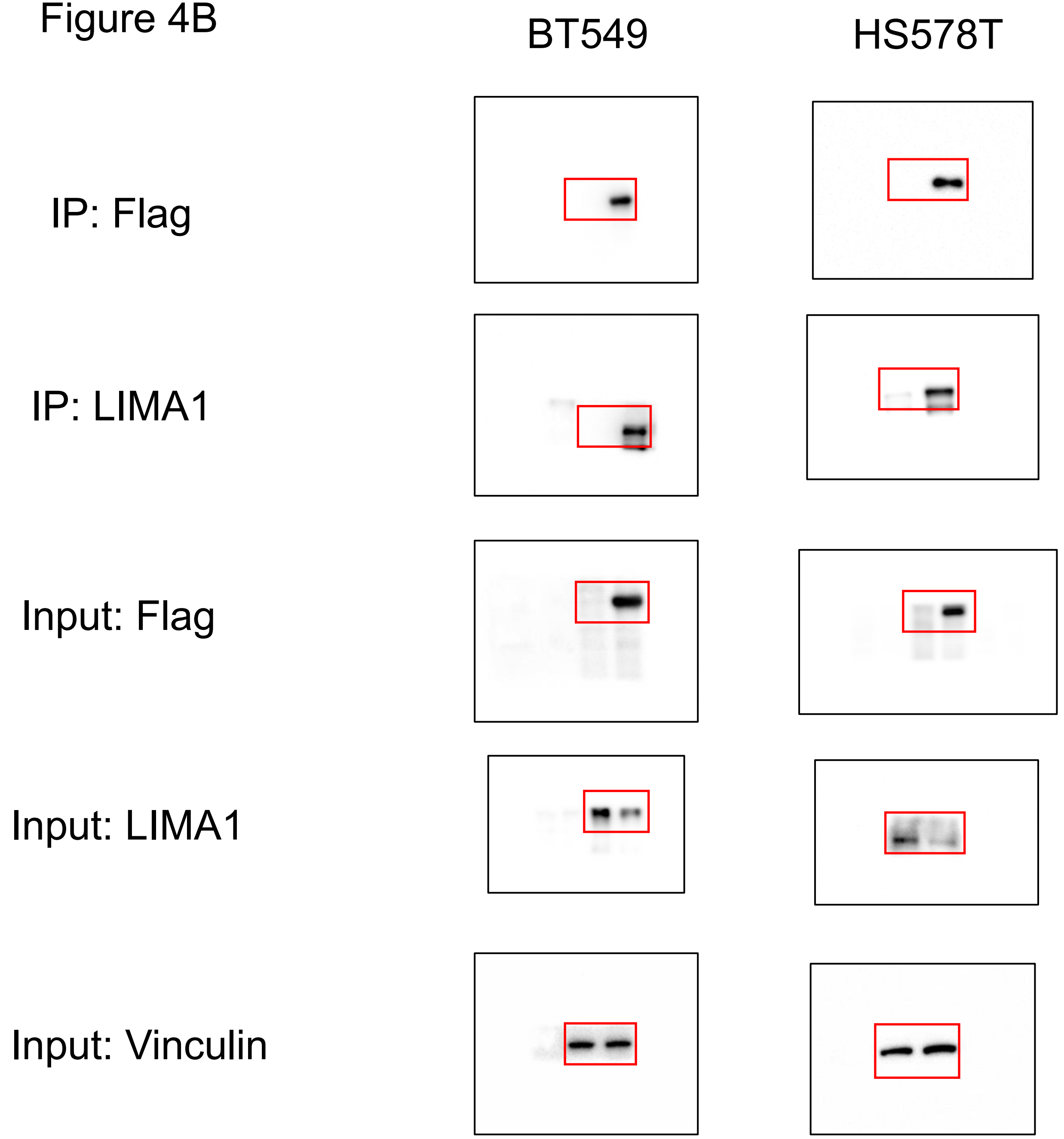
BT549
HS578T
IP: Flag
IP: LIMA1
Input: Flag
Input: LIMA1
Input: Vinculin

Figure 4C

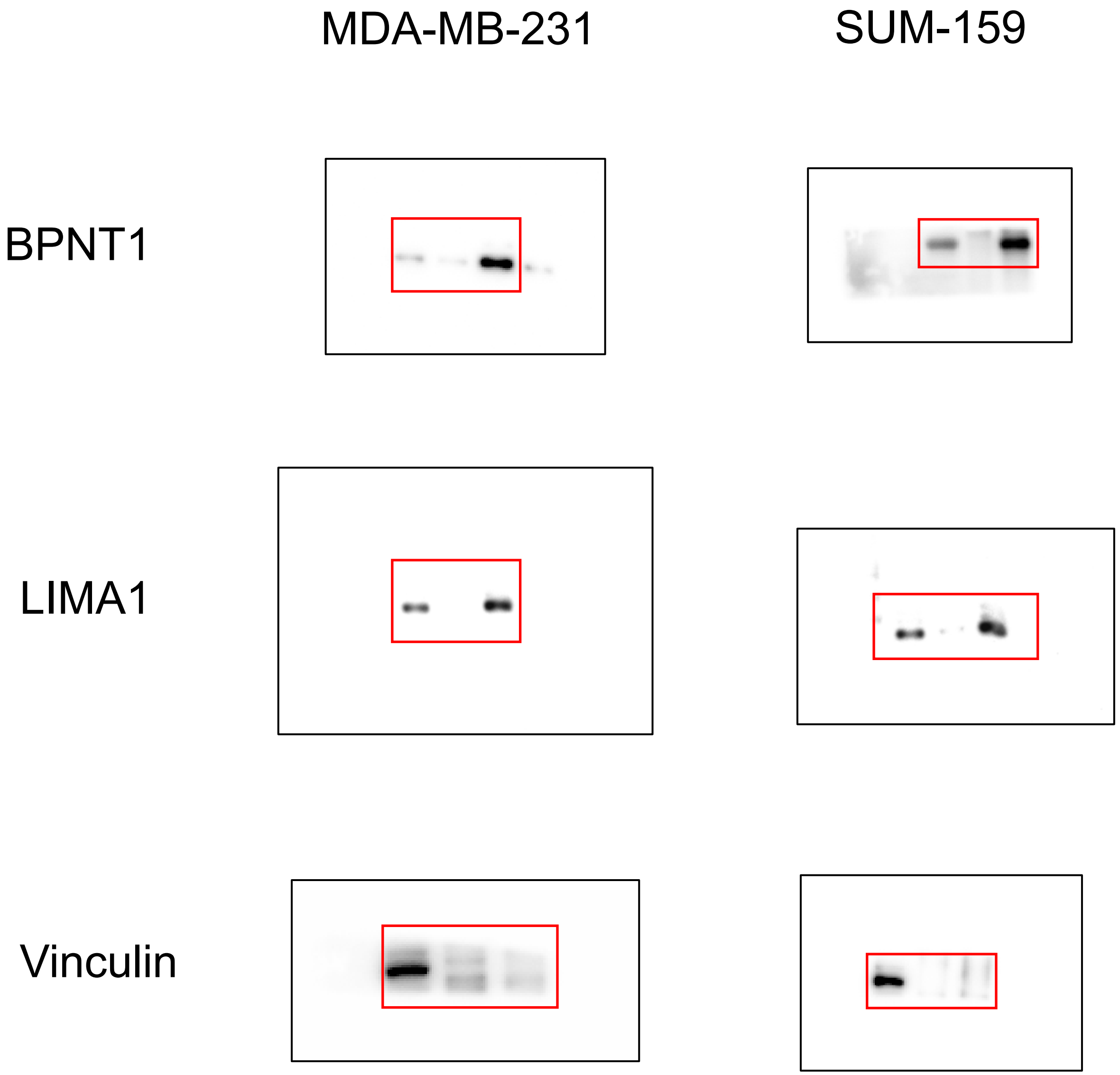

Figure 4D

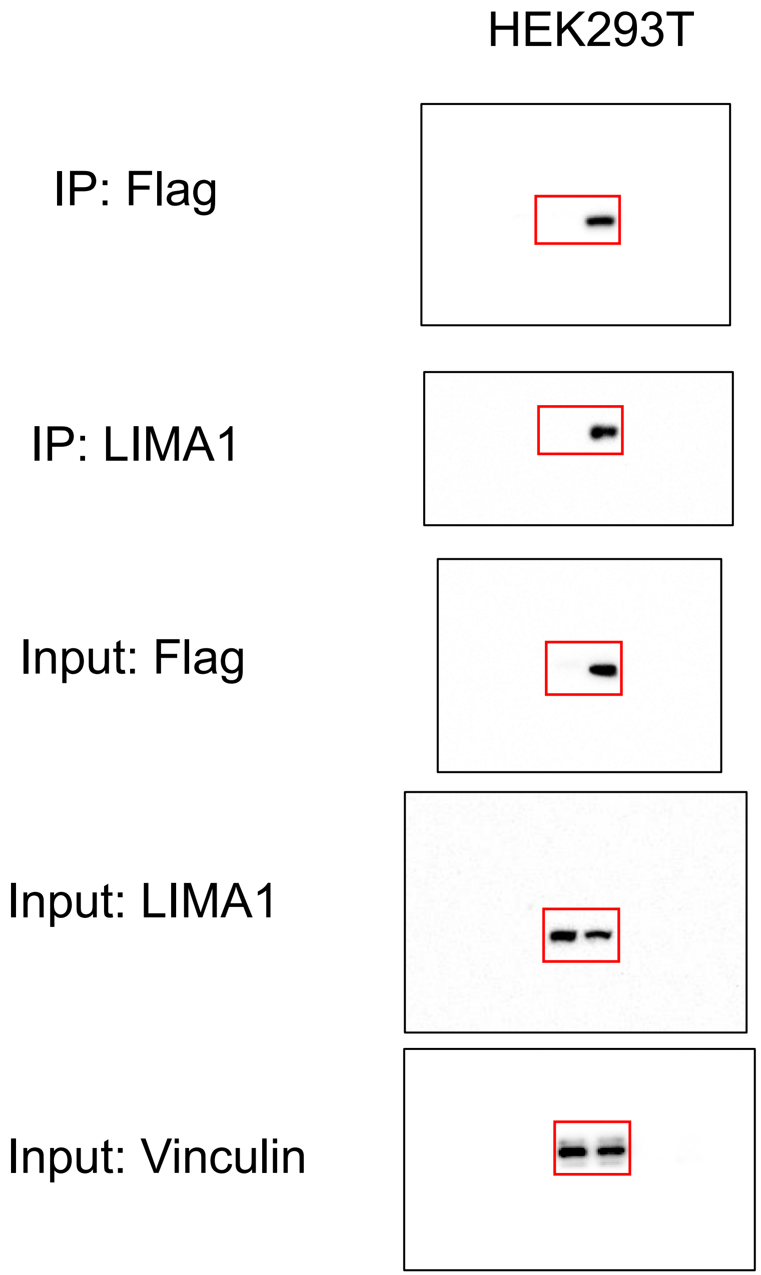
HEK293T
IP: Flag
IP: LIMA1
Input: Flag
Input: LIMA1
Input: Vinculin

Figure 4G

HEK293T

IP: HA

IP: Flag

Input: Flag

Input: HA

Input: Vinculin

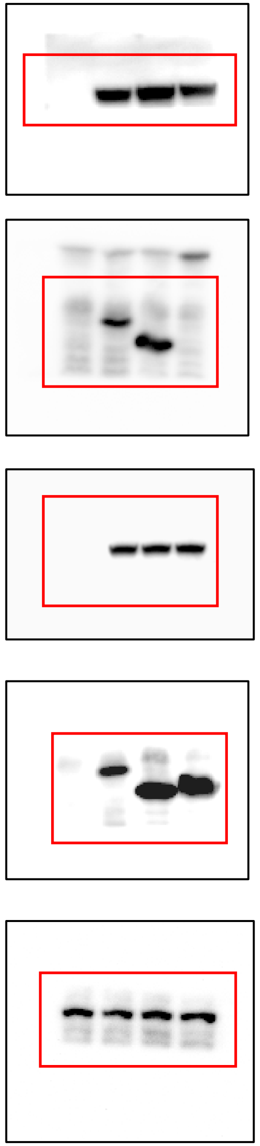

Figure 4H

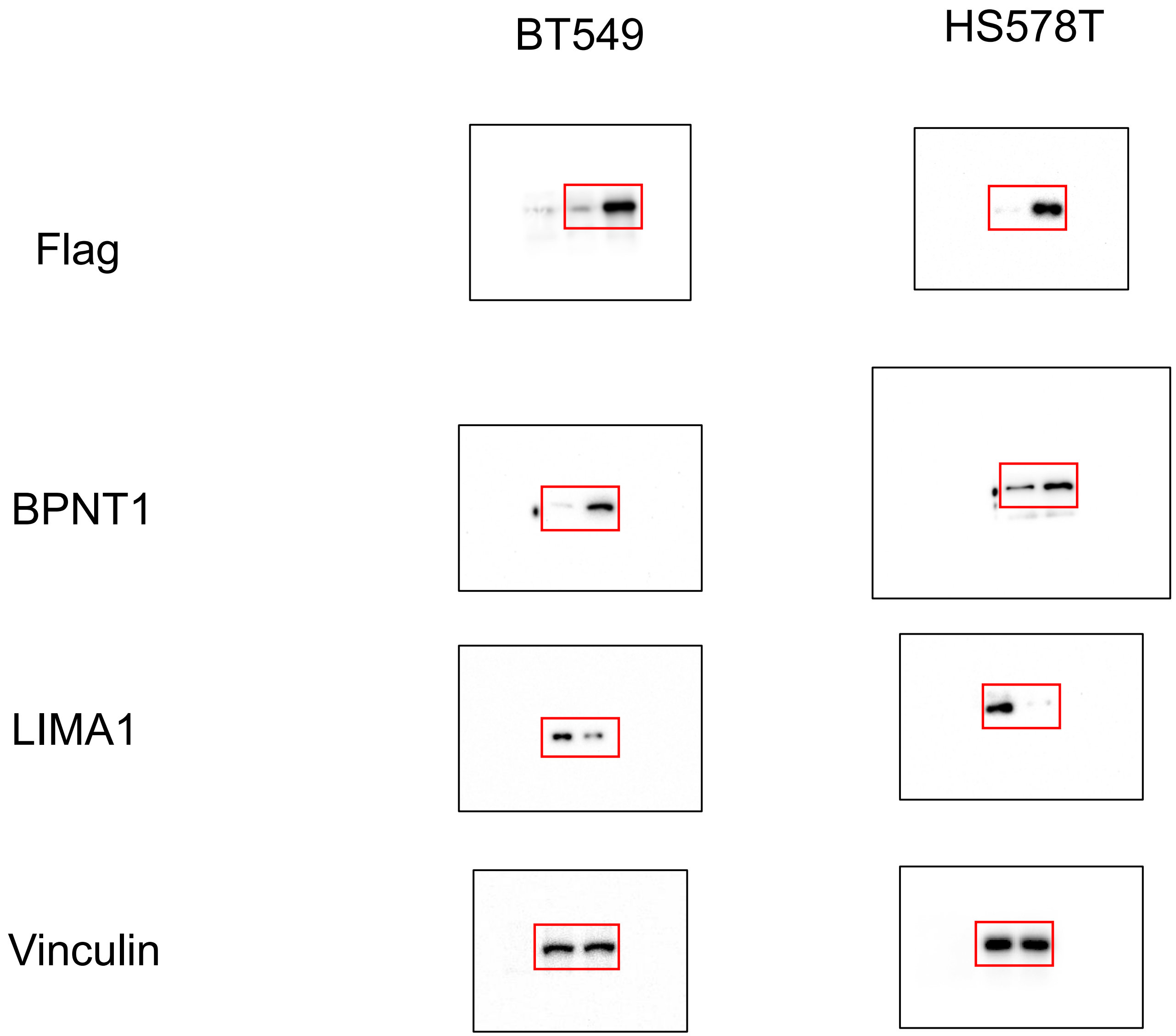

Figure 4I

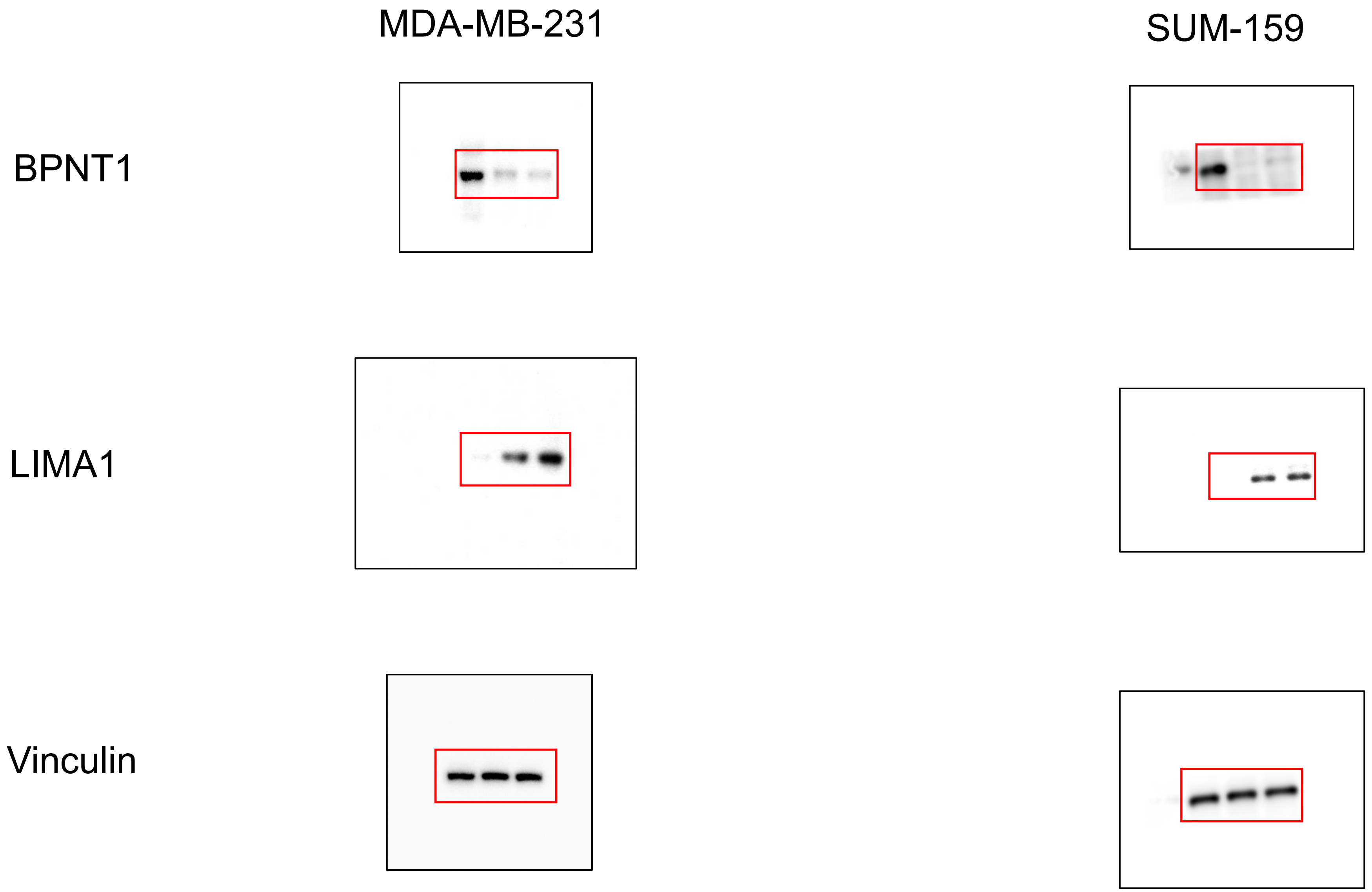

Figure 4J

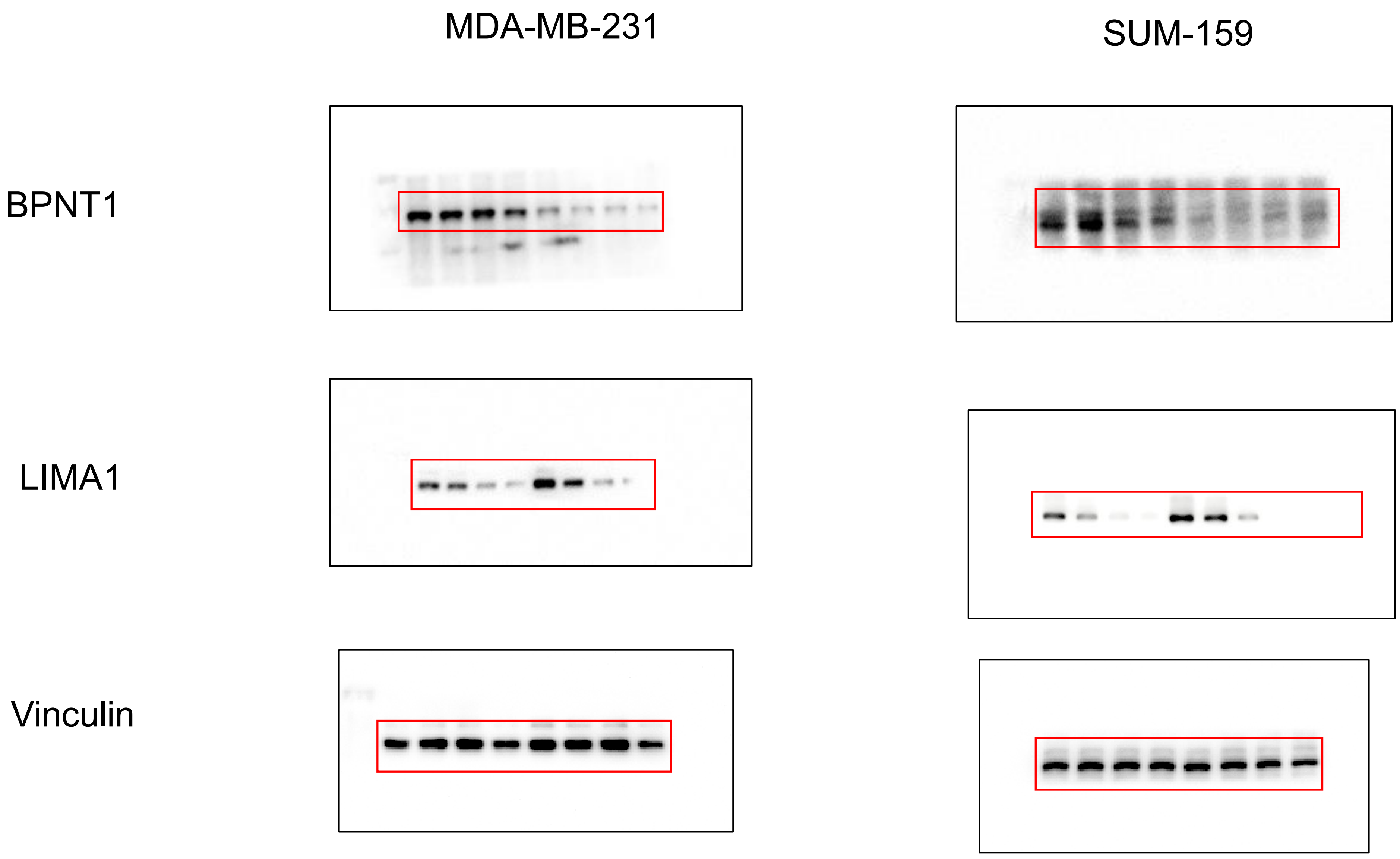

Figure 4L

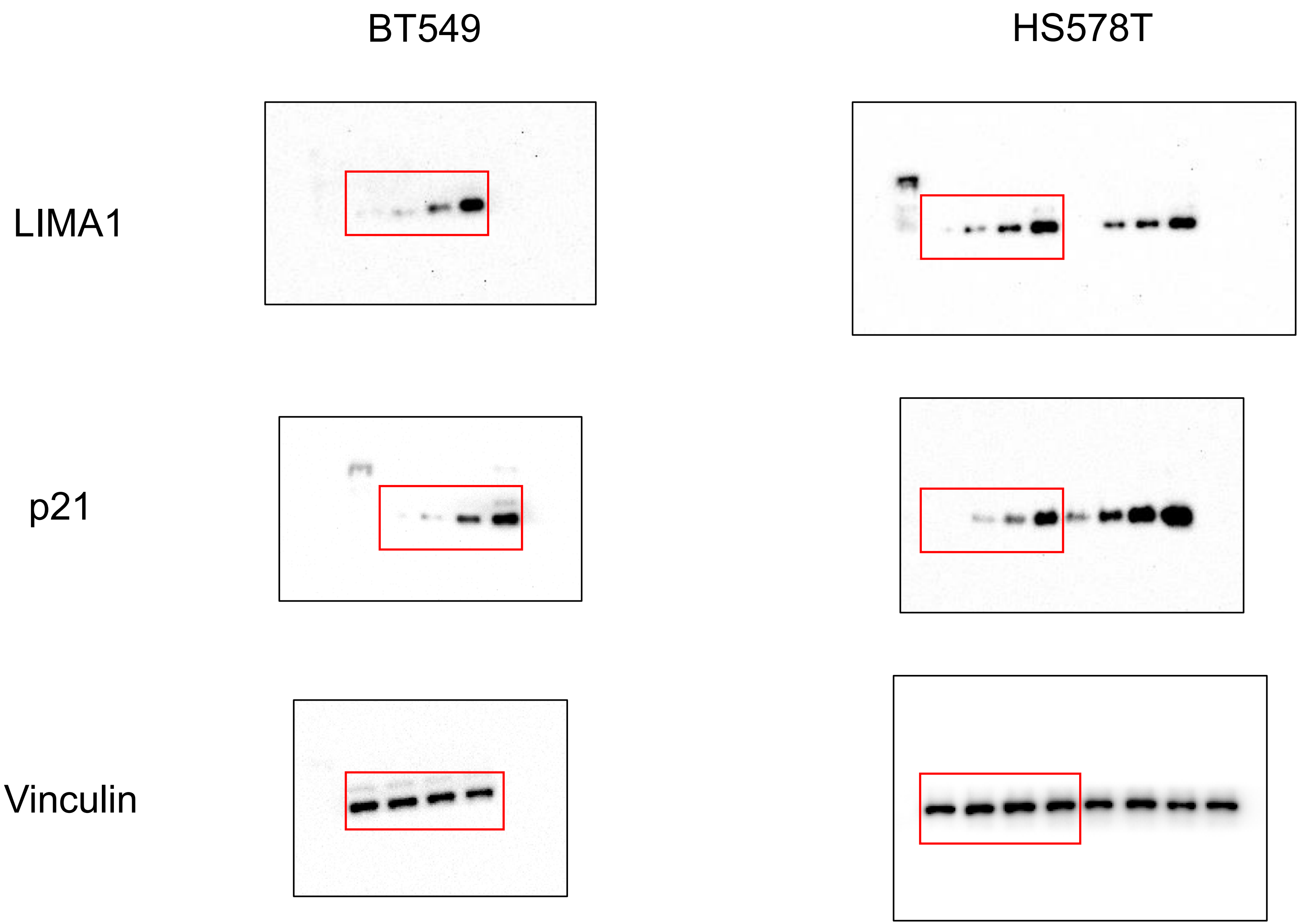

Figure 4M

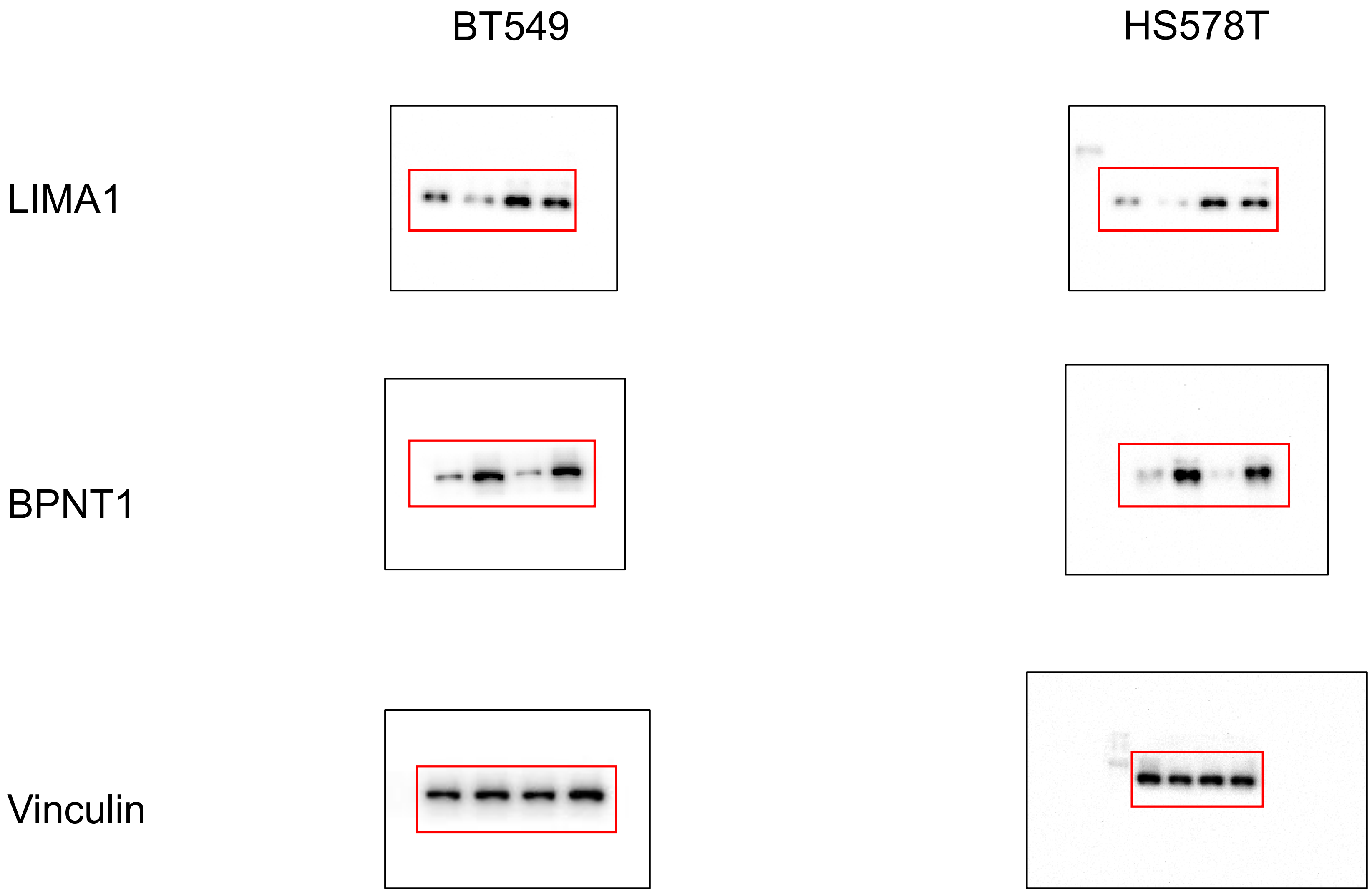

Figure 5A

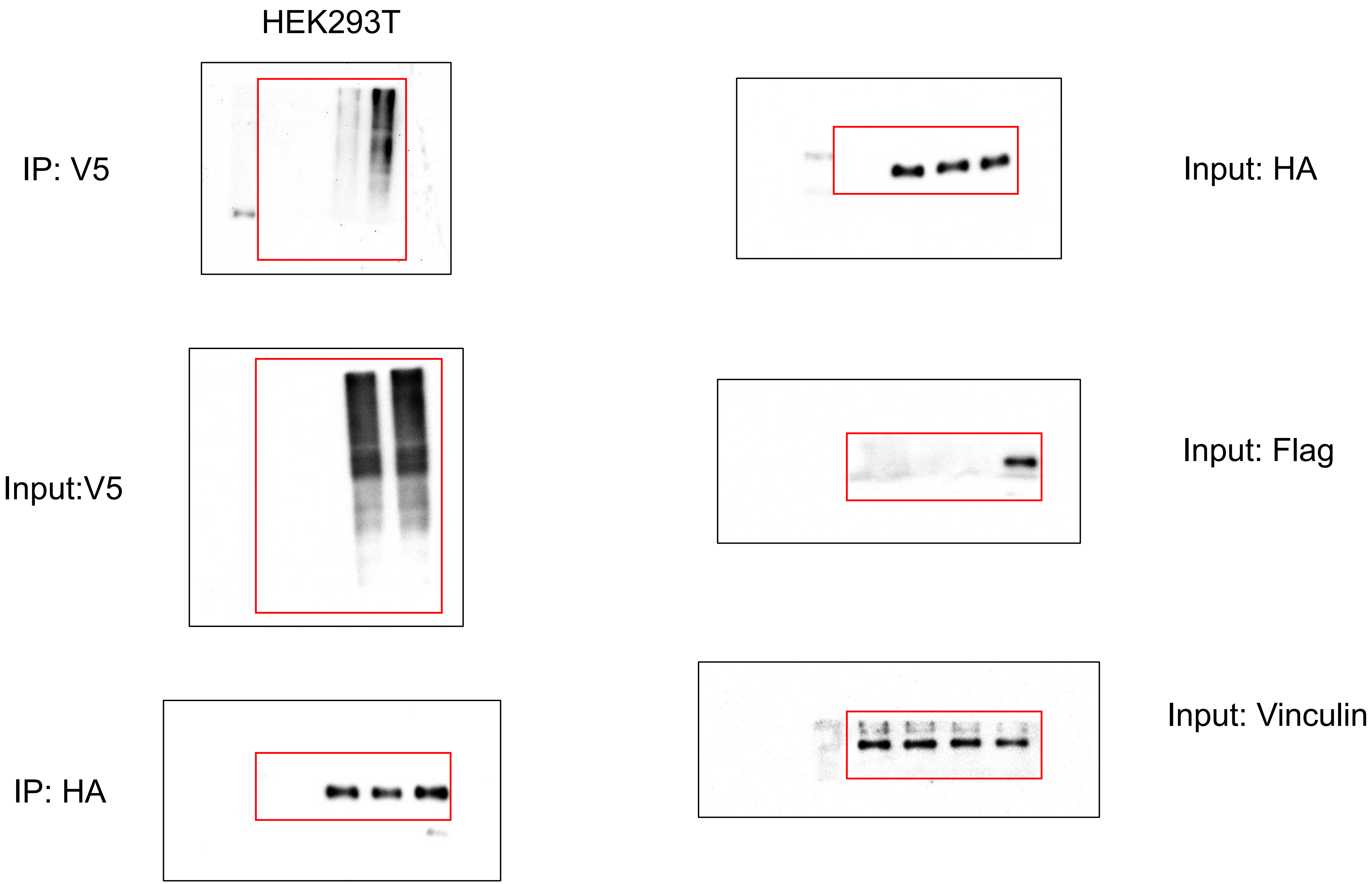
HEK293T
IP: V5
Input:V5
IP: HA
Input: HA
Input: Flag
Input: Vinculin

Figure 5B

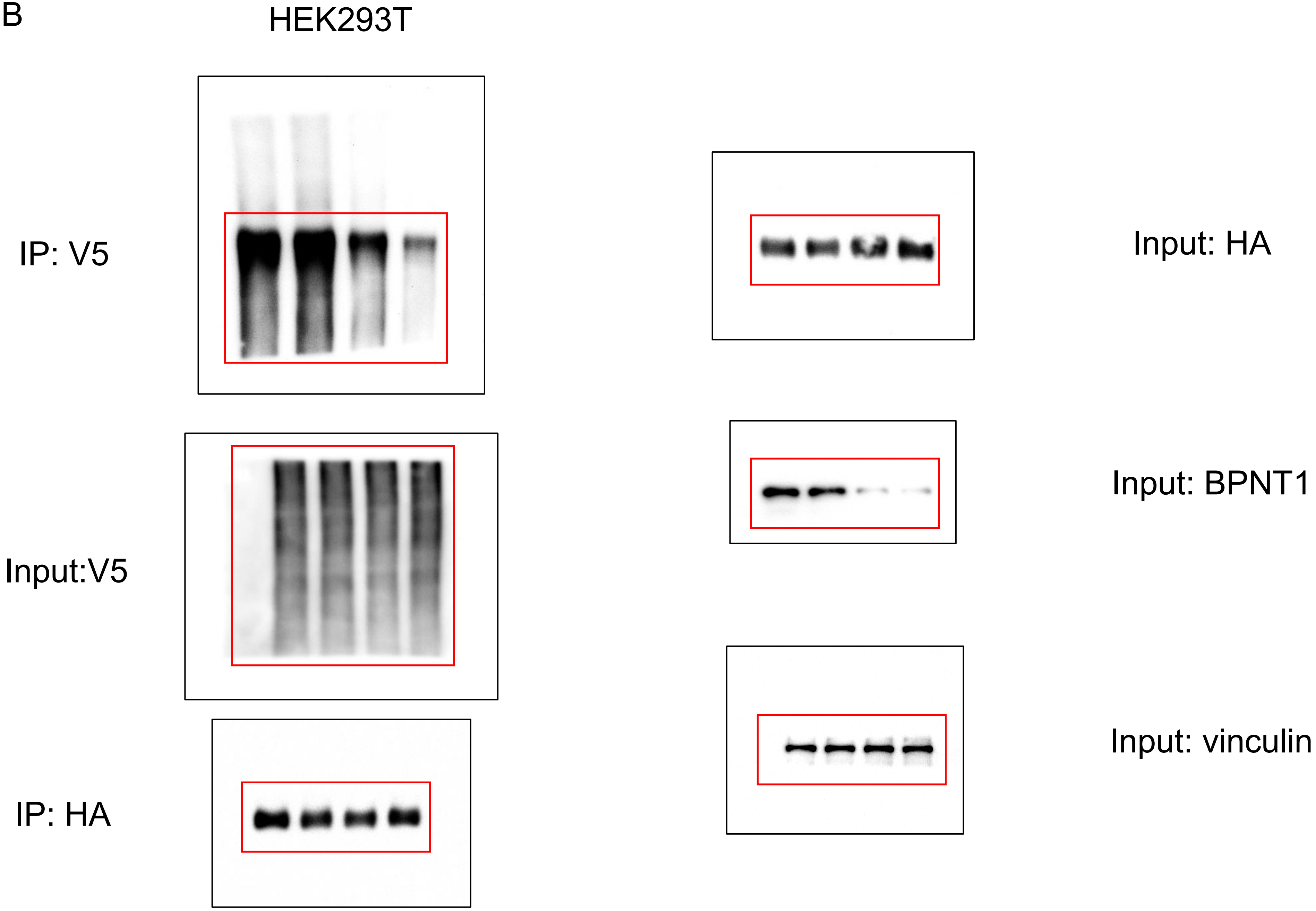
HEK293T
IP: V5
Input:V5
IP: HA
Input: HA
Input: BPNT1
Input: vinculin

Figure 5C

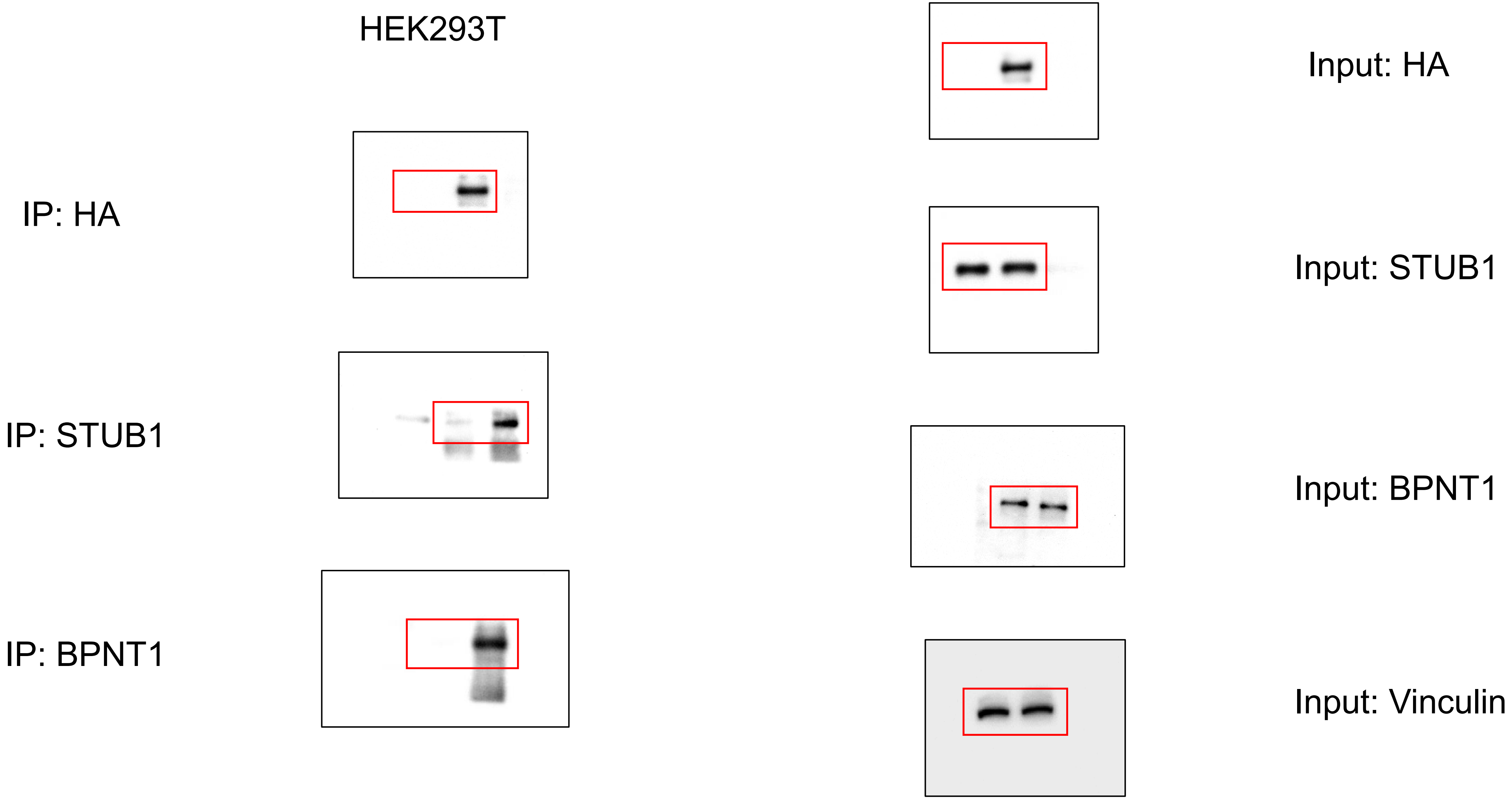
HEK293T
IP: HA
IP: STUB1
IP: BPNT1
Input: HA
Input: STUB1
Input: BPNT1
Input: Vinculin

Figure 5D

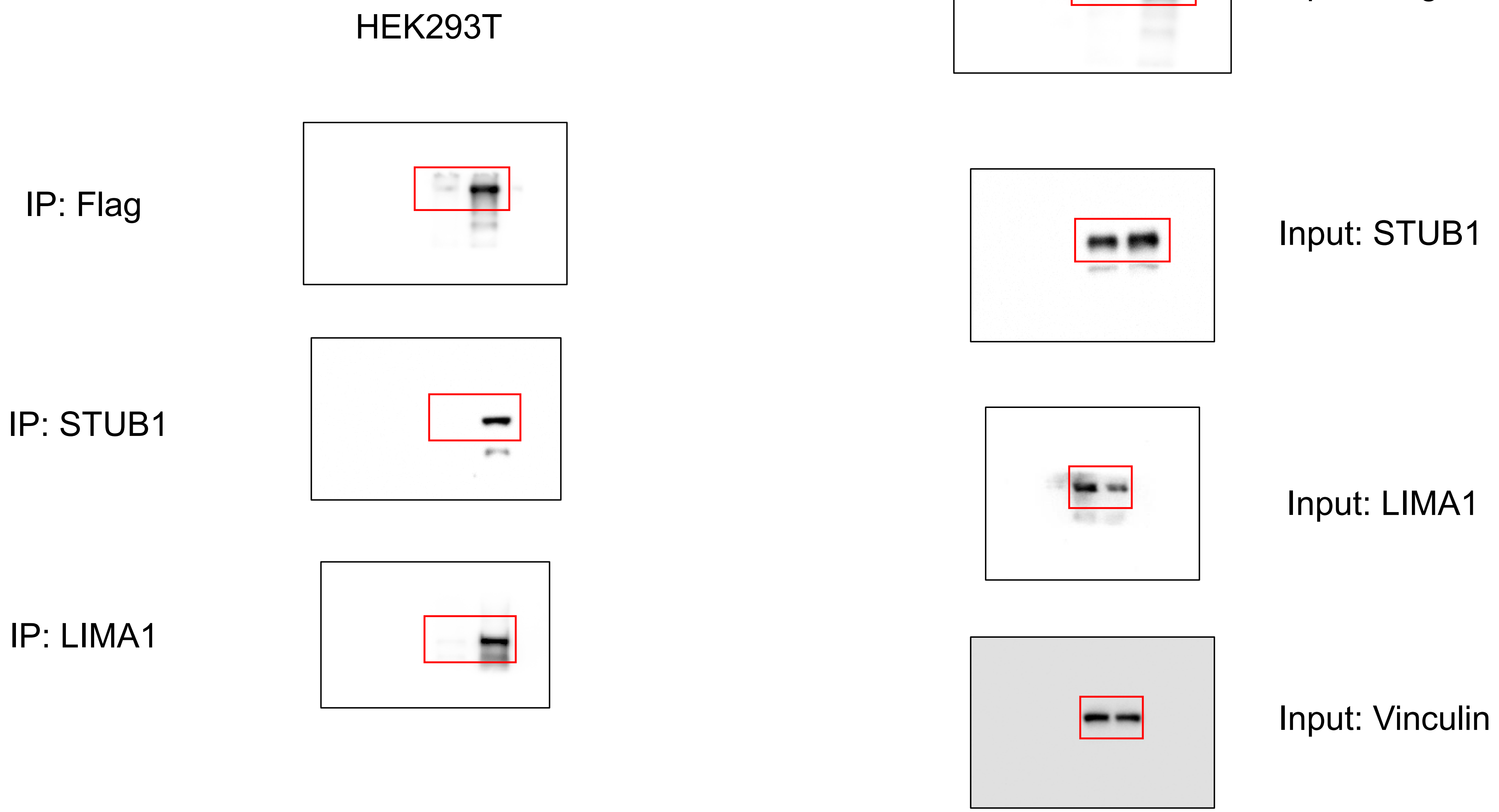
HEK293T
IP: Flag
IP: STUB1
IP: LIMA1
Input: Flag
Input: STUB1
Input: LIMA1
Input: Vinculin

Figure 5E

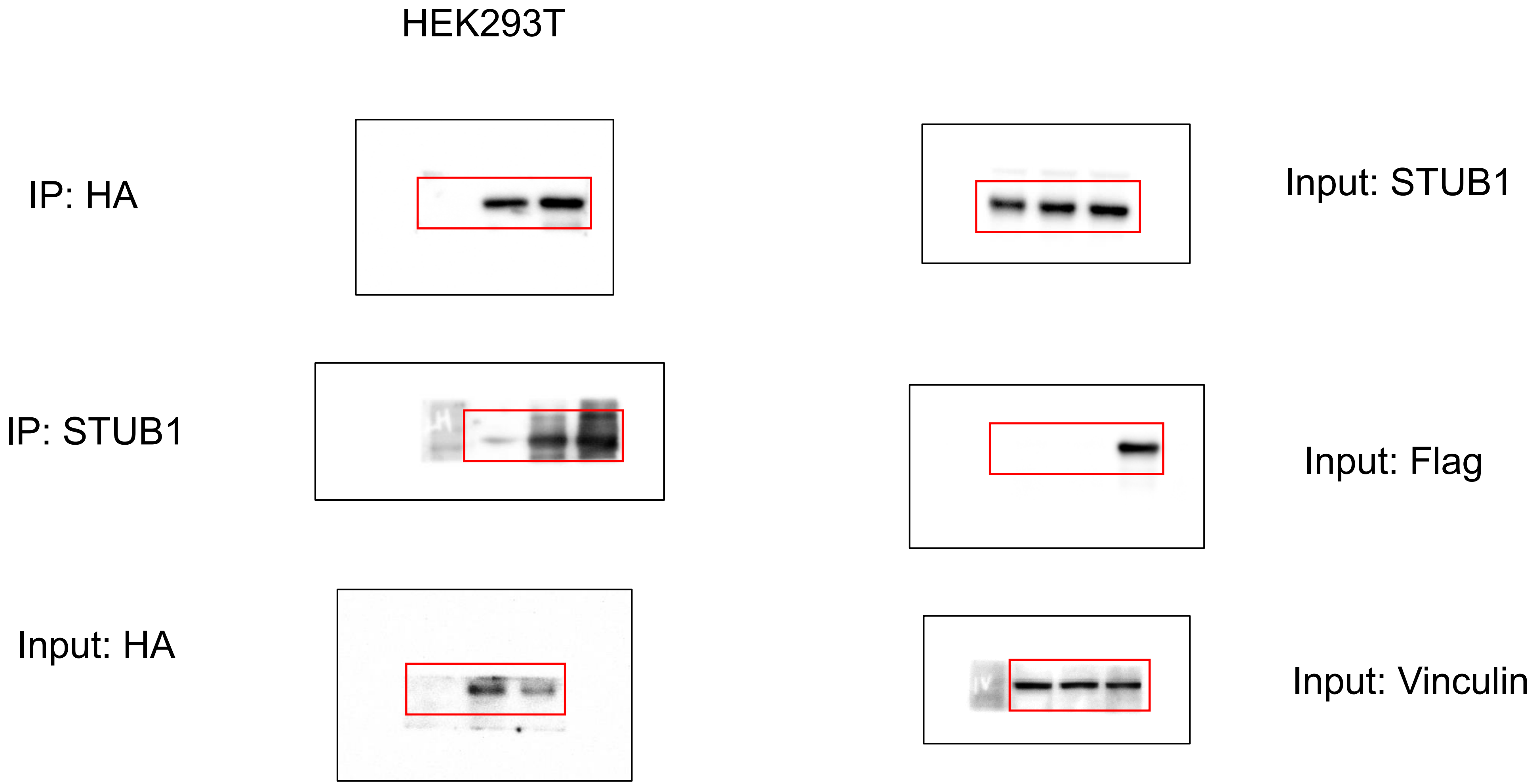
HEK293T
IP: HA
Input: STUB1
IP: STUB1
Input: Flag
Input: HA
Input: Vinculin

Figure 5F

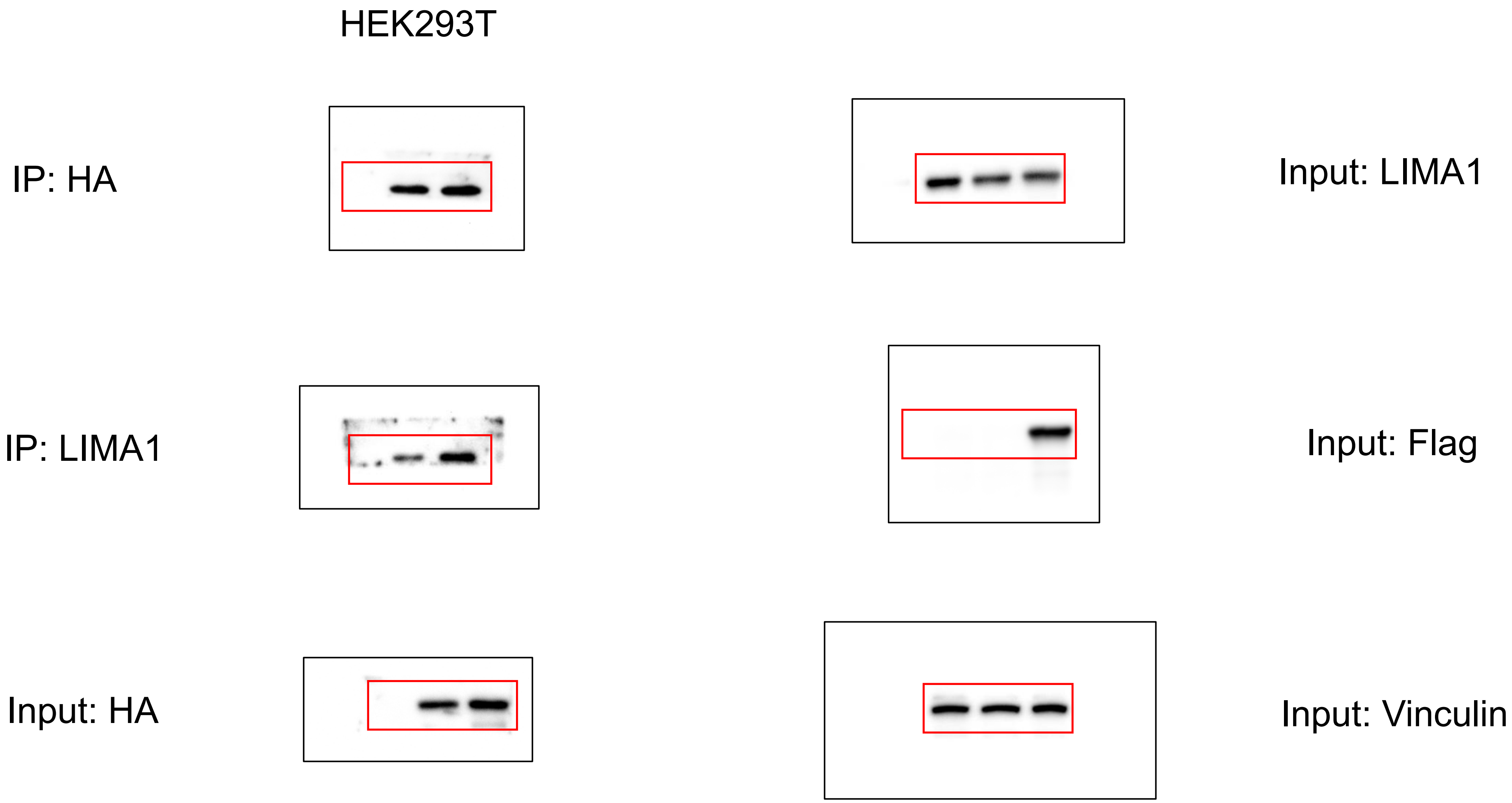
HEK293T
IP: HA
Input: LIMA1
IP: LIMA1
Input: Flag
Input: HA
Input: Vinculin

Figure 5G

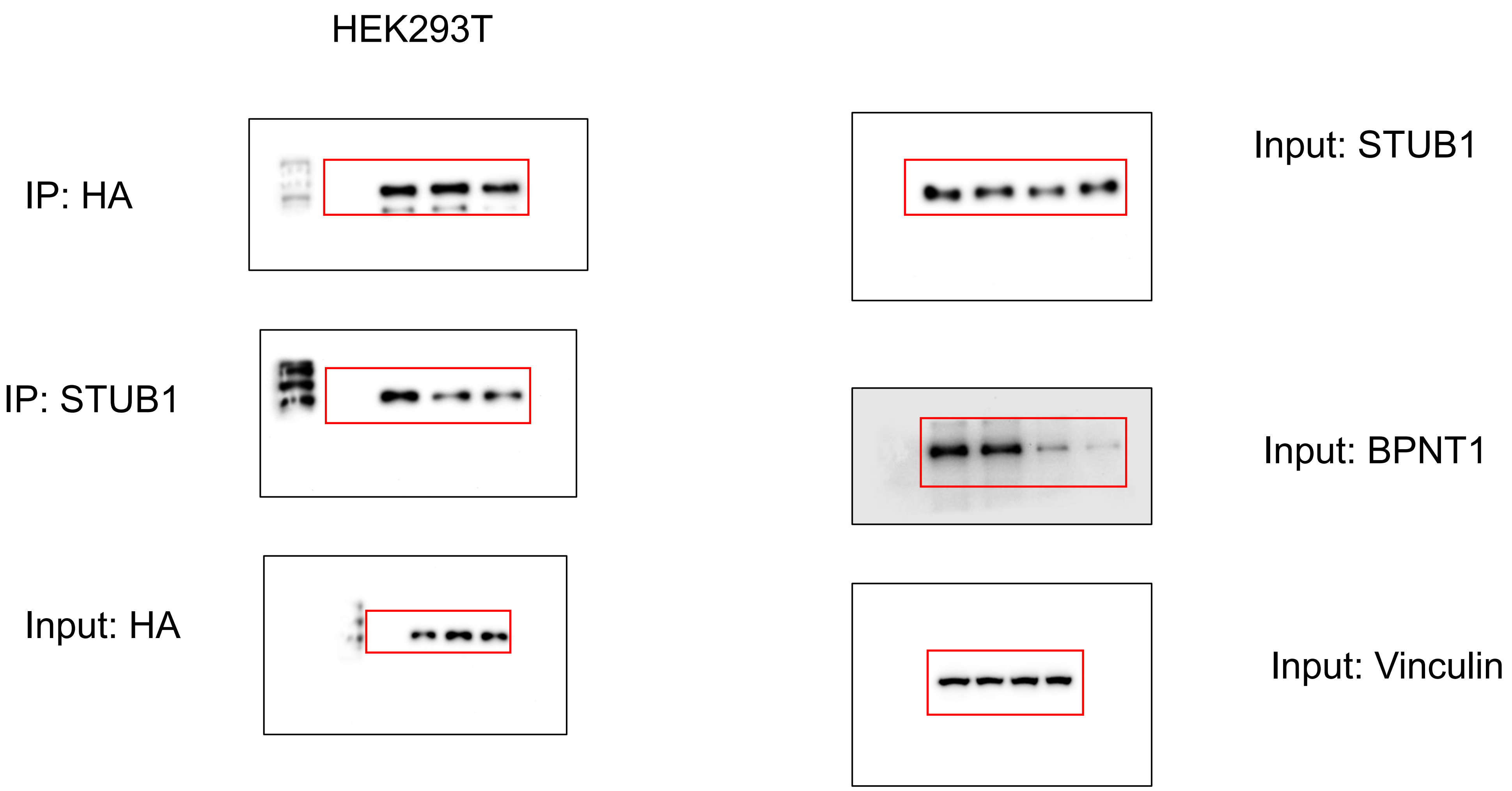
HEK293T
IP: HA
IP: STUB1
Input: HA
Input: STUB1
Input: BPNT1
Input: Vinculin

Figure 5H

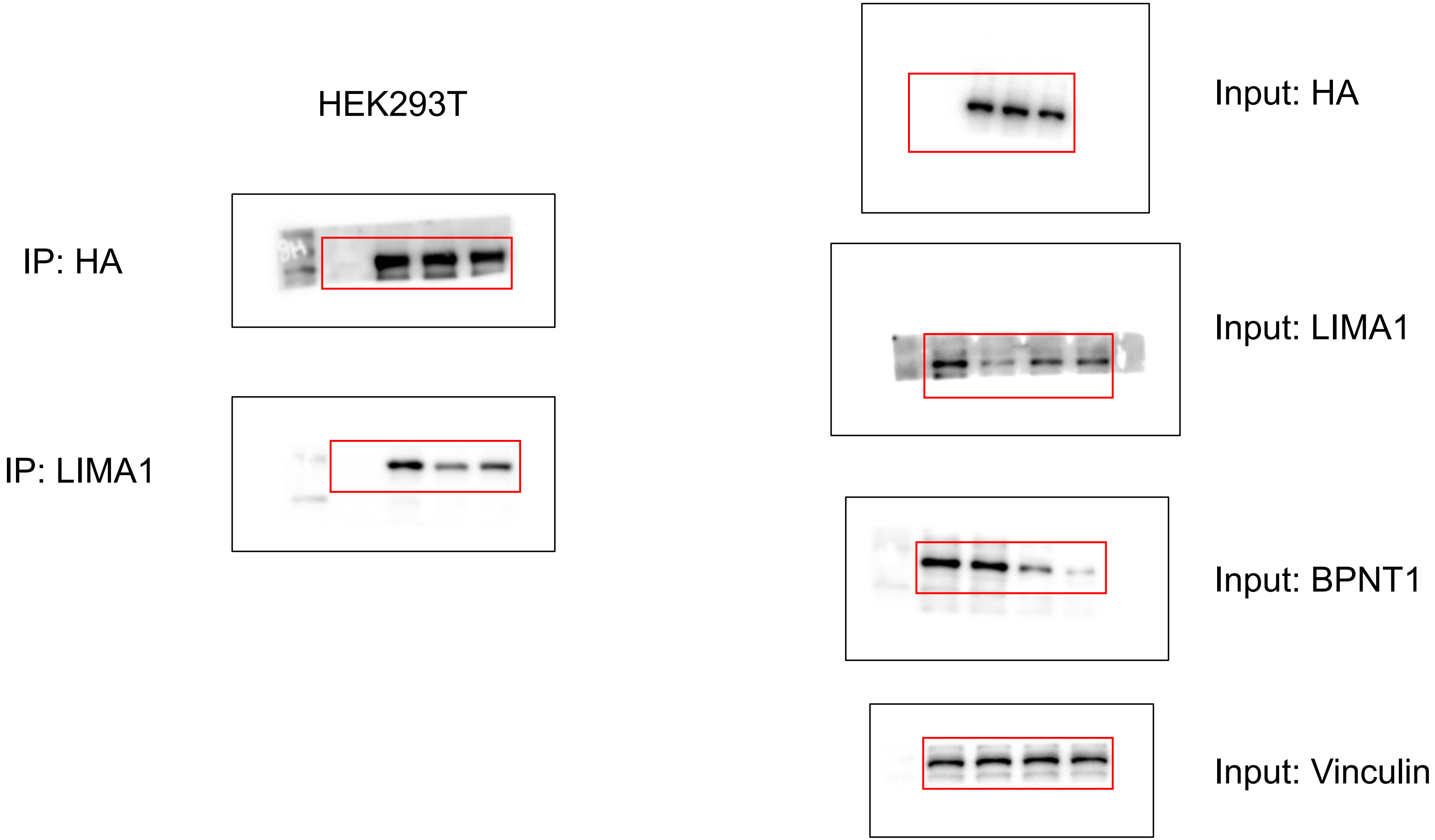
HEK293T
IP: HA
IP: LIMA1
Input: HA
Input: LIMA1
Input: BPNT1
Input: Vinculin

Figure 5I

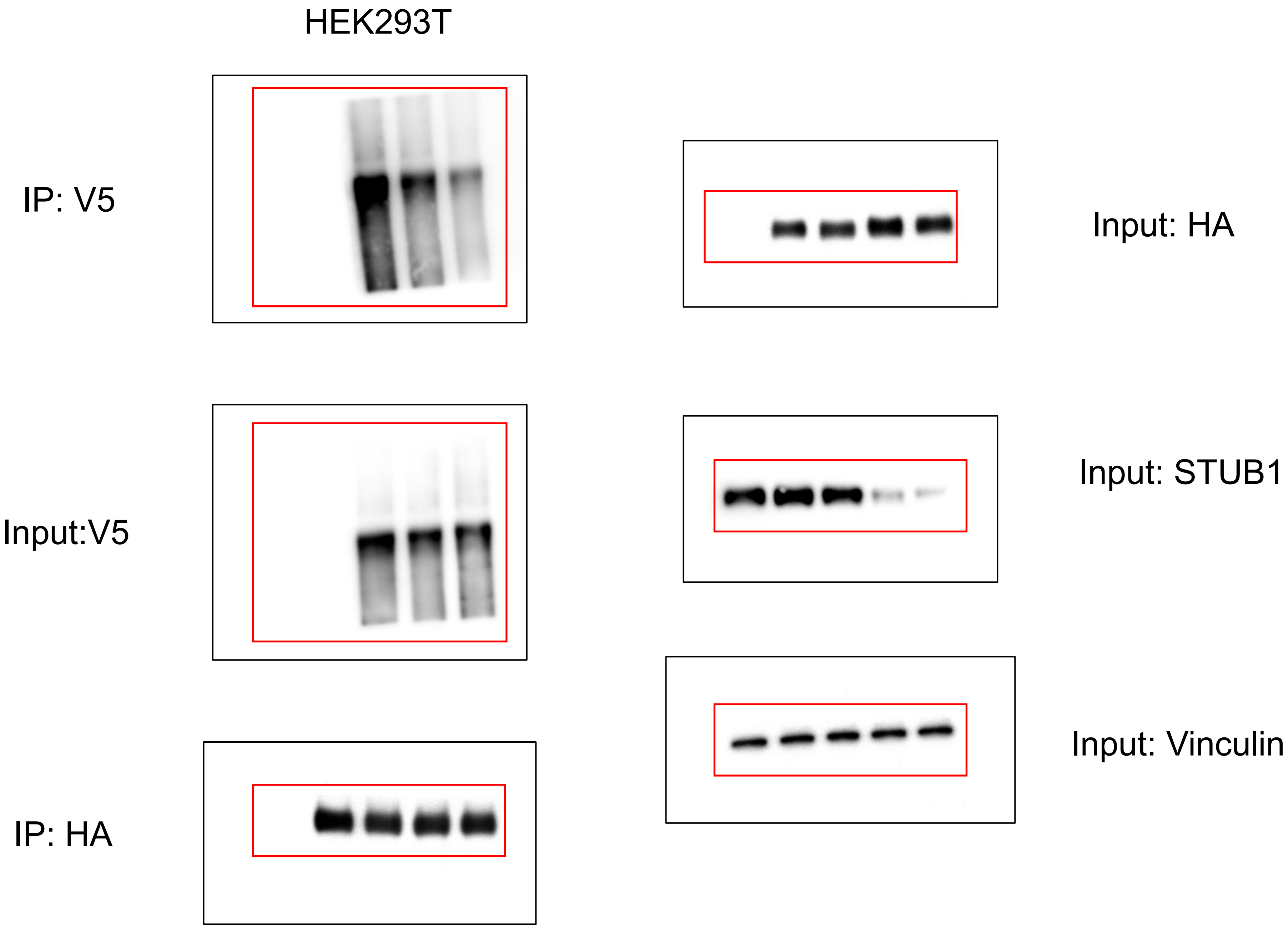
HEK293T
IP: V5
Input: HA
Input: STUB1
Input:V5
Input: Vinculin
IP: HA

Figure 5J

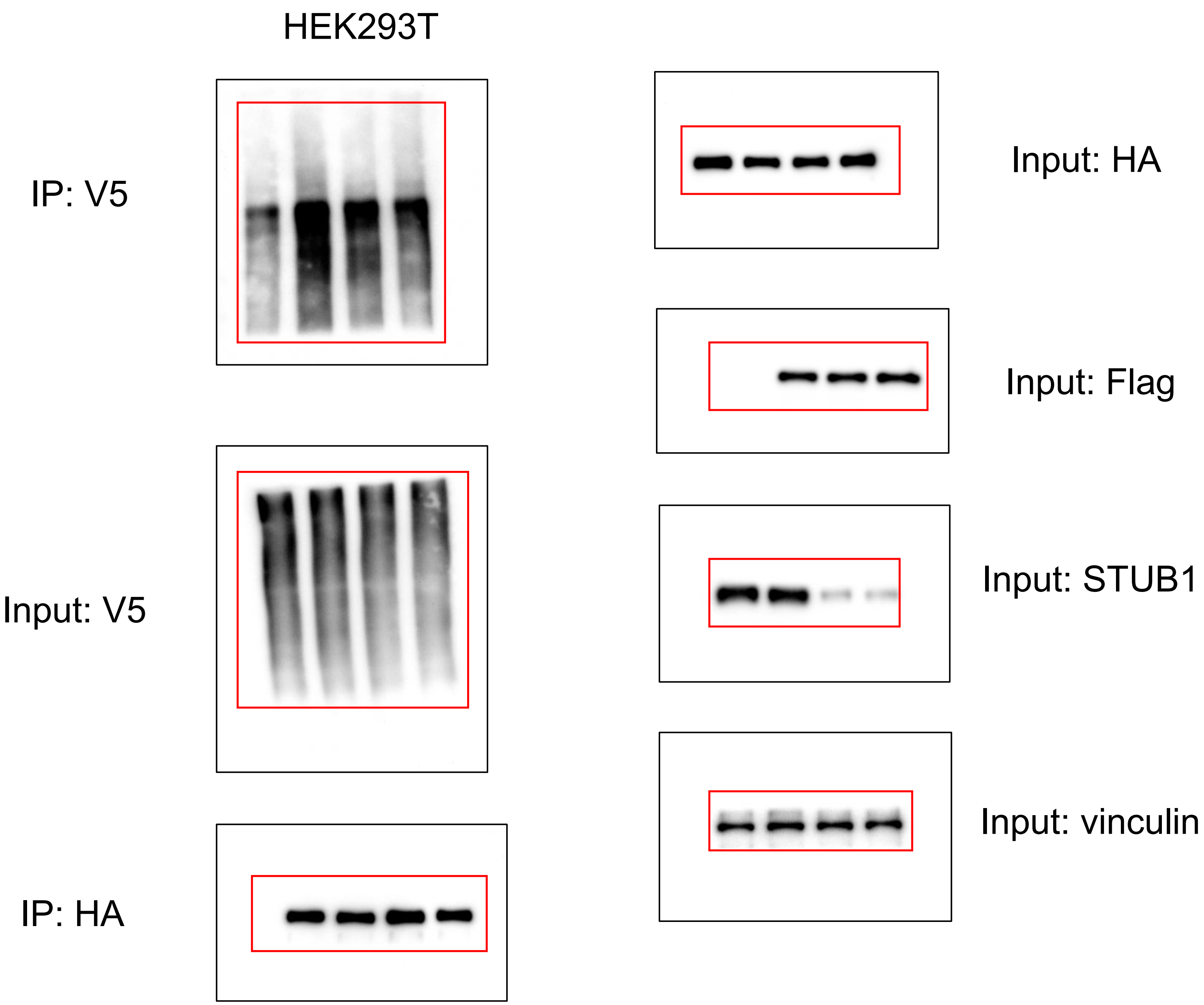
HEK293T
IP: V5
Input: V5
IP: HA
Input: HA
Input: Flag
Input: STUB1
Input: vinculin

Figure 6A

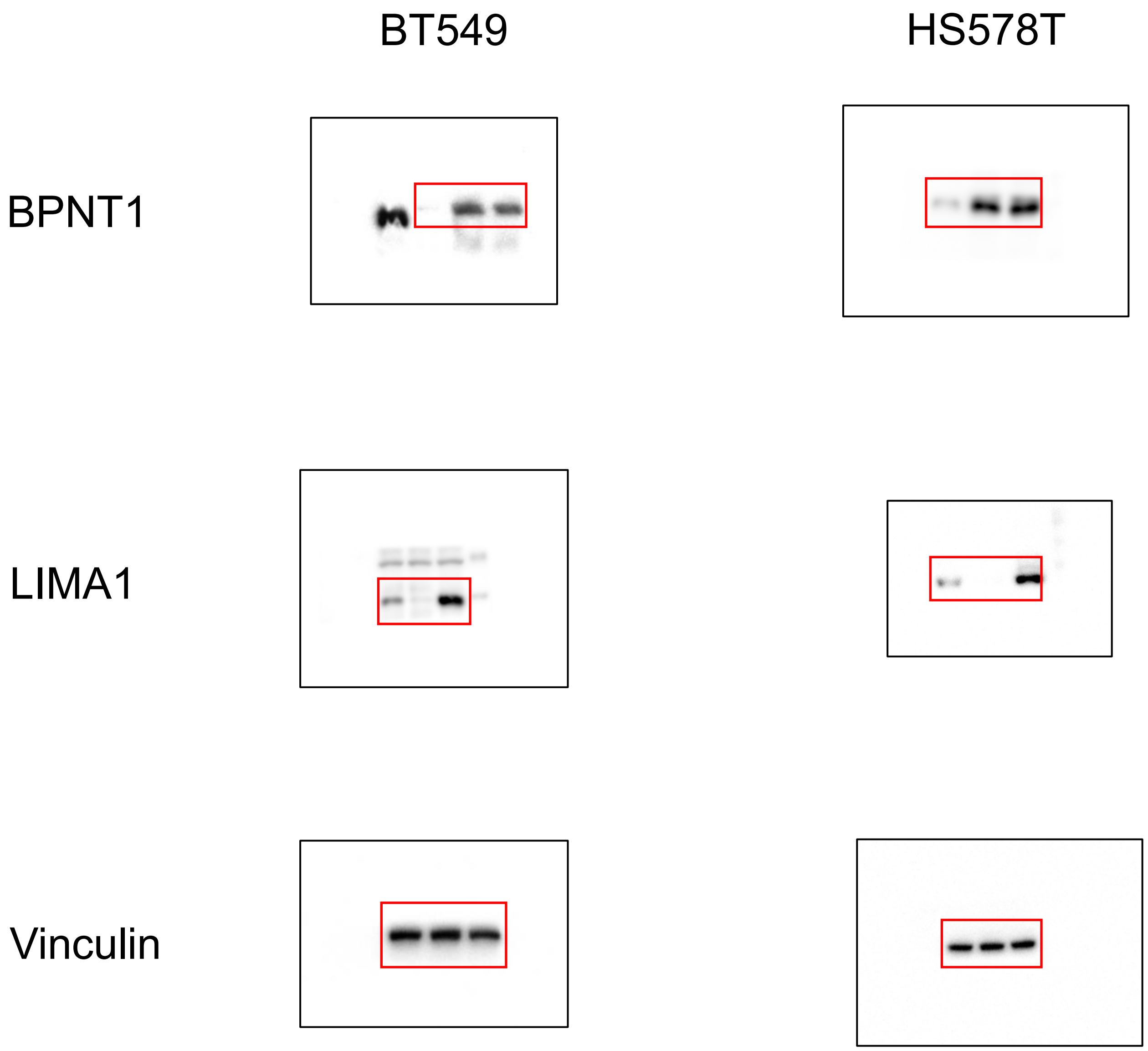

Figure 7A

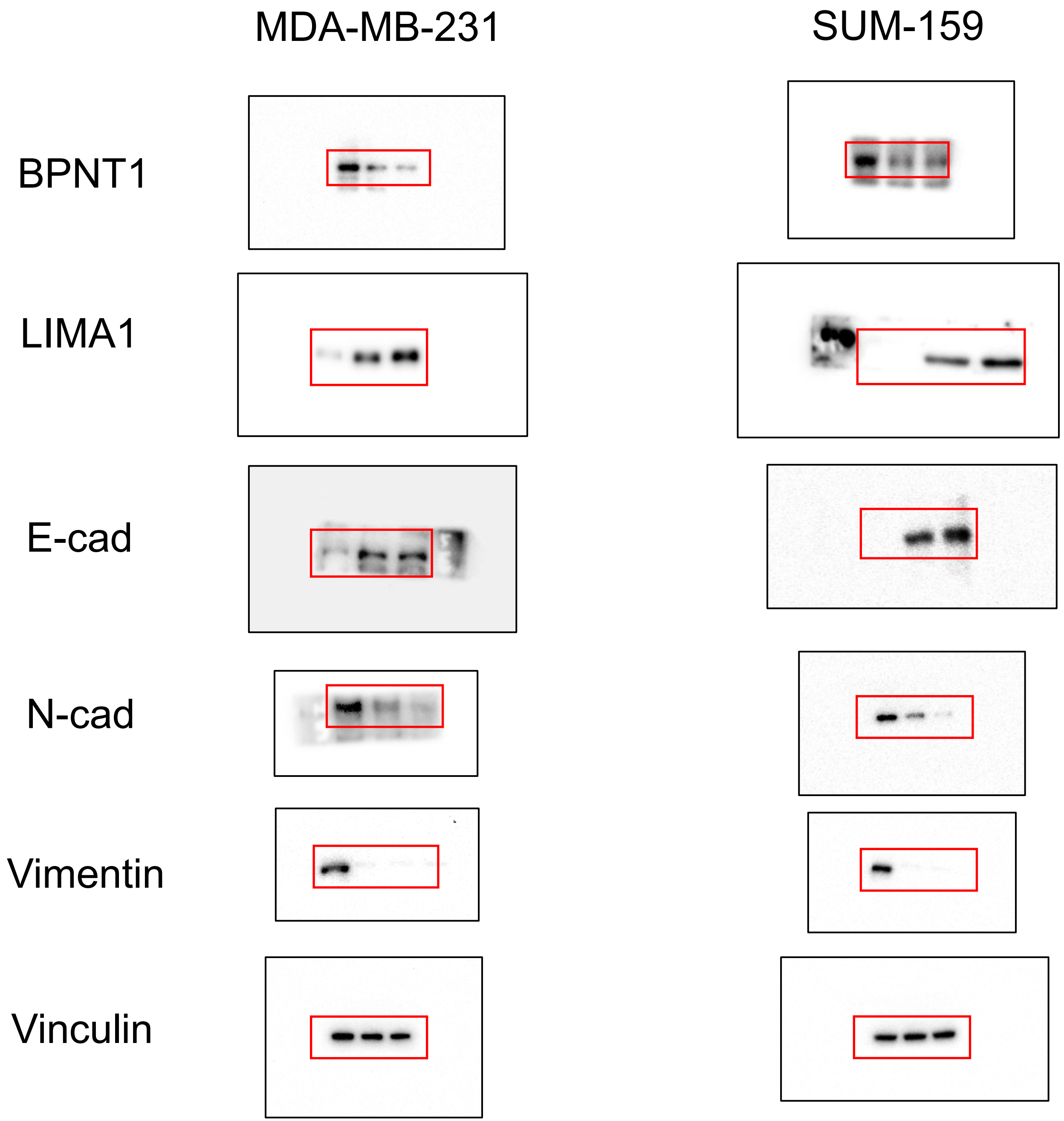

Figure 7B

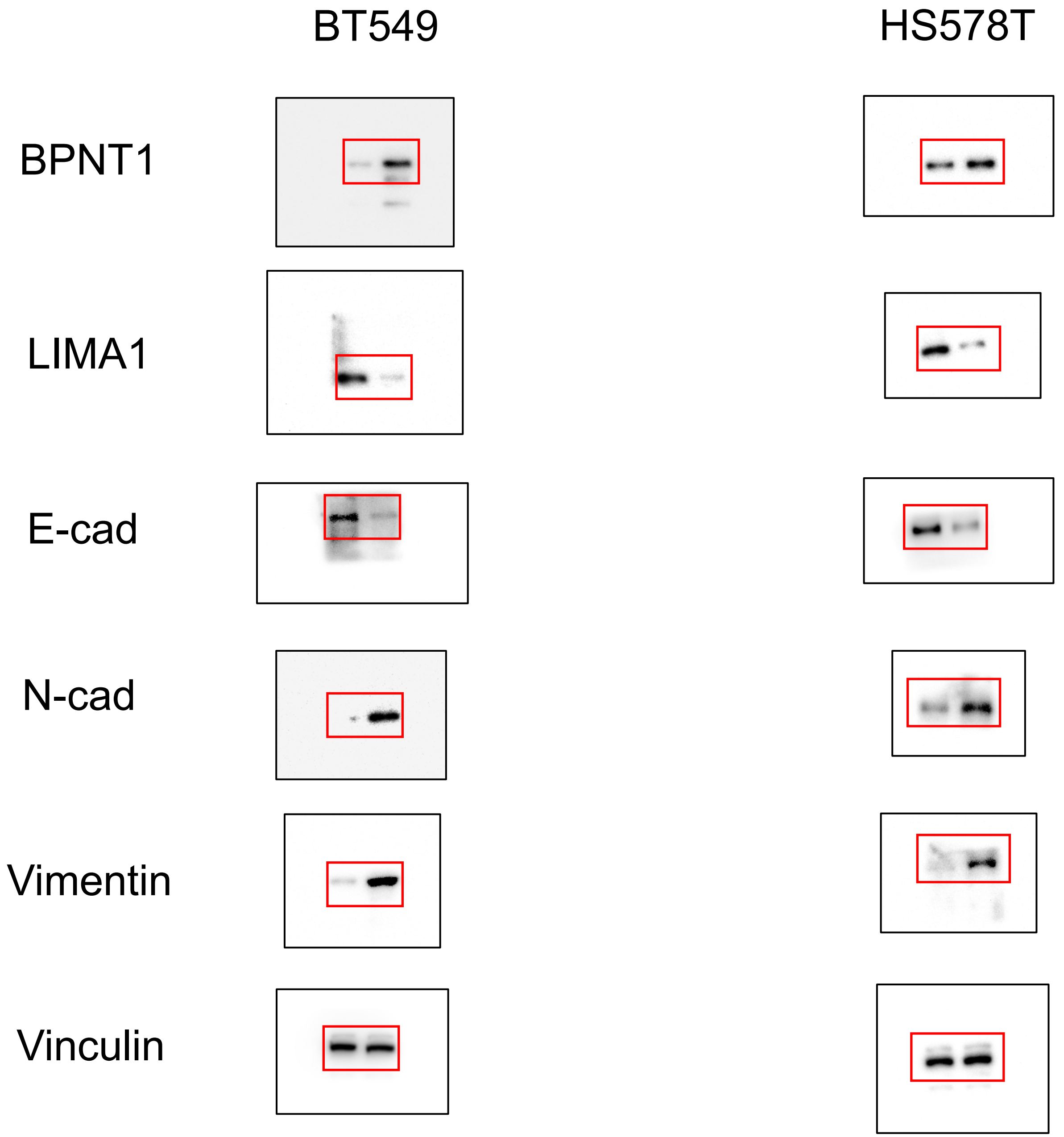

Figure 7C

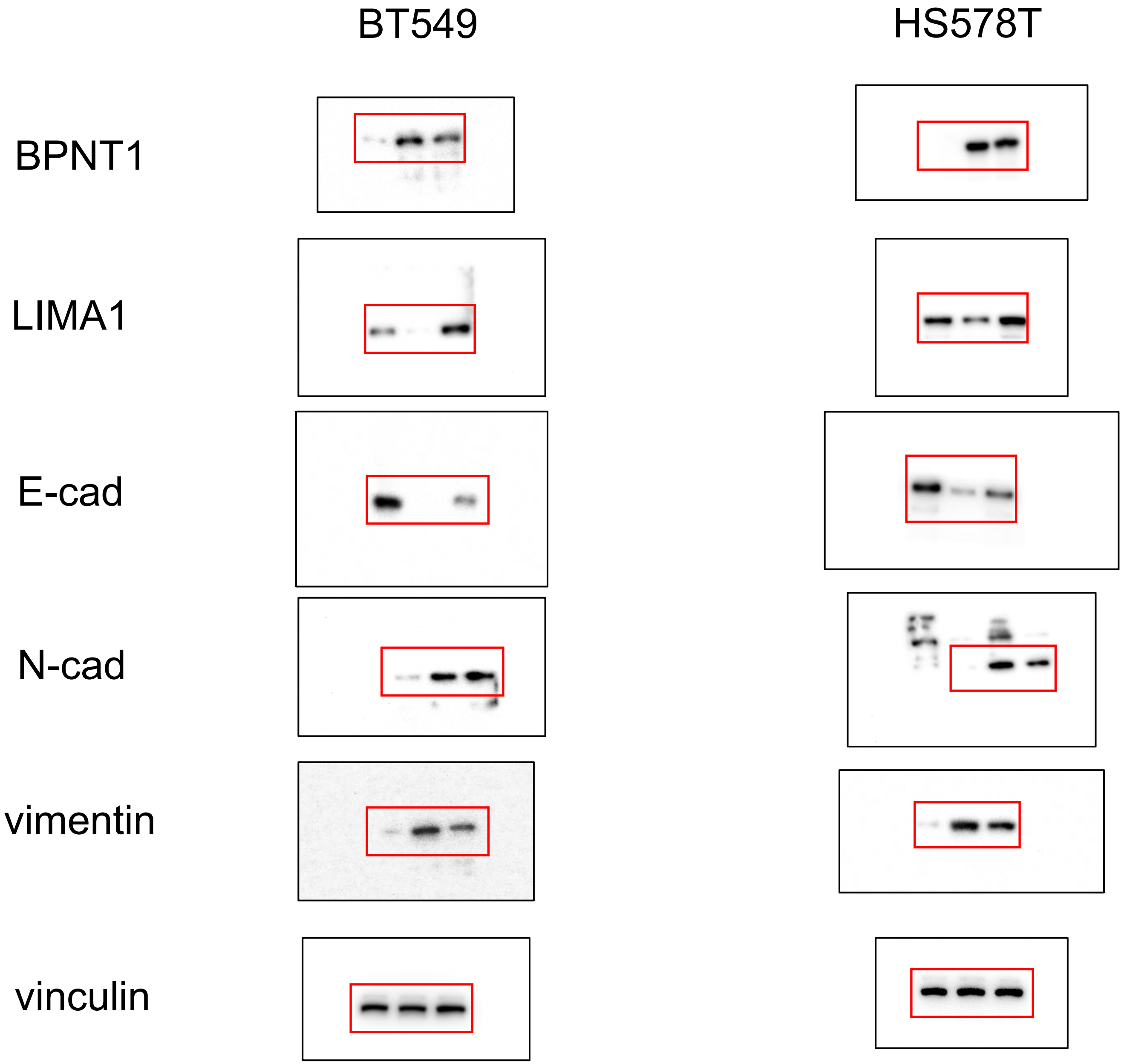

Figure S4B

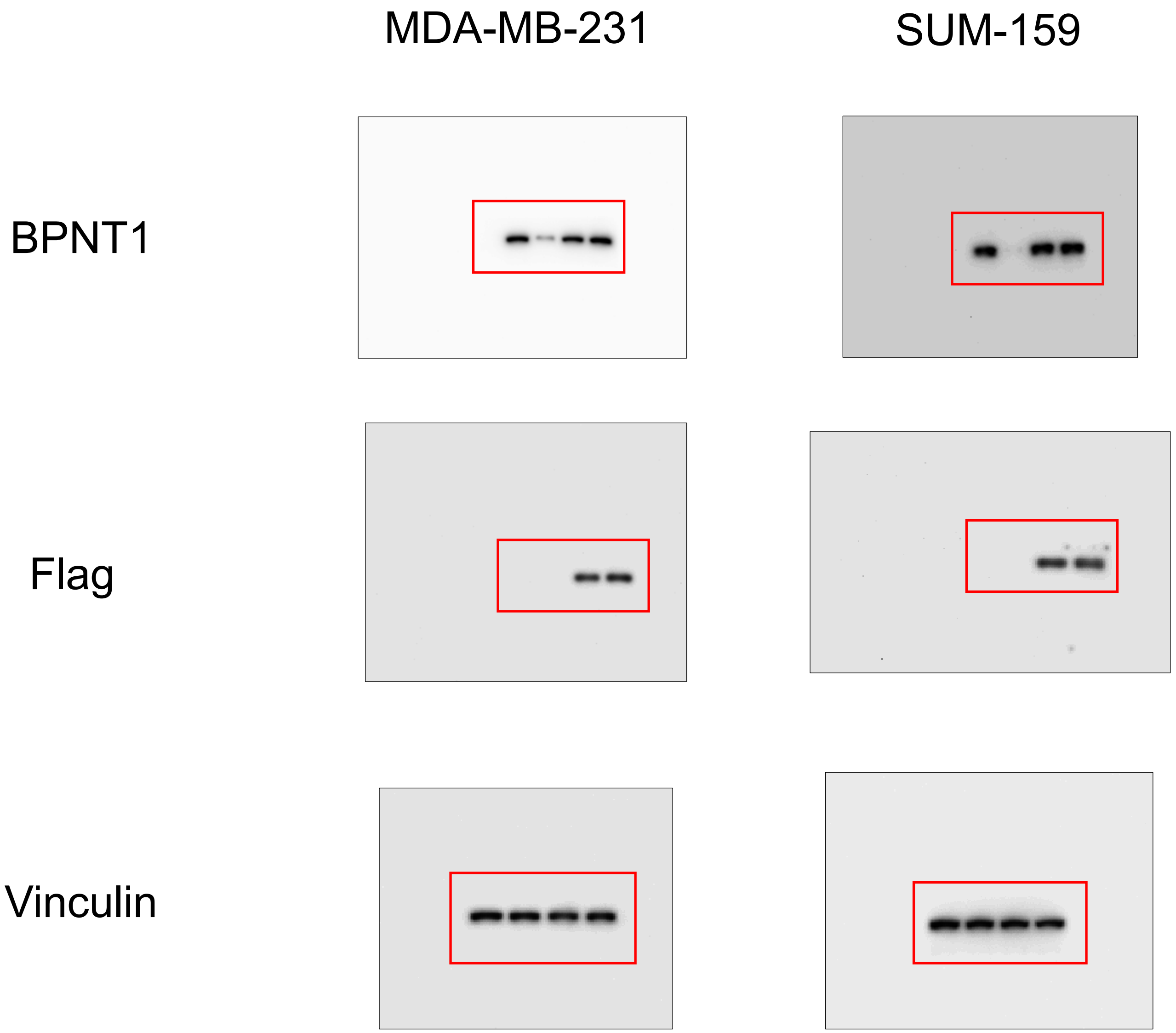

Figure S5A

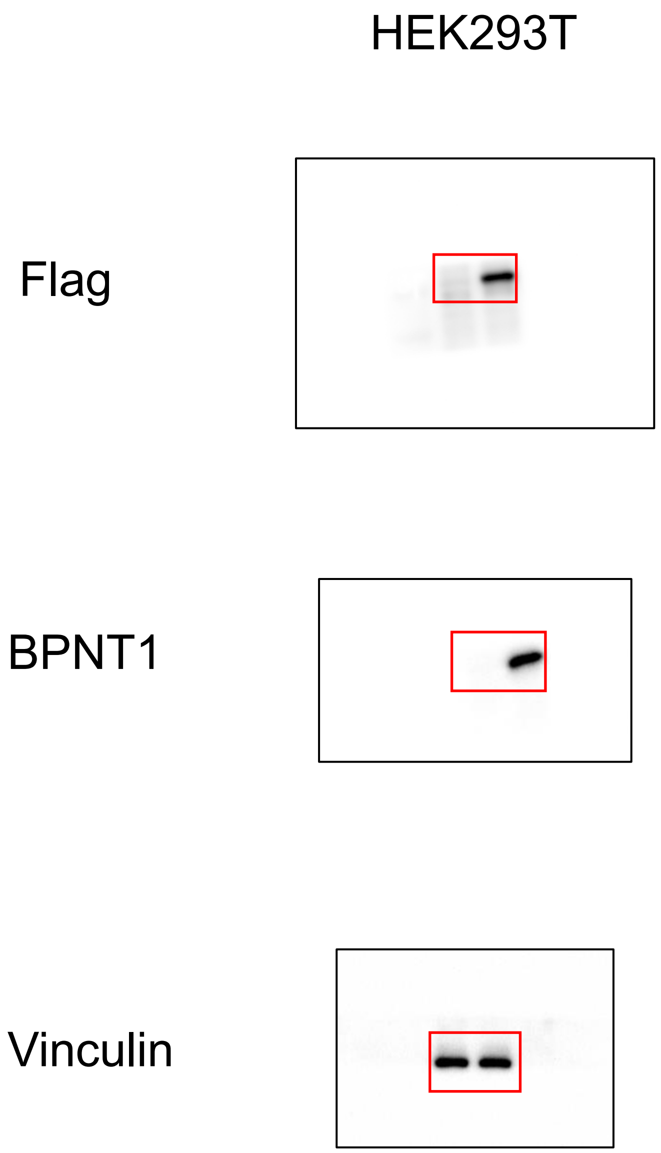

Figure S5G

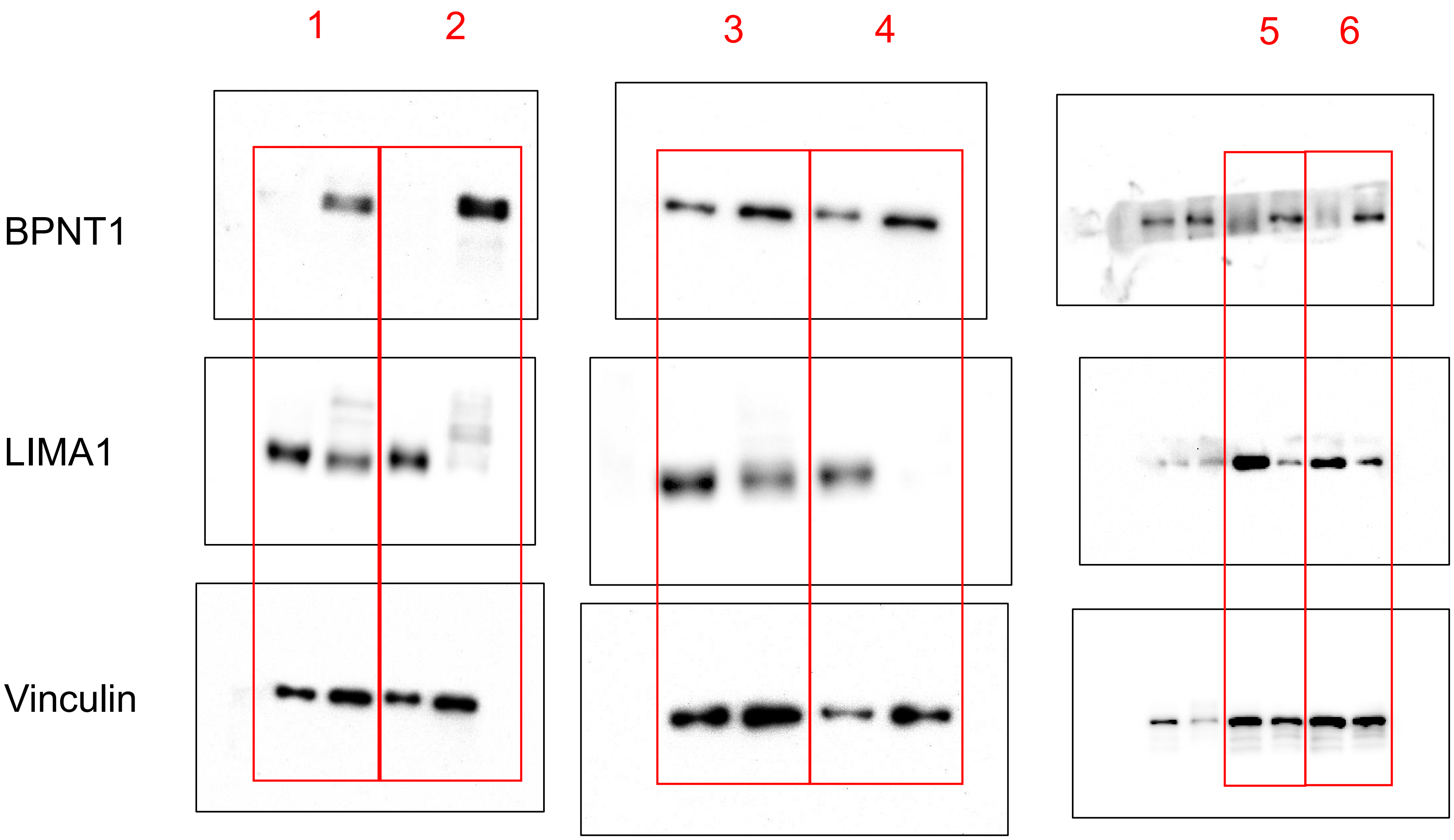

Figure S5G

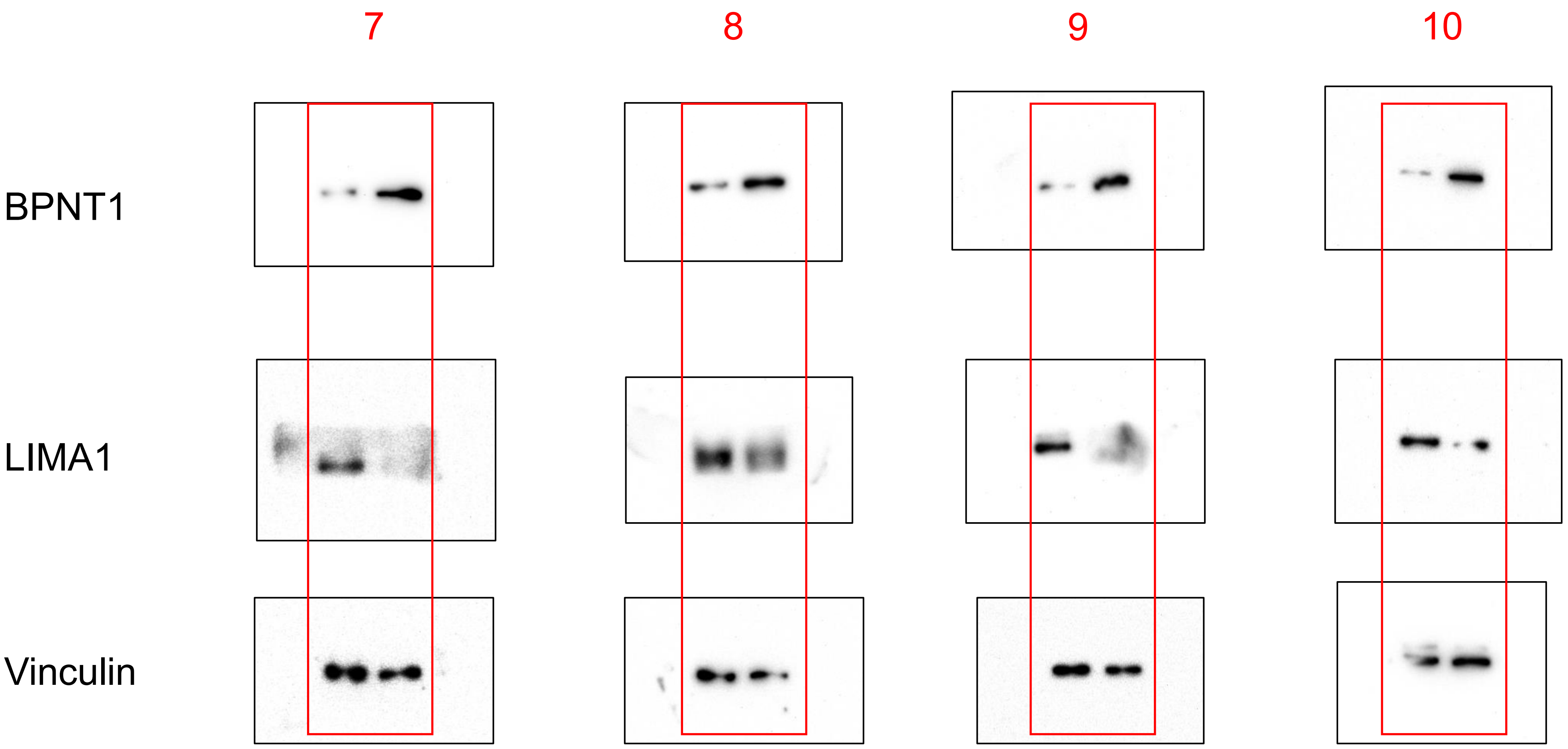

Figure S5J

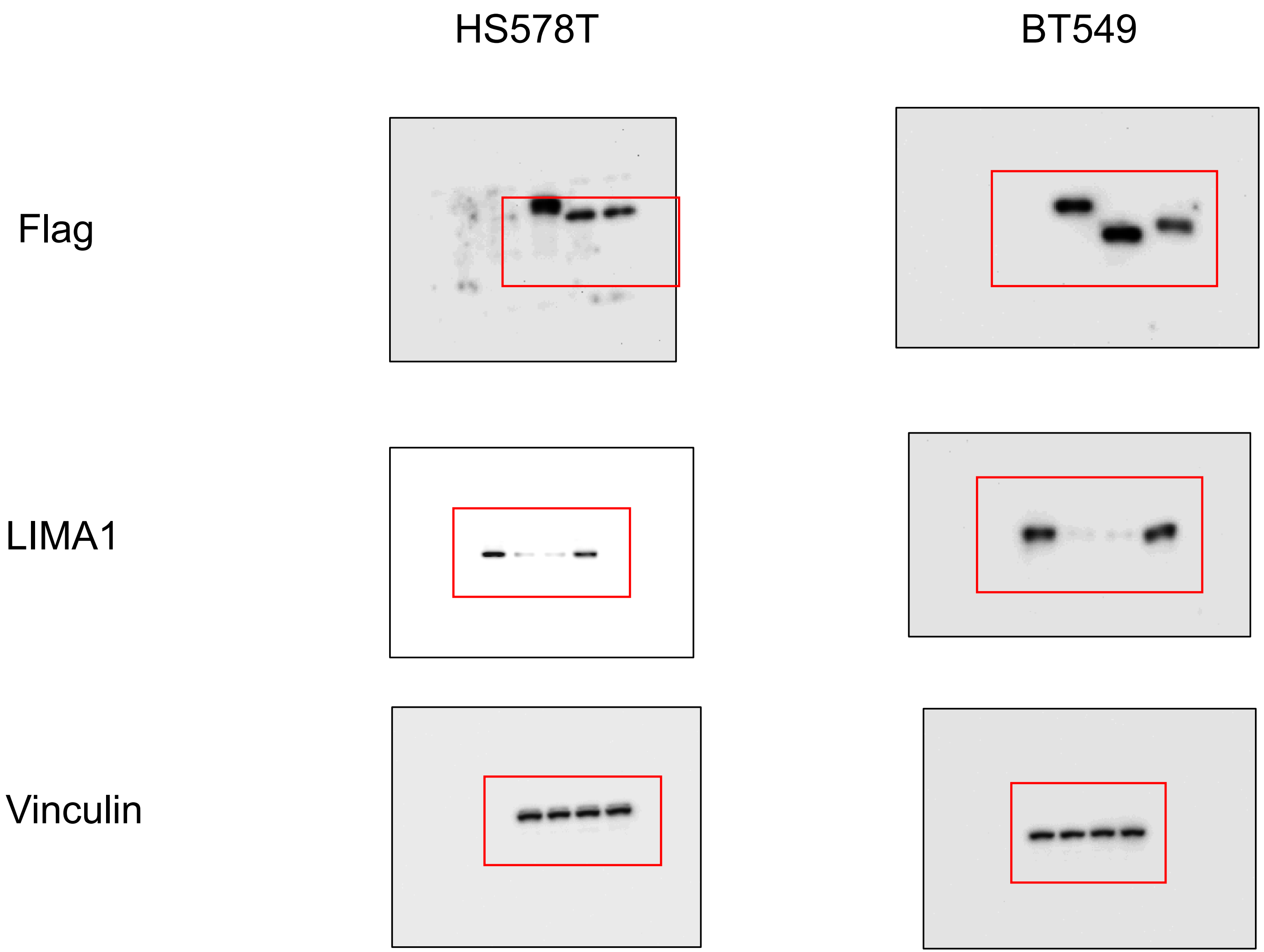

Figure S5K

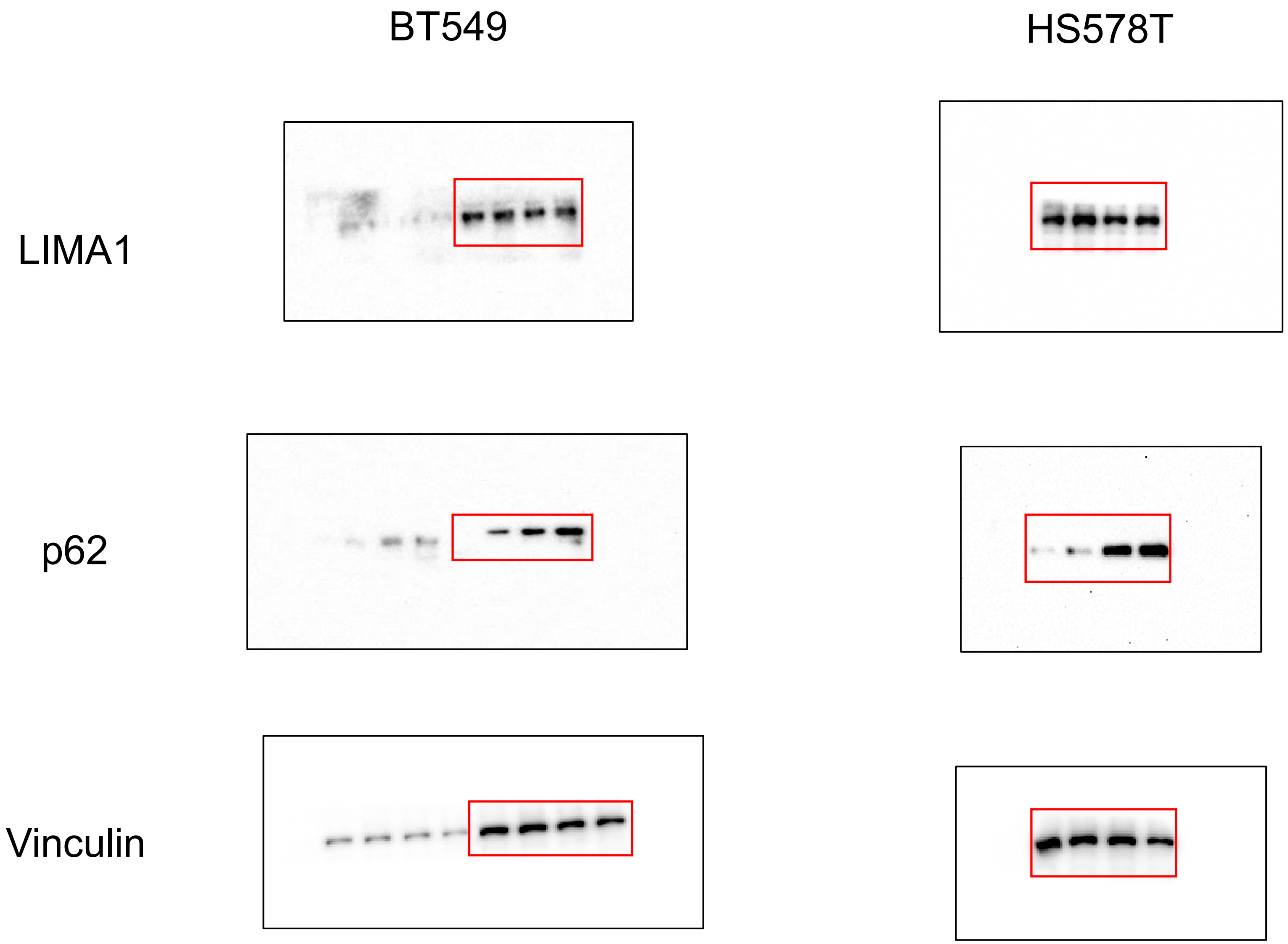

Figure S6A

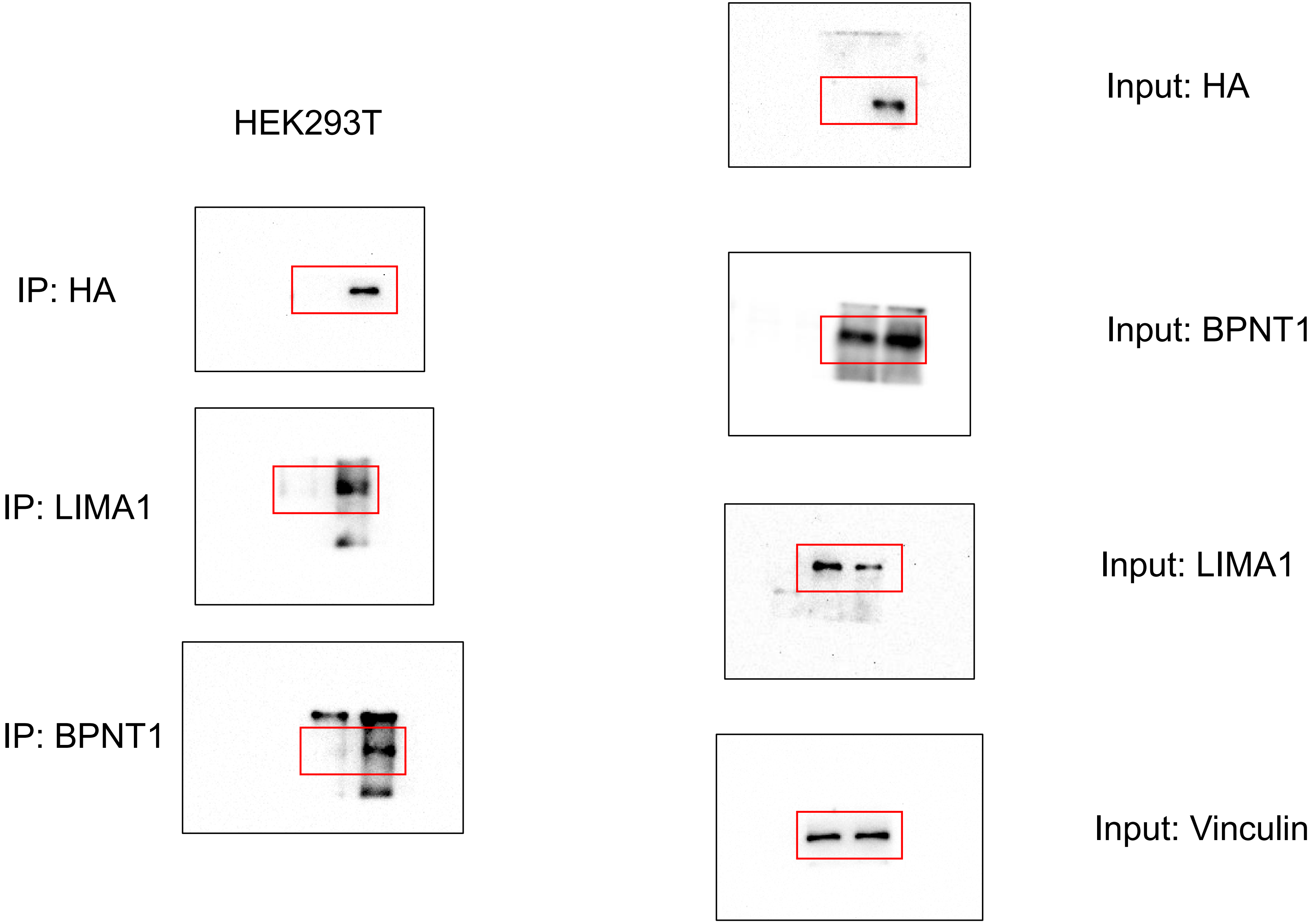

HEK293T
IP: HA
IP: LIMA1
IP: BPNT1
Input: HA
Input: BPNT1
Input: LIMA1
Input: Vinculin

Figure S6B

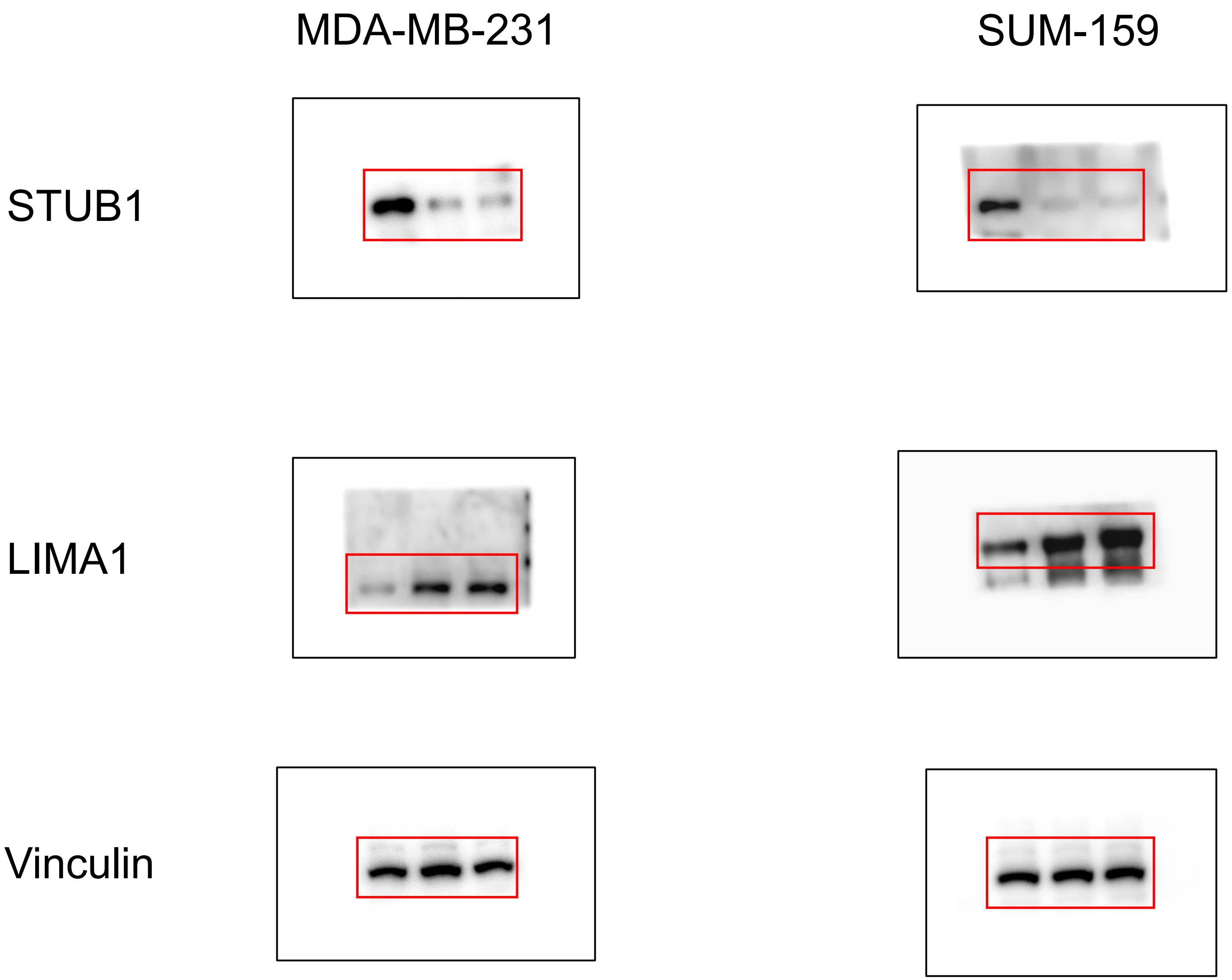

Figure S6C

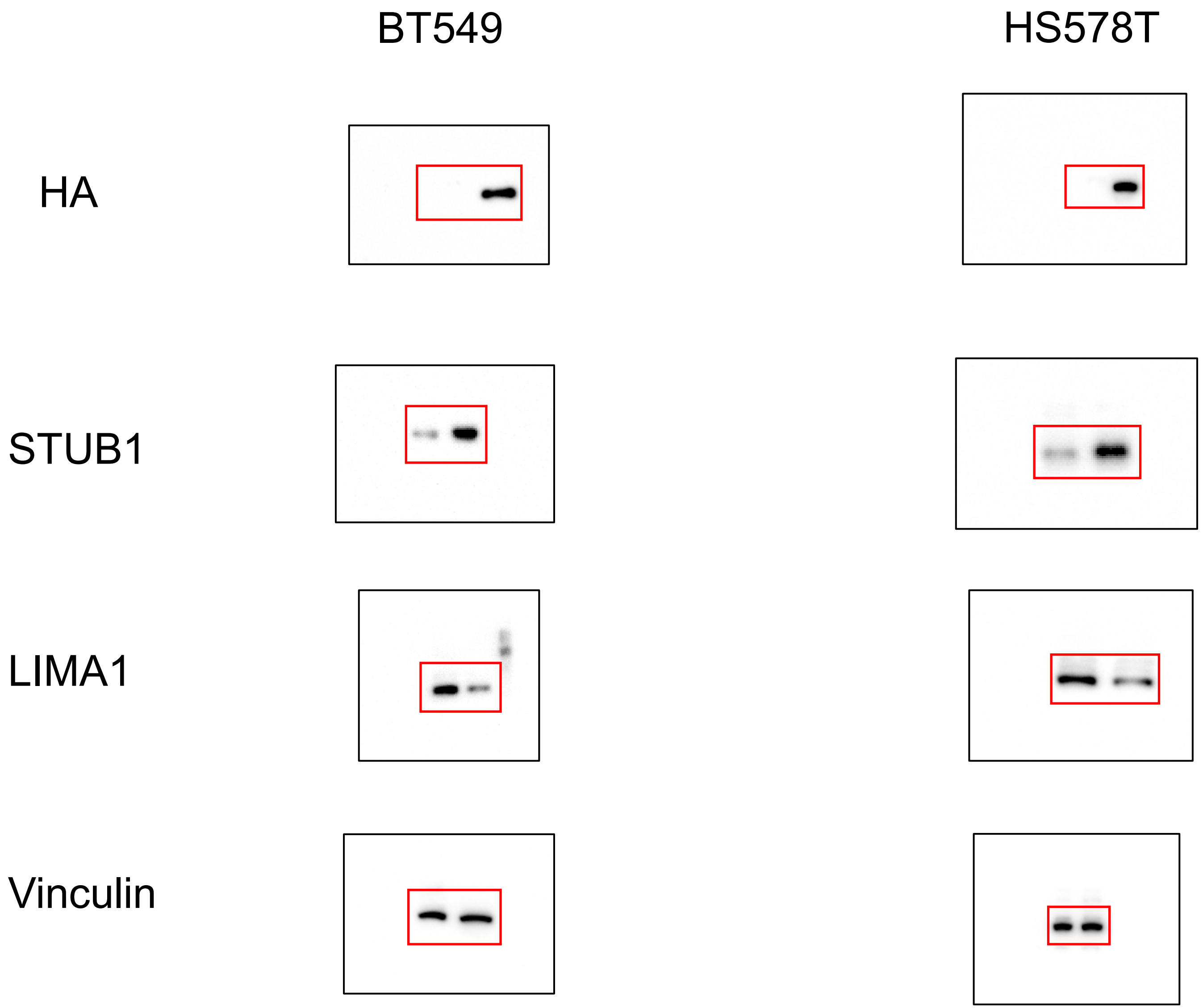

Figure S6F

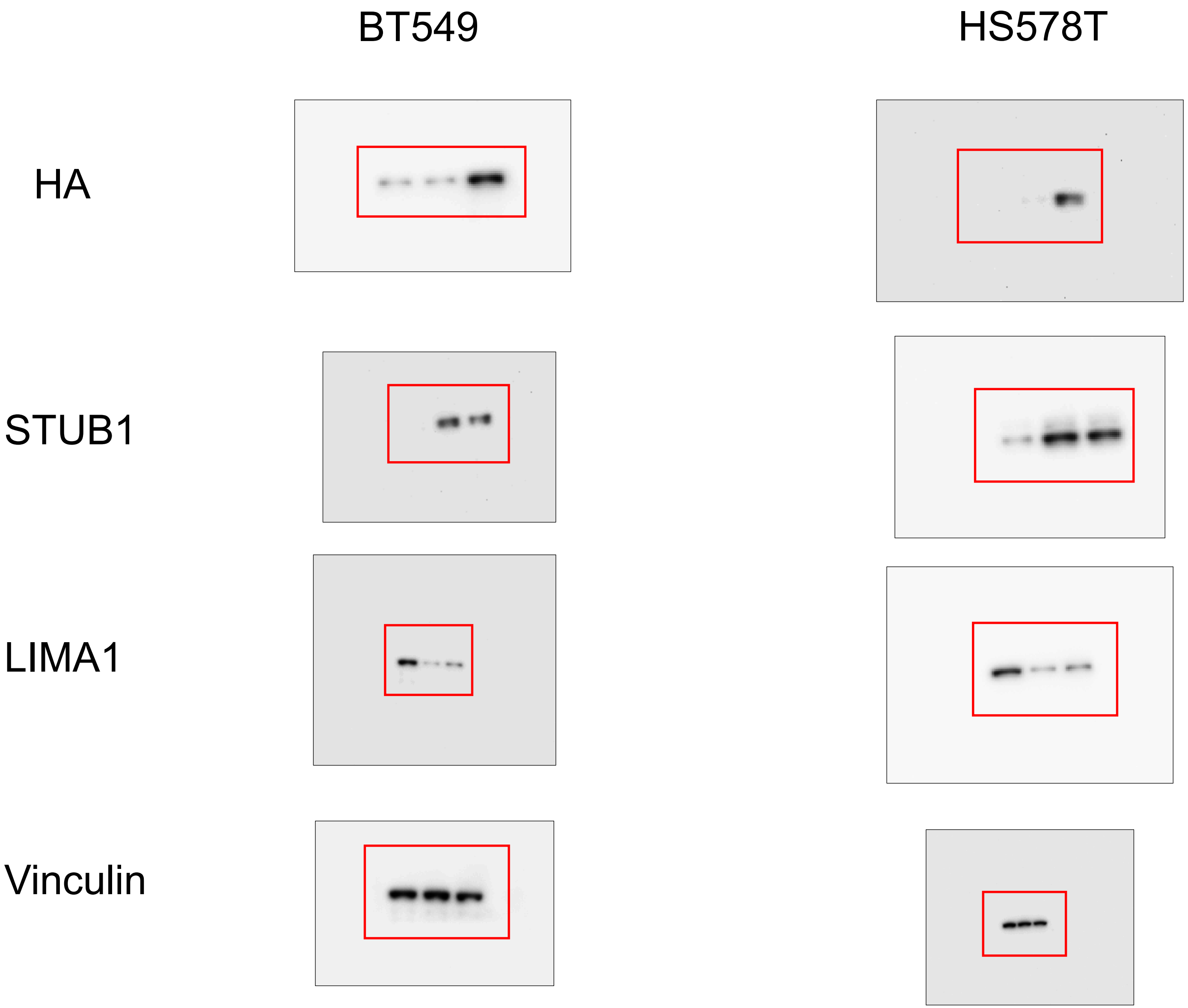

Supplement: Supplementary file 2 — Ling et al. Original western blots 10-28-2025 [file 41419_2025_8245_MOESM2_ESM.pdf]
